# Supplementary material for: mRNA trans-splicing dual AAV vectors for (epi)genome editing and gene therapy
Source: Nat Commun. 2023 Oct 18;14:6578. doi: 10.1038/s41467-023-42386-0 (PMC10584818; doi:10.1038/s41467-023-42386-0)
Supplement: Supplementary file 1 — Supplementary Information [file 41467_2023_42386_MOESM1_ESM.pdf]

Supplementary Information

mRNA trans-splicing dual AAV vectors for (epi)genome editing and gene therapy

Lisa Maria Riedmayr[1], Klara Sonnie Hinrichsmeyer[1], Stefan Bernhard Thalhammer[1], David Manuel Mittas[1], Nina Karguth[1], Dina Yehia Otify[1], Sybille Böhm[2], Valentin Johannes Weber[3], Michael David Bartoschek[2], Victoria Splith[2], Manuela Brümmer[1], Raphael Ferreira[4], Nanda Boon[5], Gabriele Maria Wögenstein[6], Christian Grimm[6], Jan Wijnholds[5,7], Verena Mehlfeld[1], Stylianos Michalakis[8], Stefanie Fenske[1,9], Martin Biel[1], and Elvir Becirovic[3]*

[1]Department of Pharmacy - Center for Drug Research, LMU Munich, Munich, 81377, Germany

[2]ViGeneron GmbH, Planegg, 82152, Germany

[3]Laboratory for Retinal Gene Therapy, Department of Ophthalmology, University Hospital Zurich, University of Zurich, Schlieren, 8952, Switzerland

[4]Genetics Department, Harvard Medical School, Boston, MA 02115, USA

[5]Department of Ophthalmology, Leiden University Medical Center (LUMC), 2333 ZA, Leiden, Netherlands

[6]Laboratory for Retinal Cell Biology, Department of Ophthalmology, University Hospital Zurich, University of Zurich, Schlieren, 8952, Switzerland

[7]Netherlands Institute for Neuroscience, Institute of the Royal Netherlands Academy of Arts and Sciences (KNAW), 1105 BA, Amsterdam, Netherlands

[8]Department of Ophthalmology, University Hospital, LMU Munich, 80336, Munich, Germany

[9]German Center for Cardiovascular Research (DZHK), partner site Munich Heart Alliance, Munich, 81377, Germany

* Correspondence: Elvir Becirovic, Email: elvir.becirovic@uzh.ch

Inventory of Supporting Information

Supplementary Figures

1. Fig. S1 *In vitro* evaluation of REVeRT.
2. Fig. S2 Reconstitution of fluorescent reporter genes via dual REVeRT AAVs *in vivo*.
3. Fig. S3 Split luciferase reporter assay.
4. Fig. S4 *Myo7b* transactivation using split dCas9-VPR reconstituted via REVeRT.
5. Fig. S5 *In vitro* prime editing and CONNACT strategy via REVeRT.
6. Fig. S6 Natural history in Abca4-deficient mice.
7. Fig. S7. Functional analysis of REVeRT- and buffer-injected eyes in Abca4$^{-/-}$ Rdh8$^{-/-}$ mice.
8. Fig. S8 Pairwise comparison of treated and buffer-injected eyes in Abca4-deficient mice.
9. Fig. S9 Gating Strategy used for flow cytometry on HEK293 cells transfected with split cerulean constructs.

Supplementary Tables

1. Table S1. Binding domain analysis.
2. Table S2. Trans-splicing elements used for REVeRT.
3. Table S3. Differentially expressed genes.
4. Table S4 Splicing events with endogenous transcripts in Cas9-VPR injected retinas.
5. Table S5 sgRNA sequences.
6. Table S6 pegRNA sequences used in Fig. S5.
7. Table S7 Primer sequences.

Supplementary Notes

1. Supplementary Note 1: Sequences of REVeRT plasmids for therapeutic purposes.

Supplementary Figures

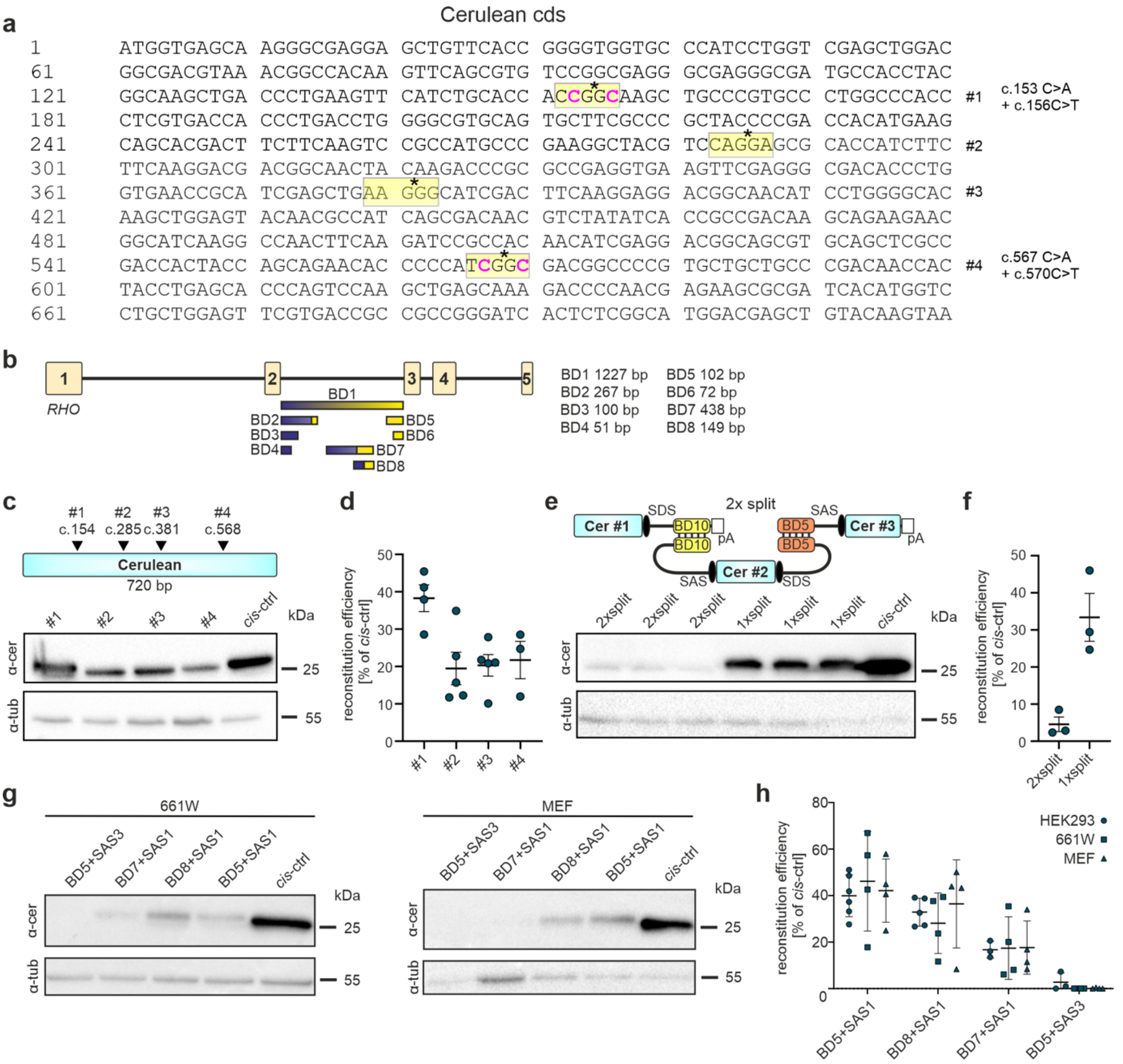

Fig. S1 *In vitro* evaluation of REVeRT. **a** Coding sequence (cds) of cerulean. The split sites (#1-4) used are highlighted in yellow. Asterisks mark the exact split position. Introduced silent mutations are highlighted in pink. **b** Scheme depicting *RHO* intron 2 used for BD1-BD8. The respective sequences originate from different regions of the intron as indicated by the color gradient. BD2, BD7 and BD8 represent fusion sequences containing the parts of the 5' end and parts of the 3' end of the intron. **c** Upper panel, Position of the split sites within the cerulean sequence. Lower panel, Western Blot of HEK293 cells transfected with cerulean vectors split at positions #1-4. **d** Ratiometric quantification of reconstitution efficiency relative to the cis-ctrl determined from western blots **c**. n=4 (#1), n=5 (#2, #3) and n=3 (#4) transfections. **e** Upper panel, Scheme depicting mRNA reconstitution for cerulean split into three fragments. Lower panel, Western Blot of HEK293 cells transfected with cerulean constructs split into two (1xsplit) or three fragments (2xsplit). **f** Ratiometric quantification of reconstitution efficiency relative to the cis-ctrl determined from western blots **e**. n=3 transfections. **g** Western blot of 661W (left) or mouse embryonic fibroblast (MEF, right) cells transfected with split cerulean constructs in presence of different BDs or SASs. **h** Ratiometric quantification of the reconstitution efficiency relative to the cis-ctrl determined from western blots **g**. The corresponding HEK293 western blots are shown in Fig 1. Scatter plots show mean ± SEM. Minimum of n=3 transfections. All source data are provided as a Source Data file.

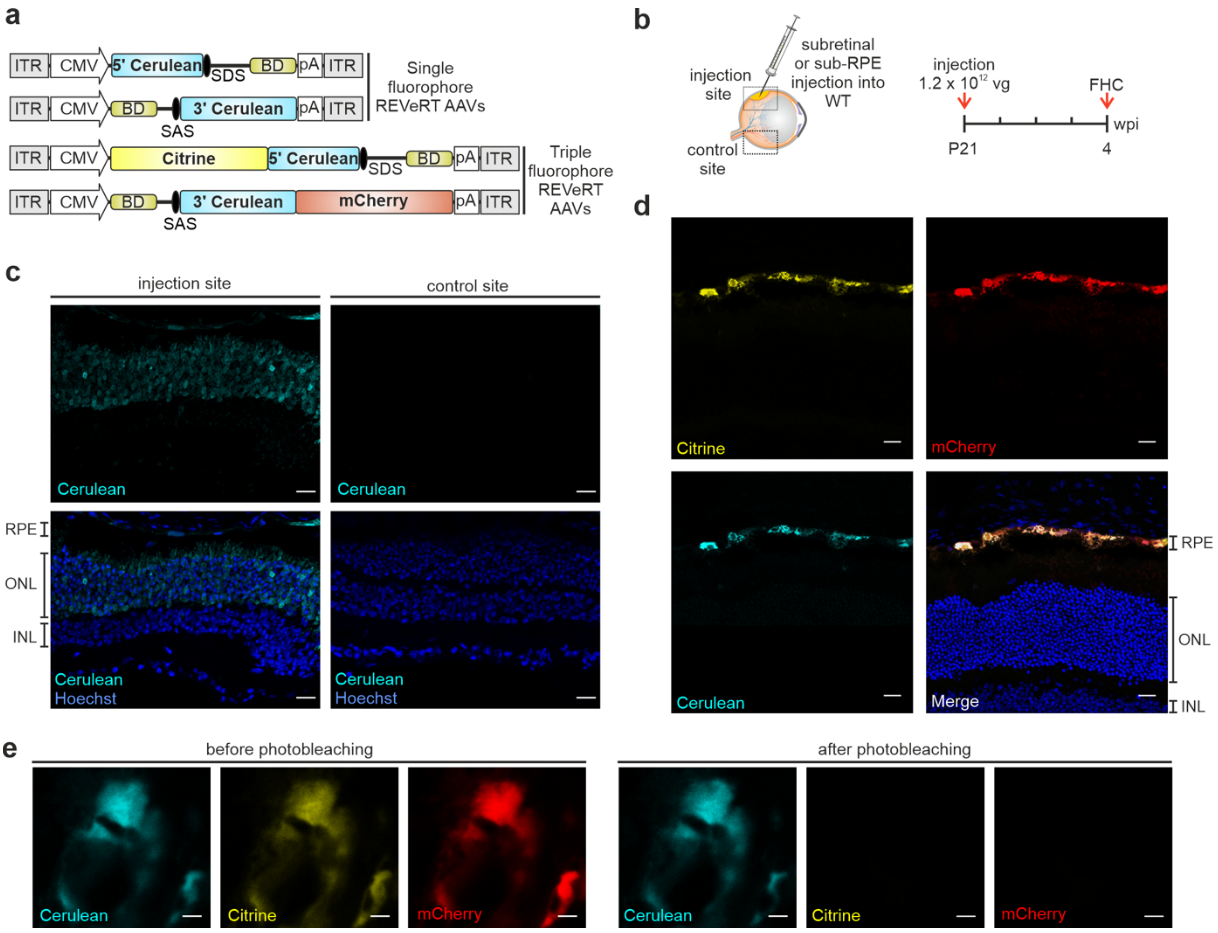

Fig. S2 Reconstitution of fluorescent reporter genes via dual REVeRT AAVs *in vivo*. a Design of dual REVeRT AAVs used for experiments shown in **c** (single fluorophore), **d** and **e** (triple fluorophore). ITR, inverted terminal repeats. **b** Experimental setup. FHC, fluorescence histochemistry. wpi, weeks post injection. **c** Representative FHC images of retinas obtained from mice injected with single fluorophore REVeRT AAVs. Left panel, cerulean expression along the injection site; Right panel, sections from the non-injected control site within the same retina. RPE, retinal pigment epithelium; ONL, outer nuclear layer; INL, inner nuclear layer. Scale bar, 20 µm. **d**, **e** FHC of a retina injected with triple fluorophore REVeRT AAVs (citrine, split cerulean and mCherry) system. Scale bar, 30 µm. **e** High magnification images of RPE cell expressing all three fluorophores before and after selective photobleaching of the mCherry and citrine signal. Photobleaching confirmed the presence of reconstituted cerulean. Scale bar, 2 µm. All results in **c-e** are derived from non-amplified (i.e., antibody-free) fluorophore signals.

a Luciferase cds

```
1     ATGGAAGACG CCAAAAACAT AAAGAAAGGC CCGGCGCCAT TCTATCCGCT GGAAGATGGA ACCGCTGGAG
71    AGCAACTGCA TAAGGCTATG AAGAGATACG CCCTGGTTCC TGGAACAATT GCTTTTACAG ATGCACATAT
141   CGAGGTGGAC ATCACTTACG CTGAGTACTT CGAAATGTCC GTTCGGTTGG CAGAAGCTAT GAAACGATAT
211   GGGCTGAATA CAAATCACAG AATCGTCGTA TGCAGTGAAA ACTCTCTTCA ATTCTTTATG CCGGTGTTGG
281   GCGCGTTATT TATCGGAGTT GCAGTTGCGC CCGCGAACGA CATTTATAAT GAACGTGAAT TGCTCAACAG
351   TATGGGCATT TCGCAGCCTA CCGTGGTGTT CGTTTCCAAA AAGGGGTTGC AAAAAATTTT GAACGTGCAA
421   AAAAAGCTCC CAATCATCCA AAAAATTATT ATCATGGATT CTAAAACGGA TTACCAGGGA TTTCAGTCGA
491   TGTACACGTT CGTCACATCT CATCTACCTC CCGGTTTTAA TGAATACGAT TTTGTGCCAG AGTCCTTCGA
561   TAGGGACAAG ACAATTGCAC TGATCATGAA CTCCTCTGGA TCTACTGGTC TGCCTAAAGG TGTCGCTCTG
631   CCTCATAGAA CTGCCTGCGT GAGATTCTCG CATGCCAGAG ATCCTATTTT TGGCAATCAA ATCATTCCGG
701   ATACTGCGAT TTTAAGTGTT GTTCCATTCC ATCACGGTTT TGGAATGTTT ACTACACTCG GATATTTGAT
771   ATGTGGATTT CGAGTCGTCT TAATGTATAG ATTTGAAGAA GAGCTGTTTC TGAGGAGCCT TCAGGATTAC   #1 c.834
841   AAGATTCAAA GTGCGCTGCT GGTGCCAACC CTATTCTCCT TCTTCGCCAA AAGCACTCTG ATTGACAAAT
911   ACGATTTATC TAATTTACAC GAAATTGCTT CTGGTGGCGC TCCCCTCTCT AAGGAAGTCG GGGAAGCGGT   #2 c.963
981   TGCCAAGAGG TTCCATCTGC CAGGTATCAG GCAAGGATAT GGGCTCACTG AGACTACATC AGCTATTCTG   #3 c.1003
1051  ATTACACCCG AGGGGGATGA TAAACCGGGC GCGGTCGGTA AAGTTGTTCC ATTTTTTGAA GCGAAGGTTG
1121  TGGATCTGGA TACCGGGAAA ACGCTGGGCG TTAATCAAAG AGGCGAACTG TGTGTGAGAG GTCCTATGAT
1191  TATGTCCGGT TATGTAAACA ATCCGGAAGC GACCAACGCC TTGATTGACA AGGATGGATG GCTACATTCT
1261  GGAGACATAG CTTACTGGGA CGAAGACGAA CACTTCTTCA TCGTTGACCG CCTGAAGTCT CTGATTAAGT
1331  ACAAAGGCTA TCAGGTGGCT CCCGCTGAAT TGGAATCCAT CTTGCTCCAA CACCCCAACA TCTTCGACGC
1401  AGGTGTCGCA GGTCTTCCCG ACGATGACGC CGGTGAACTT CCCGCCGCCG TTGTTGTTTT GGAGCACGGA
1471  AAGACGATGA CGGAAAAAGA GATCGTGGAT TACGTCGCCA GTCAAGTAAC AACCGCGAAA AAGTTGCGCG
1541  GAGGAGTTGT GTTTGTGGAC GAAGTACCGA AAGGTCTTAC CGGAAAACTC GACGCAAGAA AAATCAGAGA
1611  GATCCTCATA AAGGCCAAGA AGGGCGGAAA GATCGCCGTG TAA
```

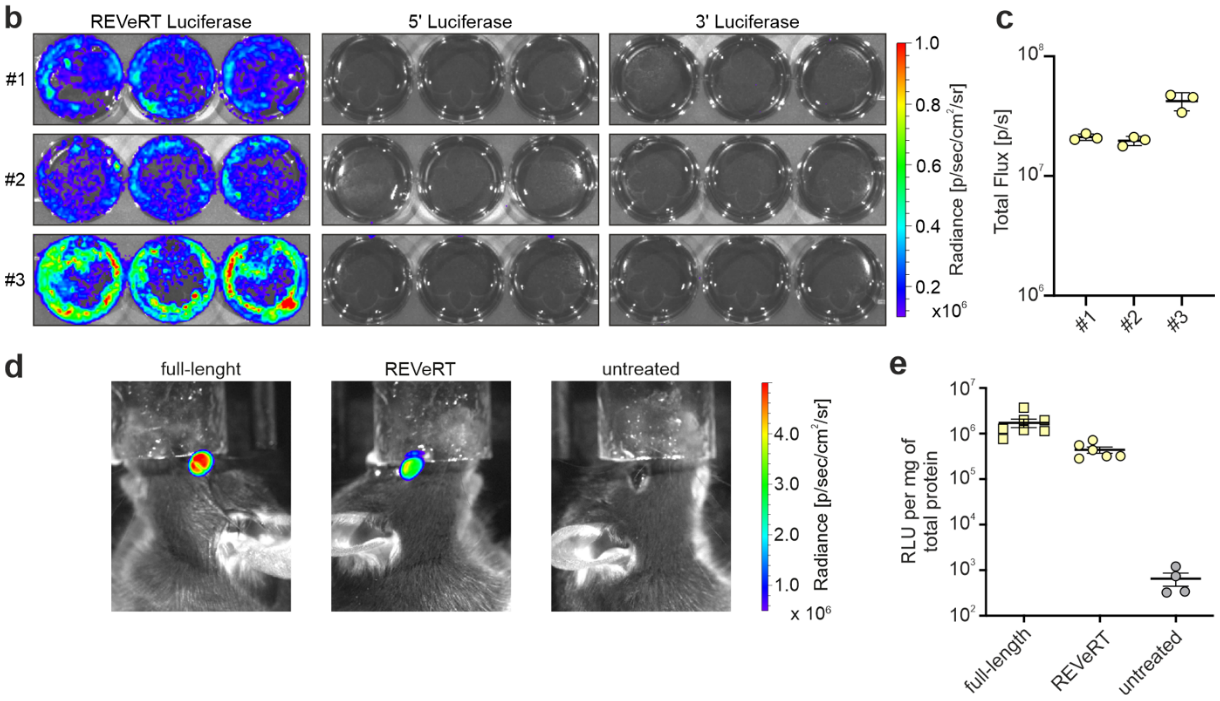

Fig. S3 Split luciferase reporter assay. a Luciferase coding sequence (cds). The split sites (#1-#3) used in this study are highlighted in yellow. Asterisks mark the exact split position. **b** Luminescence images of HEK293T cells transfected with luciferase constructs split at different positions as indicated. The cells were either transfected with dual vectors (REVeRT Luciferase) or with a single vector (5' Luciferase, 3' Luciferase) as negative control. **c** Quantification of the results shown in **b**. Scatter plot shows mean ± SEM. n=3 transfections. **d** Luminescence images of mice subretinally injected with split (REVeRT) or full-length luciferase constructs at three weeks post injection. **e** Relative quantification of luciferase expression in injected retinas at three weeks post injection. RLU, relative light units. Plots show mean ± SEM. n=7 retinas (full-length), n=6 retinas (REVeRT), n=4 retinas (untreated). All source data are provided as a Source Data file.

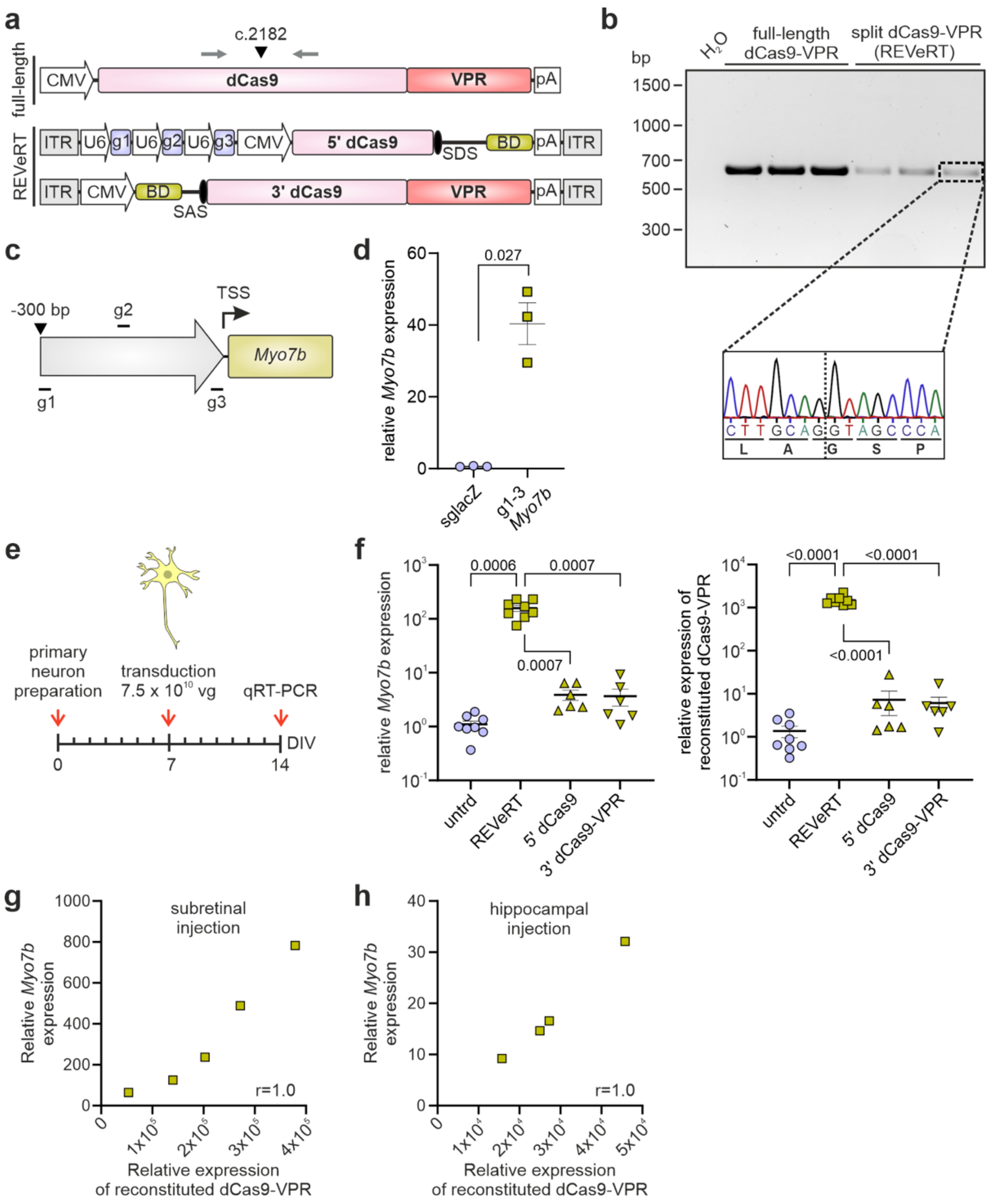

Fig. S4 ***Myo7b*** **transactivation using split dCas9-VPR reconstituted via REVeRT. a** Split (REVeRT) and full-length dCas9-VPR constructs used in **b-h**. Split site (arrowhead) and junction-spanning primers (arrows) are indicated. **b** RT-PCR using indicated primer pair. Sequencing of the splicing junction shown below. The RT-PCR was repeated twice. **c** Binding positions of three sgRNAs targeting the region upstream of the transcription start site (TSS) of *Myo7b*. **d** qRT-PCR from 661W cells co-transfected with split dCas9-VPR and either *lacZ*- or *Myo7b*-targeting (g1-g3) sgRNAs. *lacZ*-transfected cells served as reference. Mann-Whitney test was used for statistical analysis. n=3 independent transfections. **e** Time scale for experiments in mouse primary neurons transduced with dual REVeRT AAV8Y733F vectors. DIV, days *in vitro*. **f** Relative expression of *Myo7b* and reconstituted dCas9-VPR in transduced mouse primary neurons (n=8 cultures). Welch ANOVA with Dunnett T3 was used for statistical analysis. Untreated cells (untrd) served as reference (n=8 cultures). Neurons transduced with single vectors (5' dCas9, 3' dCas9-VPR) served as controls (n=6 cultures)**. g**, **h** Dose-dependency between dCas9-VPR reconstitution and transcriptional activation of *Myo7b* upon subretinal (**g**) or hippocampal injection (**h**). Spearman correlation coefficient shown in the corner. Scatter plots show mean ± SEM. All source data are provided as a Source Data file.

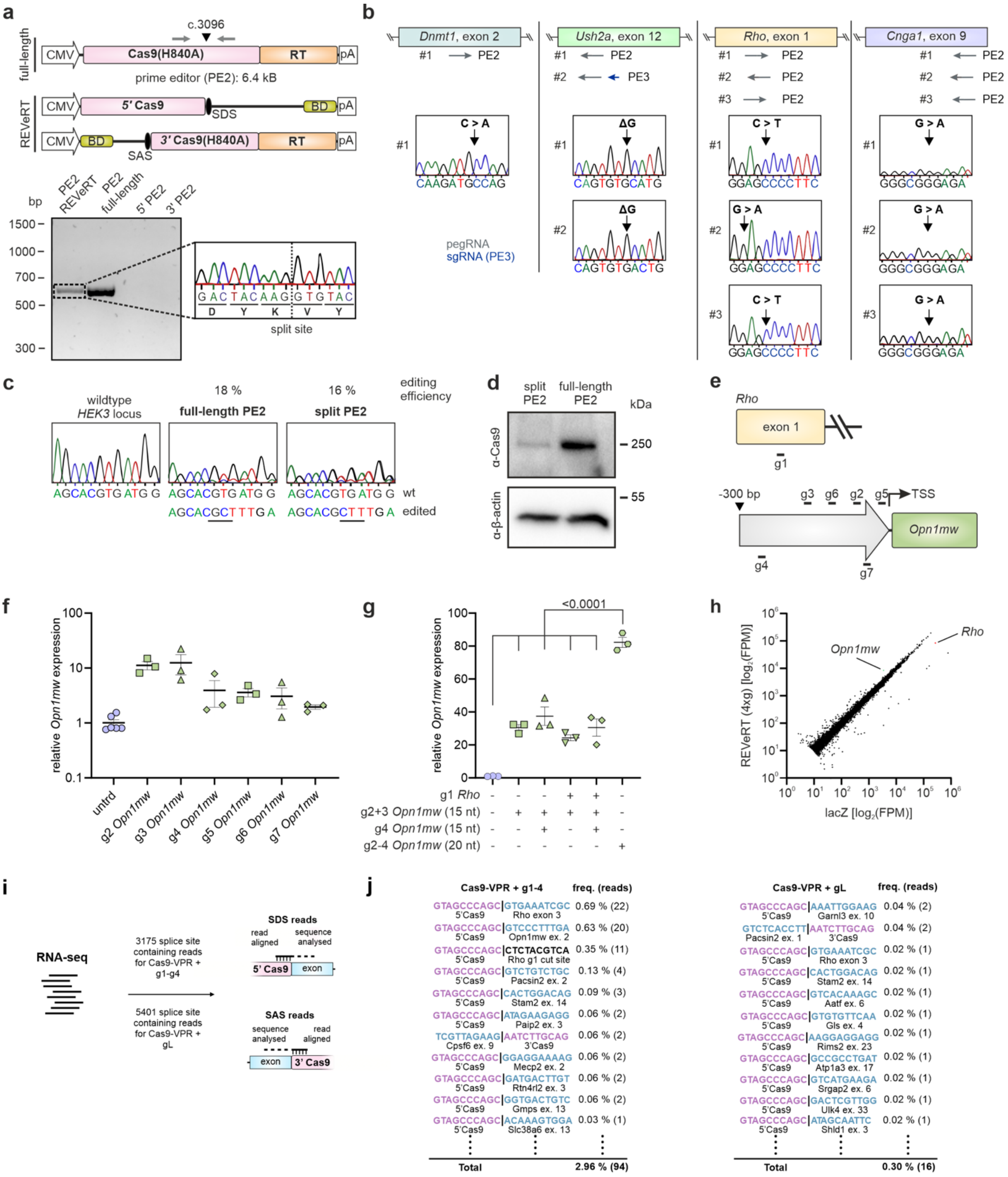

Fig. S5 ***In vitro*** **prime editing and CONNACT strategy via REVeRT. a** Upper panel, split and full-length prime editor 2 (PE2) constructs used in **b** and **c**. Split site (arrowhead) and junction-spanning primers (arrows) are indicated. Lower panel, RT-PCR of reconstituted PE2 using junction-spanning primers and sequencing result of the splicing junction. Single vectors (5’ PE2 and 3’ PE2) served as controls. The RT-PCR has been repeated three times. **b** Sequencing results of the targeted genomic loci after co-transfection of 661W and MEF cells with PE2 or PE3 (PE2 + additional sgRNA introducing a nick) and corresponding pegRNAs. Target exons and binding position of the pegRNAs are depicted above. **c** Sequencing of the *HEK3* locus after co-transfection of HEK293T cells with split or full-length PE2 and pegRNA. **d** Western blot of 661W cells transfected with split or full-length PE. The western blot has been repeated once. **e** Binding positions of the sgRNAs targeting *Rho* exon 1 (g1) or the region upstream of the TSS of *Opn1mw* (g2-g7). **f** qRT-PCR from MEF cells co-transfected with

dCas9-VPR and g2 – g7. Untreated cells (untrd) served as reference. n=3 independent transfections. **g** qRT-PCR from MEF cells co-transfected with dCas9-VPR and different combinations of sgRNAs as indicated. Spacer lengths of *Opn1mw*-targeting sgRNAs are shown in brackets. Untreated cells served as reference. One-way ANOVA with Dunnett's T3 test was used to compare expression levels using a 15 nt vs. 20 nt spacer. Scatter plots show mean ± SEM. n=3 independent transfections. **h** RNA-Seq analysis of retinas originating from three different animals (dark pink data points in Fig. 4d) injected with REVeRT(4xg) or *lacZ* vectors. *Opn1mw* and *Rho* transcripts are highlighted. FPM, fragments per million. **i** Schematic depiction of RNA-Seq data analysis to identify REVeRT splicing events with endogenous transcripts. **j** Identity of transcripts containing a splicing event with an endogenous mRNA. Splicing events have been analyzed for retinas injected with Cas9-VPR and targeting sgRNAs (g1-g4) and injected with a non-targeting sgRNA (gL). The full list of identified sequences can be found in Table S4. All source data are provided as a Source Data file.

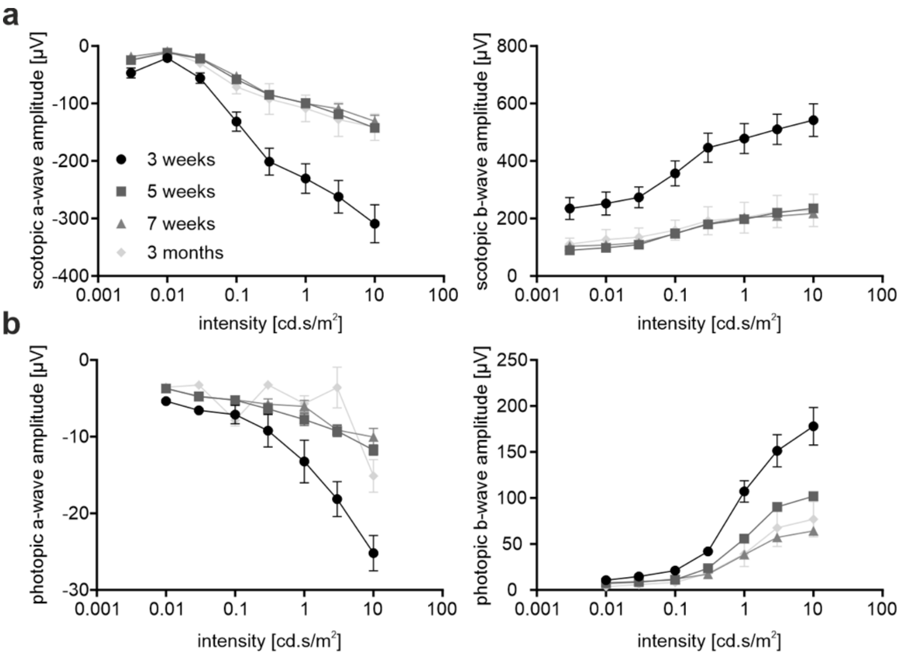

Fig. S6 Natural history in Abca4-deficient mice. a, **b** Scotopic (**a**) and photopic (**b**) ERG measurements for untreated *Abca4*$^{-/-}$/*Rdh8*$^{-/-}$ mice between 3 weeks and 3 months of age as indicated. Plots show mean ± SEM. n=4 mice. Both eyes were measured. All source data are provided as a Source Data file.

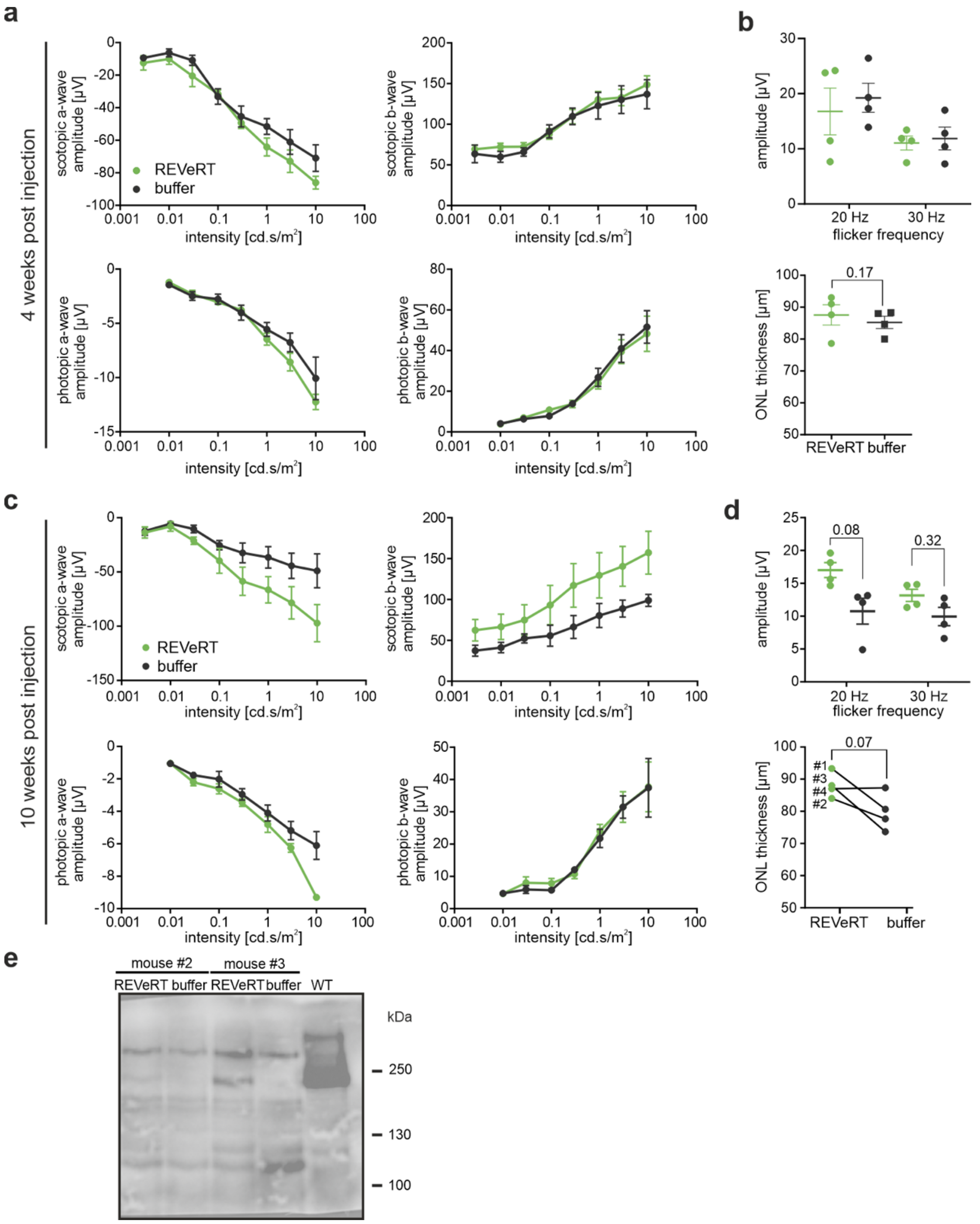

Fig. S7. Functional analysis of REVeRT- and buffer-injected eyes in Abca4$^{-/-}$ Rdh8$^{-/-}$ mice. **a** ERG measurements of injected *Abca4$^{-/-}$/Rdh8$^{-/-}$* mice at 4 wpi under scotopic (upper panel) and photopic (lower panel) conditions. **b** Upper panel, Amplitudes from flicker stimuli at 20 Hz and 30 Hz under photopic conditions at 4 wpi. Lower panel, OCT measurements of injected *Abca4$^{-/-}$/Rdh8$^{-/-}$* mice at 4 wpi. **c** ERG measurements under scotopic (upper panel) or photopic (lower panel) conditions at 10 wpi. **d** Upper panel, Results from flicker at 20 Hz and 30 Hz under photopic conditions at 10 wpi. Lower panel, Pairwise comparison of OCT measurements for the treated and control eye of the single animals at 10 wpi. Two-way ANOVA with Šídák's multiple comparisons test was used for statistical analysis. Plots show mean ± SEM. Wpi, weeks post injection. n=4 injected mice for **a** – **d**. **e** Uncropped western blot shown in Fig. 5e. All source data are provided as a Source Data file.

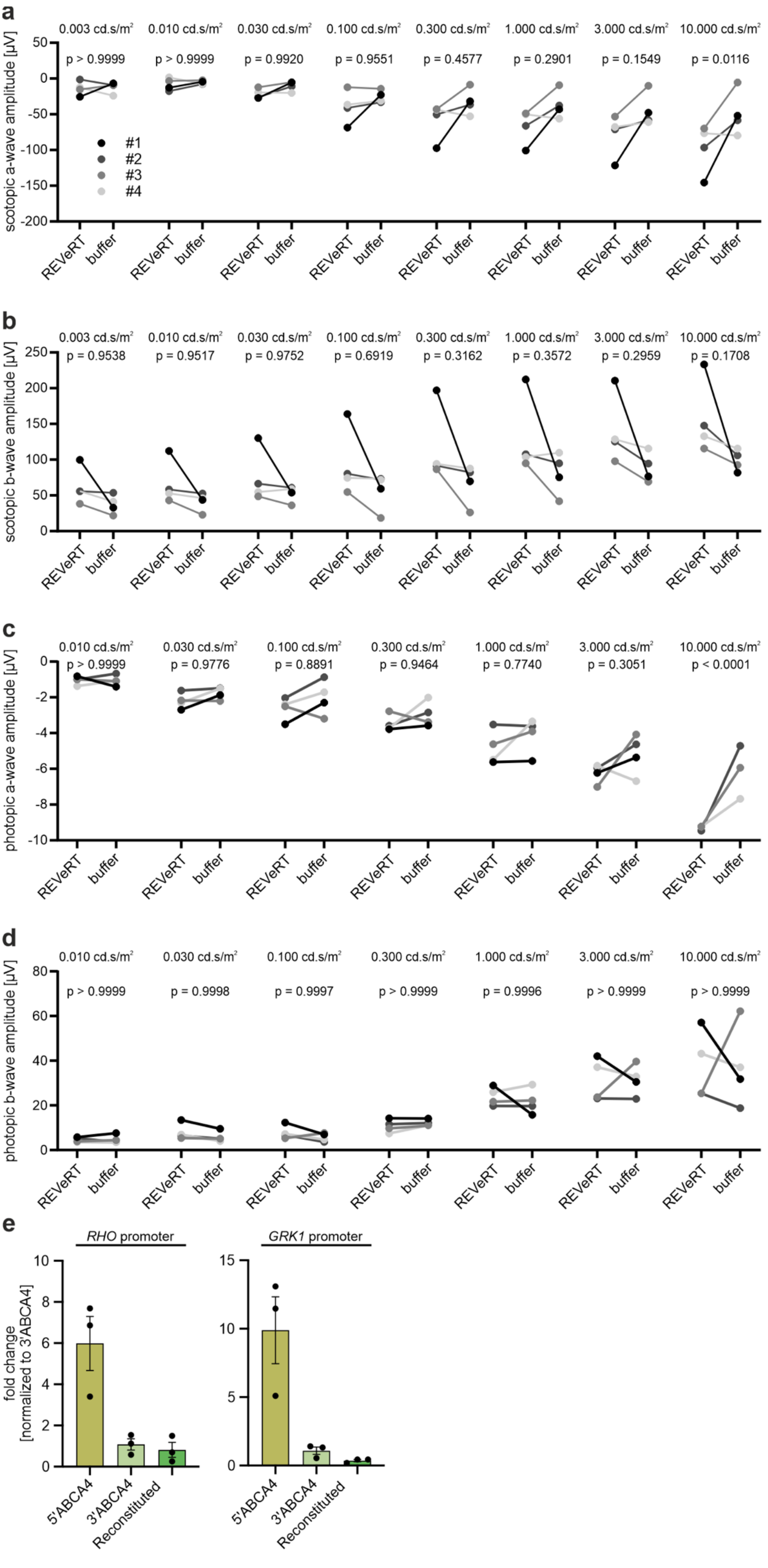
a
0.003 cd.s/m²
0.010 cd.s/m²
0.030 cd.s/m²
0.100 cd.s/m²
0.300 cd.s/m²
1.000 cd.s/m²
3.000 cd.s/m²
10.000 cd.s/m²
p > 0.9999
p > 0.9999
p = 0.9920
p = 0.9551
p = 0.4577
p = 0.2901
p = 0.1549
p = 0.0116
scotopic a-wave amplitude [µV]
50
0
-50
-100
-150
-200
#1
#2
#3
#4
REVeRT
buffer
b
0.003 cd.s/m²
0.010 cd.s/m²
0.030 cd.s/m²
0.100 cd.s/m²
0.300 cd.s/m²
1.000 cd.s/m²
3.000 cd.s/m²
10.000 cd.s/m²
p = 0.9538
p = 0.9517
p = 0.9752
p = 0.6919
p = 0.3162
p = 0.3572
p = 0.2959
p = 0.1708
scotopic b-wave amplitude [µV]
250
200
150
100
50
0
REVeRT
buffer
c
0.010 cd.s/m²
0.030 cd.s/m²
0.100 cd.s/m²
0.300 cd.s/m²
1.000 cd.s/m²
3.000 cd.s/m²
10.000 cd.s/m²
p > 0.9999
p = 0.9776
p = 0.8891
p = 0.9464
p = 0.7740
p = 0.3051
p < 0.0001
photopic a-wave amplitude [µV]
0
-2
-4
-6
-8
-10
REVeRT
buffer
d
0.010 cd.s/m²
0.030 cd.s/m²
0.100 cd.s/m²
0.300 cd.s/m²
1.000 cd.s/m²
3.000 cd.s/m²
10.000 cd.s/m²
p > 0.9999
p = 0.9998
p = 0.9997
p > 0.9999
p = 0.9996
p > 0.9999
p > 0.9999
photopic b-wave amplitude [µV]
80
60
40
20
0
REVeRT
buffer
e
RHO promoter
GRK1 promoter
fold change
[normalized to 3'ABCA4]
10
8
6
4
2
0
15
10
5
0
5'ABCA4
3'ABCA4
Reconstituted

Fig. S8 Pairwise comparison of treated and buffer-injected eyes in Abca4-deficient mice. **a**-**d** Pairwise plots for the ERG measurements shown in Fig. S7a and c under scotopic (**a**, **b**) or photopic (**c**, **d**) conditions as indicated. Light intensities and p-values (two-way ANOVA with Šídák's multiple comparison test) are shown above the corresponding measurements. n=4 injected mice. **e** Relative frequency of 5'ABCA4 mRNA, 3'ABCA4 mRNA and reconstituted mRNA in REVeRT-injected retinas of adult *Abca4*$^{-/-}$/*Rdh8*$^{-/-}$ mice 4 weeks post injection. All values are relative to the frequency of 3'ABCA4 transcript. Transcript expression was driven by a RHO (left) or a GRK1 promoter (right). Error bars indicate mean ± standard error and were calculated from technical triplicate reactions from n=3 injected mice. All source data are provided as a Source Data file.

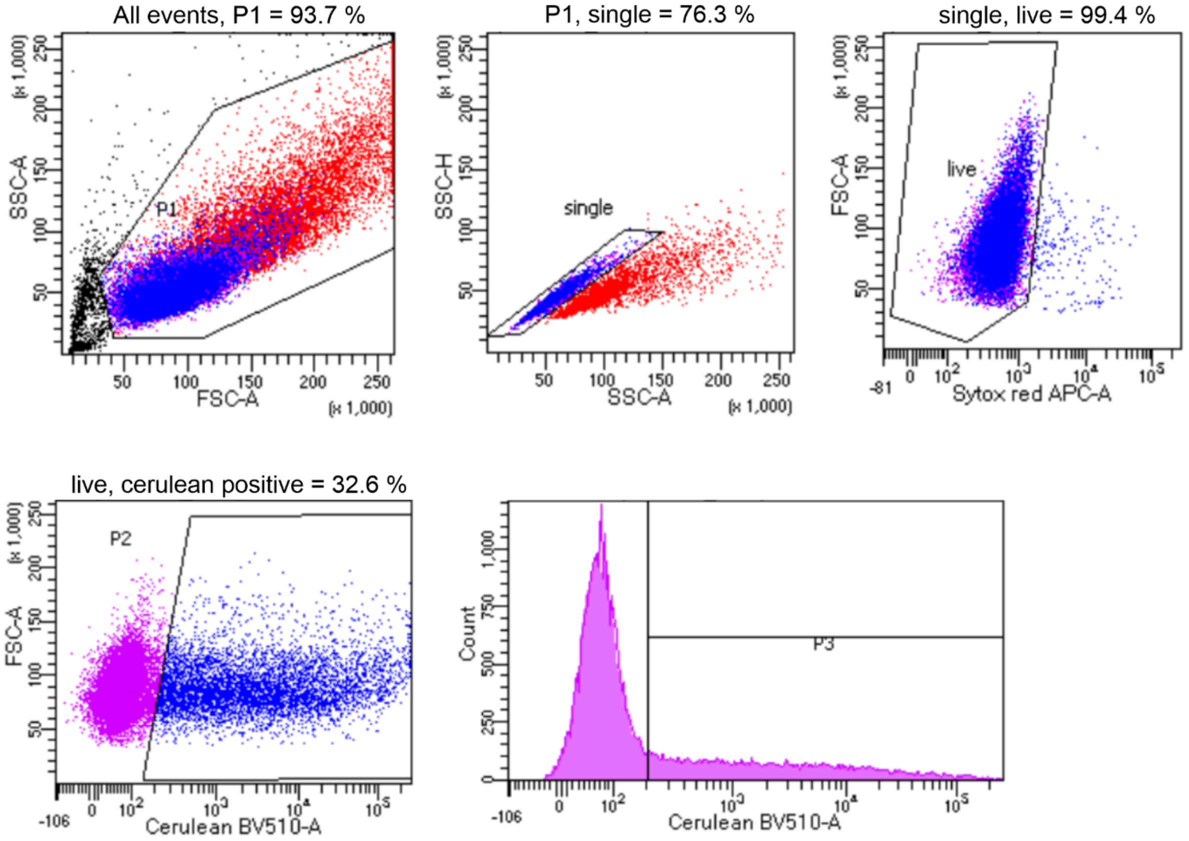

Fig. S9 Gating Strategy used for flow cytometry on HEK293 cells transfected with split cerulean constructs.

Table S1. Binding domain analysis.

Binding domain	Reconstitution efficiency WB ± SEM [%]	Reconstitution efficiency FC ± SEM [%]	Length [bp]	GC content [%]
1	2.1 ± 2.1	1.7 ± 0.9	1227	55.3
2	27.0 ± 5.8	17.3 ± 1.2	267	55.1
3	5.7 ± 1.8	4.2 ± 0.4	100	57.0
4	37.3 ± 5.4	31.5 ± 1.7	51	56.9
5	39.9 ± 2.3	36.4 ± 1.4	102	62.7
6	24.2 ± 6.6	12.1 ± 0.2	72	56.9
7	16.7 ± 2.1	13.6 ± 0.5	438	56.8
8	32.9 ± 2.5	35.8 ± 1.8	105	59.0
9	29.2 ± 9.4	24.0 ± 0.7	100	47.0
10	53.5 ± 5.7	44.0 ± 1.3	100	46.0
11	30.7 ± 5.0	20.3 ± 0.7	100	48.0
12	13.2 ± 5.0	16.8 ± 0.5	100	48.0

Table S2. Trans-splicing elements used for REVeRT.

Element	Sequence
BD: BD10	CATCTGACCACC**T**GCGAA(Δ5bp)TTTTTGCATC**CT**GCTG**TT**TAAT **CT**GCGTTGG(Δ3bp)TTTAACCGCC**T**GTC**T**GGCTTT**T**TTTCAC**T**GA TGTG(Δ1bp)AT*CGCCTTGATGCACT*
SAS: SAS1	CAACGAGTCTTTTGTCATCTACAG|G
SDS: SDS1	AG|GTAAG

BD domain contains following modifications compared to the native lacZ sequence: 11x substitution (bold), 3x deletion (Δ), 1x artificial sequence (*italic*).

Table S3. Differentially expressed genes.

Gene	ID	log2FoldChange	pvalue	padj
Rad54b	ENSMUSG00000078773	2.36	1.72E-16	3.79E-13
Manba	ENSMUSG00000028164	1.77	8.14E-38	1.08E-33
Slc6a2	ENSMUSG00000055368	1.65	1.88E-29	1.24E-25
Gm19410	ENSMUSG00000109372	1.59	4.31E-10	5.18E-07
Frrs1	ENSMUSG00000033386	1.46	5.76E-13	1.09E-09
Podn	ENSMUSG00000028600	1.39	6.49E-12	1.07E-08
Opn1mw	ENSMUSG00000031394	1.29	1.87E-25	8.22E-22
Ifi47	ENSMUSG00000078920	1.20	1.09E-06	5.61E-04
Gm16062	ENSMUSG00000087249	1.18	6.95E-07	4.48E-04
Iigp1	ENSMUSG00000054072	1.13	1.91E-06	7.88E-04
BC065403	ENSMUSG00000097211	1.13	1.40E-04	1.01E-02
Cutal	ENSMUSG00000026870	1.09	6.47E-06	1.68E-03
1500026H17Rik	ENSMUSG00000097383	1.09	9.25E-05	7.69E-03
Fam204a	ENSMUSG00000057858	1.09	6.62E-17	1.75E-13
Shisa2	ENSMUSG00000044461	1.07	4.18E-18	1.38E-14
Gucy1a2	ENSMUSG00000041624	-1.07	1.00E-03	2.95E-02
Malat1	ENSMUSG00000092341	-1.10	1.77E-03	3.98E-02
Klf12	ENSMUSG00000072294	-1.17	2.02E-06	7.97E-04
Kcnh5	ENSMUSG00000034402	-1.18	3.85E-04	1.72E-02
Dok6	ENSMUSG00000073514	-1.28	5.92E-05	5.79E-03
Gm11808	ENSMUSG00000068240	-1.28	7.70E-04	2.51E-02
Gm340	ENSMUSG00000090673	-1.55	6.85E-06	1.68E-03
Rho	ENSMUSG00000030324	-1.69	6.85E-06	1.68E-03
Cdr1	ENSMUSG00000090546	-2.22	4.37E-04	1.84E-02
Gm22009	ENSMUSG00000089417	-2.29	1.11E-04	8.76E-03
Gm26917	ENSMUSG00000097971	-2.81	3.22E-04	1.58E-02
Scarna2	ENSMUSG00000088185	-3.37	6.60E-05	6.23E-03
Hist1h4d	ENSMUSG00000061482	-3.59	5.06E-04	2.00E-02
Rmrp	ENSMUSG00000088088	-3.68	1.87E-03	4.05E-02
Gm23935	ENSMUSG00000076258	-3.71	3.06E-04	1.51E-02
Lars2	ENSMUSG00000035202	-3.72	2.85E-04	1.45E-02
Rn7sk	ENSMUSG00000065037	-3.94	4.07E-04	1.76E-02
Gm24187	ENSMUSG00000088609	-3.96	1.35E-04	1.00E-02
Gm42418	ENSMUSG00000098178	-4.42	3.62E-09	3.42E-06
Gm24270	ENSMUSG00000076281	-4.55	4.02E-05	4.62E-03
Mir6236	ENSMUSG00000098973	-5.32	1.12E-05	2.13E-03
Gm15564	ENSMUSG00000086324	-5.37	6.06E-06	1.61E-03

Significant up- or downregulation is defined as absolute log2 fold change > 1 and adjusted p-value < 0.05. DESeq2 was used for RNA-Seq analysis. This includes using the two-sided Wald test to obtain log2 and p-values and using the Benjamini-Hochberg procedure as a correction for multiple testing to obtain adjusted p-values.

Table S4 Splicing events with endogenous transcripts in Cas9-VPR injected retinas.

5‘ Gene	3‘ Gene	# of reads	Eye injected with
5‘ Cas9	Rho exon 3	22	Cas9-VPR + g1-g4
5‘ Cas9	Opn1mw exon 2	20	Cas9-VPR + g1-g4
5‘ Cas9	Rho g1 cut site	11	Cas9-VPR + g1-g4
5‘ Cas9	Pacsin2 exon 2	4	Cas9-VPR + g1-g4
5‘ Cas9	Stam2 exon 14	3	Cas9-VPR + g1-g4
5‘ Cas9	Rtn4rl2 exon 3	2	Cas9-VPR + g1-g4
5‘ Cas9	Mecp2 exon 2	2	Cas9-VPR + g1-g4
5‘ Cas9	Paip2 exon 3	2	Cas9-VPR + g1-g4
5‘ Cas9	Gmps exon 13	2	Cas9-VPR + g1-g4
Cpsf6 exon 9	3‘ Cas9	2	Cas9-VPR + g1-g4
5‘ Cas9	Slc38a6 exon 13	1	Cas9-VPR + g1-g4
5‘ Cas9	Slc30a9 exon 2	1	Cas9-VPR + g1-g4
5‘ Cas9	Lpcat1 exon 2	1	Cas9-VPR + g1-g4
5‘ Cas9	Sdf2 exon 4	1	Cas9-VPR + g1-g4
5‘ Cas9	Cfap418 exon 2	1	Cas9-VPR + g1-g4
5‘ Cas9	Abdhd14a exon 3	1	Cas9-VPR + g1-g4
5‘ Cas9	Tbc1d8 exon 2	1	Cas9-VPR + g1-g4
5‘ Cas9	Pard3 exon 23	1	Cas9-VPR + g1-g4
5‘ Cas9	Zmat1 exon 2	1	Cas9-VPR + g1-g4
5‘ Cas9	Opn1mw exon 1	1	Cas9-VPR + g1-g4
5‘ Cas9	AW046200 lncRNA	1	Cas9-VPR + g1-g4
Arid3b exon 3	3‘ Cas9	1	Cas9-VPR + g1-g4
5‘ Cas9	Garnl3 exon 10	2	Cas9-VPR + gL
Pacsin2 exon 1	3‘ Cas9	2	Cas9-VPR + gL
5‘ Cas9	Rho exon 3	1	Cas9-VPR + gL
5‘ Cas9	Stam2 exon 14	1	Cas9-VPR + gL
5‘ Cas9	Aatf exon 6	1	Cas9-VPR + gL
5‘ Cas9	Cox7b exon 2	1	Cas9-VPR + gL
5‘ Cas9	Shld1 exon 3	1	Cas9-VPR + gL
5‘ Cas9	Ulk4 exon 33	1	Cas9-VPR + gL
5‘ Cas9	Srgap2 exon 6	1	Cas9-VPR + gL
5‘ Cas9	Atp1a3 exon 17	1	Cas9-VPR + gL
5‘ Cas9	Rims2 exon 23	1	Cas9-VPR + gL
5‘ Cas9	Gls exon 4	1	Cas9-VPR + gL
5‘ Cas9	Gt(ROSA)26Sor	1	Cas9-VPR + gL

Table S5 sgRNA sequences.

sgRNA	Spacer sequence	Target and purpose	Figure
g1 Myo7b	AGACTCCAAGAACGCCAGTC	Transcriptional activation of *Myo7b*	3, S4
g2 Myo7b	GGGCACCATTAACCACTGCT	Transcriptional activation of *Myo7b*	3, S4
g3 Myo7b	GGAAGGGCTCCAAGCGGAAC	Transcriptional activation of *Myo7b*	3, S4
g1 Rho	GTACGGTGACGTAGAGCGTG	*Rho* exon 1, Knockout of *Rho*	4, S5
g2 Opn1mw	GGGGCCTTTAAGGTA	Transcriptional activation of *Opn1mw*	4, S5
g3 Opn1mw	GCCACCCCTGTGGAT	Transcriptional activation of *Opn1mw*	4, S5
g4 Opn1mw	CTTGCTTGTTTACAA	Transcriptional activation of *Opn1mw*	4, S5
g5 Opn1mw	GTCCTGTAACCCCAT	Transcriptional activation of *Opn1mw*	S5
g6 Opn1mw	GATGATCTAAGTCCT	Transcriptional activation of *Opn1mw*	S5
g7 Opn1mw	CTGCAGGATCAGCCC	Transcriptional activation of *Opn1mw*	S5
Ush2a exon12 #2	CAGACCTCACAAGCACTCCA	*Ush2a* exon 12, Nicking gRNA for PE3	S5

Table S6 pegRNA sequences used in Fig. S5.

pegRNA	Spacer sequence	3‘ extension	RT template length (nt)	PBS length (nt)
*HEK3*_CTT	GGCCCAGACTGA GCACGTGA	TCTGCCATCAAAGC GTGCTCAGTCTG	13	13
*Dnmt1*_5d_5 GtoT	CGGGCTGGAGC TGTTCGCGC	AAGATGCAAGCGC GAACAGCTCCAG	12	13
Ush2a exon 12 #1 + #2	AACTCTGTGATC CGCTTTCT	TGACACTGCCCAGA AAGCGGATCACAGA	15	13
Rho exon 1 #1	AGTACTGCGGCT GCTCGAAG	GGAGTCCCTTCGAG CAGCCGCAG	10	13
Rho exon 1 #2	CCAACGTCACAG GCGTGGTG	AGGGGCTTCGCAC CACGCCTGTGACG	13	13
Rho exon 1 #3	AGTACTGCGGCT GCTCGAAG	GTGCGGAGTCCCTT CGAGCAGCCGCA	14	12
Cnga1 exon 9 #1	CAAGAAAGGGGA CATCGGGC	GTACATCTCTCGCC CGATGTCCCCTTT	14	13
Cnga1 exon 9 #2	TATGCAAGAAAG GGGACATC	TACATCTCCCGTCC GATGTCCCCTTTC	17	10
Cnga1 exon 9 #3	CAAGAAAGGGGA CATCGGGC	GTACATCTCTCGCC CGATGTCCCCTT	14	12

Table S7 Primer sequences.

Primer	Sequence (5‘ – 3‘)
Cas9 forward	AGAACGCTTGAAAACTTACGCT
Cas9 reverse	TTGATGTCCAGTTCCTGATCC
Cerulean forward	ATGGTGAGCAAGGGCGAGG
Cerulean reverse	CTTGTACAGCTCGTCCATGCC
Cnga1 forward	TGTGAAGCTGGTCTGTTGGT
Cnga1 reverse	CCTCCCTTTCTCTTCCAGCA
Dnmt1 forward	GTGTGGTACATGCTGCTTCCG
Dnmt reverse	CTCCCTCAAGCTCCCAGTCAAT
HEK3 forward	GCATGCATTTGTAGGCTTGA
HEK3 reverse	CTTTTCCTCTGTTGAGCTCG
HEK3 + adapter forward	ACACTCTTTCCCTACACGACGCTCTTCCGATCTGC ATGCATTTGTAGGCTTGA
HEK3 + adapter reverse	GACTGGAGTTCAGACGTGTGCTCTTCCGATCTCT TTTCCTCTGTTGAGCTCG
ITR2 forward	GGAACCCCTAGTGATGGAGTT
ITR2 reverse	CGGCCTCAGTGAGCGA
PE2 forward	CGATGTGGACGCTATCGTGC
PE2 reverse	GCCTTGCCGATTTCCTGCTC
qRT-PCR 5’ABCA4 F	ACCACCACCTTGTCCATCCT
qRT-PCR 5’ABCA4 R	ATGTGCTCAGCCACCGTGAG
qRT-PCR 3’ABCA4 F	GTCCCCAGCACAGCCTATGT
qRT-PCR 3’ABCA4 R	CTGAGCAGCGTCCGGTTATT
qRT-PCR 5‘+3‘ ABCA4 F (split-site spanning)	CTGCTCCTGAAGTATCGCTCAG
qRT-PCR 5‘+3‘ ABCA4 R (split-site spanning)	AGCCTGTGCCAAAGCAGTTC
qRT-PCR Alas forward	TCGCCGATGCCCATTCTTATC
qRT-PCR Alas reverse	GGCCCCAACTTCCATCATCT
qRT-PCR Actb forward	CACACCCGCCACCAGTTC
qRT-PCR Actb reverse	CCCACGATGGAGGGGAATAC
qRT-PCR Myo7b forward	GGGACACAAGTACAGGAAGGA
qRT-PCR Myo7b reverse	GCGTTCAAAGCCCACTAGG
qRT-PCR Cas9 reconst forward	AGCACAAGTTTCTGGCCAGGG
qRT-PCR Cas9 reconst reverse	GGTAGTTTGGTTCTCTCGGGCC
qRT-PCR Opn1mw forward	GGAGCAGGTACTGGCCTTATG
qRT-PCR Opn1mw reverse	GGAGGTAGCAGAGCACGATG
qRT-PCR Rho forward	GGATCATGGCGTTGGCCTGT
qRT-PCR Rho reverse	CCGCATGAACATTGCATGCCC
Rho forward	AGCCTTGGTCTCTGTCTACG
Rho reverse	TGGTGAATCCTCCGAAGACC
Ush2a forward	CCAGGGCTTAGATGCAATCG
Ush2a reverse	AAGTAGCCTGCCTTACACTG

Supplementary Note 1: Sequences of REVeRT plasmids for therapeutic purposes.

Sequences provided:

1. pAAV-3xsgMyo7b-CMV-5'dCas9-SDS-BD10-SV40pA (7484 bp)
2. pAAV-CMV-BD10-SAS620-3'dCas9-VPR-synpA (7699 bp)
3. pAAV-sglacZ-RHO-5'Cas9-SDS-BD10-SV40pA (6351 bp)
4. pAAV-RHO-SAS-BD10-3'Cas9-VPR-synpA (7285 bp)
5. pAAV-sgRho-2xsgOpn1mw-RHO-5'Cas9-SDS-BD10-SV40pA (7056 bp)
6. pAAV-sgRho-3xsgOpn1mw-RHO-5'Cas9-SDS-BD10-SV40pA (7413 bp)
7. pAAV-sgRho-2xsgOpn1mw-RHO-Cas9N-RmaIntN-SV40pA (7154 bp)
8. pAAV-RHO-RmaIntC-Cas9C-VPR-synpA (7343 bp)
9. pGL2-RHO-5'ABCA4-SDS-BD10-SV40pA (9742 bp)
10. pGL2-RHO-BD10-SAS-3'ABCA4-SV40pA (9858 bp)
11. pGL2-GRK1-5'ABCA4-BD10-SDS-SV40pA (9929 bp)
12. pGL2-GRK1-BD10-SAS-3'ABCA4-SV40pA (10045 bp)

Color code:

Gene of interest coding sequence

Intronic part of SAS

Intronic part of SDS

SV40pA

synpA

BD10

N- or C-intein

RHO promoter

CMV enhancer + promoter

hU6 promoter

GRK1 promoter

1. **pAAV-3xsgMyo7b-CMV-5’dCas9-SDS-BD10-SV40pA (7484 bp)**

CAGGAAACAGCTATGACCATGATTACGCCAGATTTAATTAAGGCTGCGCGCTCGCTCGCTCACTG
AGGCCGCCCGGGCAAAGCCCGGGCGTCGGGCGACCTTTGGTCGCCCGGCCTCAGTGAGCGAG
CGAGCGCGCAGAGAGGGAGTGGCCAACTCCATCACTAGGGGTTCCTTGTAGTTAATGATTAACC
CGCCATGCTACTTATCTACGTAGCCATGCTCTAGGAAGATCGGAATTCGCCCTTAAGGGCGCGC
CGTTTAAACGAGGGCCTATTTCCCATGATTCCTTCATATTTGCATATACGATACAAGGCTGTTAGA
GAGATAATTGGAATTAATTTGACTGTAAACACAAAGATATTAGTACAAAATACGTGACGTAGAAAG
TAATAATTTCTTGGGTAGTTTGCAGTTTTAAAATTATGTTTTAAAATGGACTATCATATGCTTACCGT
AACTTGAAAGTATTTCGATTTCTTGGCTTTATATATCTTGTGGAAAGGACGAAACACCGAGACTCC
AAGAACGCCAGTCGTTTTAGAGCTAGAAATAGCAAGTTAAAATAAGGCTAGTCCGTTATCAACTTG
AAAAAGTGGCACCGAGTCGGTGCTTTTTTTGTATACGAGGGCCTATTTCCCATGATTCCTTCATAT
TTGCATATACGATACAAGGCTGTTAGAGAGATAATTGGAATTAATTTGACTGTAAACACAAAGATA
TTAGTACAAAATACGTGACGTAGAAAGTAATAATTTCTTGGGTAGTTTGCAGTTTTAAAATTATGTT
TTAAAATGGACTATCATATGCTTACCGTAACTTGAAAGTATTTCGATTTCTTGGCTTTATATATCTT
GTGGAAAGGACGAAACACCGGGGCACCATTAACCACTGCTGTTTTAGAGCTAGAAATAGCAAGTT
AAAATAAGGCTAGTCCGTTATCAACTTGAAAAAGTGGCACCGAGTCGGTGCTTTTTTTGTTAACGA
GGGCCTATTTCCCATGATTCCTTCATATTTGCATATACGATACAAGGCTGTTAGAGAGATAATTGG
AATTAATTTGACTGTAAACACAAAGATATTAGTACAAAATACGTGACGTAGAAAGTAATAATTTCTT
GGGTAGTTTGCAGTTTTAAAATTATGTTTTAAAATGGACTATCATATGCTTACCGTAACTTGAAAGT
ATTTCGATTTCTTGGCTTTATATATCTTGTGGAAAGGACGAAACACCGGGAAGGGCTCCAAGCGG
AACGTTTTAGAGCTAGAAATAGCAAGTTAAAATAAGGCTAGTCCGTTATCAACTTGAAAAAGTGGC
ACCGAGTCGGTGCTTTTTTTATTTAAATAGCTAGCACGCGTTGACATTGATTATTGACTAGTTATTA
ATAGTAATCAATTACGGGGTCATTAGTTCATAGCCCATATATGGAGTTCCGCGTTACATAACTTAC
GGTAAATGGCCCGCCTGGCTGACCGCCCAACGACCCCCGCCCATTGACGTCAATAATGACGTAT
GTTCCCATAGTAACGCCAATAGGGACTTTCCATTGACGTCAATGGGTGGAGTATTTACGGTAAAC
TGCCCACTTGGCAGTACATCAAGTGTATCATATGCCAAGTACGCCCCCTATTGACGTCAATGACG
GTAAATGGCCCGCCTGGCATTATGCCCAGTACATGACCTTATGGGACTTTCCTACTTGGCAGTAC
ATCTACGTATTAGTCATCGCTATTACCATGGTGATGCGGTTTTGGCAGTACATCAATGGGCGTGG
ATAGCGGTTTGACTCACGGGGATTTCCAAGTCTCCACCCCATTGACGTCAATGGGAGTTTGTTTT
GGCACCAAAATCAACGGGACTTTCCAAAATGTCGTAACAACTCCGCCCCATTGACGCAAATGGGC
GGTAGGCGTGTACGGTGGGAGGTCTATATAAGCAGAGCTCGTTTAGTGAACCGTCAGAACCGGT
CGACTAGAGGATCCATGGCCCCAAAGAAGAAGCGGAAGGTCGGTATCCACGGAGTCCCAGCAG
CCGACAAGAAGTACTCCATTGGGCTCGCTATCGGCACAAACAGCGTCGGCTGGGCCGTCATTAC
GGACGAGTACAAGGTGCCGAGCAAAAAATTCAAAGTTCTGGGCAATACCGATCGCCACAGCATA
AAGAAGAACCTCATTGGCGCCCTCCTGTTCGACTCCGGGGAAACGGCCGAAGCCACGCGGCTC
AAAAGAACAGCACGGCGCAGATATACCCGCAGAAAGAATCGGATCTGCTACCTGCAGGAGATCT
TTAGTAATGAGATGGCTAAGGTGGATGACTCTTTCTTCCATAGGCTGGAGGAGTCCTTTTTGGTG
GAGGAGGATAAAAAGCACGAGCGCCACCCAATCTTTGGCAATATCGTGGACGAGGTGGCGTACC
ATGAAAAGTACCCAACCATATATCATCTGAGGAAGAAGCTTGTAGACAGTACTGATAAGGCTGAC
TTGCGGTTGATCTATCTCGCGCTGGCGCATATGATCAAATTTCGGGGACACTTCCTCATCGAGGG
GGACCTGAACCCAGACAACAGCGATGTCGACAAACTCTTTATCCAACTGGTTCAGACTTACAATC
AGCTTTTCGAAGAGAACCCGATCAACGCATCCGGAGTTGACGCCAAAGCAATCCTGAGCGCTAG
GCTGTCCAAATCCCGGCGGCTCGAAAACCTCATCGCACAGCTCCCTGGGGAGAAGAAGAACGG
CCTGTTTGGTAATCTTATCGCCCTGTCACTCGGGCTGACCCCCAACTTTAAATCTAACTTCGACCT
GGCCGAAGATGCCAAGCTTCAACTGAGCAAAGACACCTACGATGATGATCTCGACAATCTGCTG
GCCCAGATCGGCGACCAGTACGCAGACCTTTTTTTGGCGGCAAAGAACCTGTCAGACGCCATTC
TGCTGAGTGATATTCTGCGAGTGAACACGGAGATCACCAAAGCTCCGCTGAGCGCTAGTATGATC
AAGCGCTATGATGAGCACCACCAAGACTTGACTTTGCTGAAGGCCCTTGTCAGACAGCAACTGCC
TGAGAAGTACAAGGAAATTTTCTTCGATCAGTCTAAAAATGGCTACGCCGGATACATTGACGGCG
GAGCAAGCCAGGAGGAATTTTACAAATTTATTAAGCCCATCTTGGAAAAAATGGACGGCACCGAG
GAGCTGCTGGTAAAGCTTAACAGAGAAGATCTGTTGCGCAAACAGCGCACTTTCGACAATGGAAG
CATCCCCCACCAGATTCACCTGGGCGAACTGCACGCTATCCTCAGGCGGCAAGAGGATTTCTAC
CCCTTTTTGAAAGATAACAGGGAAAAGATTGAGAAAATCCTCACATTTCGGATACCCTACTATGTA
GGCCCCCTCGCCCGGGGAAATTCCAGATTCGCGTGGATGACTCGCAAATCAGAAGAGACCATCA
CTCCCTGGAACTTCGAGGAAGTCGTGGATAAGGGGGCCTCTGCCCAGTCCTTCATCGAAAGGAT
GACTAACTTTGATAAAAATCTGCCTAACGAAAAGGTGCTTCCTAAACACTCTCTGCTGTACGAGTA
CTTCACAGTTTATAACGAGCTCACCAAGGTCAAATACGTCACAGAAGGGATGAGAAAGCCAGCAT

TCCTGTCTGGAGAGCAGAAGAAAGCTATCGTGGACCTCCTCTTCAAGACGAACCGGAAAGTTACC
GTGAAACAGCTCAAAGAAGACTATTTCAAAAAGATTGAATGTTTCGACTCTGTTGAAATCAGCGGA
GTGGAGGATCGCTTCAACGCATCCCTGGGAACGTATCACGATCTCCTGAAAATCATTAAAGACAA
GGACTTCCTGGACAATGAGGAGAACGAGGACATTCTTGAGGACATTGTCCTCACCCTTACGTTGT
TTGAAGATAGGGAGATGATTGAAGAACGCTTGAAAACTTACGCTCATCTCTTCGACGACAAAGTC
ATGAAACAGCTCAAGAGGCGCCGATATACAGGATGGGGGCGGCTGTCAAGAAAACTGATCAATG
GGATCCGAGACAAGCAGAGTGGAAAGACAATCCTGGATTTTCTTAAGTCCGATGGATTTGCCAAC
CGGAACTTCATGCAGTTGATCCATGATGACTCTCTCACCTTTAAGGAGGACATCCAGAAAGCACA
AGTTTCTGGCCAGGGGGACAGTCTTCACGAGCACATCGCTAATCTTGCAGGTAAGGGCACTGAG
CAGAAGGGAAGAAGCTCCGGGGGCTCTTTGTAGGGTGCGGCCGCAGTGCATCAAGGCGATCAC
ATCAGTGAAAAAAAGCCAGACAGGCGGTTAAACCAACGCAGATTAAACAGCAGGATGCAAAAATT
CGCAGGTGGTCAGATGGCGGCCGCTCTAGACTCGATGAGTTTGGACAAACCACAACTAGAATGC
AGTGAAAAAAATGCTTTATTTGTGAAATTTGTGATGCTATTGCTTTATTTGTAACCATTATAAGCTG
CAATAAACAAGTTCTCGAGTTAAGGGCGAATTCCCGATTAGGATCTTCCTAGAGCATGGCTACGT
AGATAAGTAGCATGGCGGGTTAATCATTAACTACAAGGAACCCCTAGTGATGGAGTTGGCCACTC
CCTCTCTGCGCGCTCGCTCGCTCACTGAGGCCGGGCGACCAAAGGTCGCCCGACGCCCGGGCT
TTGCCCGGGCGGCCTCAGTGAGCGAGCGAGCGCGCAGCCTTAATTAACCTAATTCACTGGCCGT
CGTTTTACAACGTCGTGACTGGGAAAACCCTGGCGTTACCCAACTTAATCGCCTTGCAGCACATC
CCCCTTTCGCCAGCTGGCGTAATAGCGAAGAGGCCCGCACCGATCGCCCTTCCCAACAGTTGCG
CAGCCTGAATGGCGAATGGGACGCGCCCTGTAGCGGCGCATTAAGCGCGGCGGGTGTGGTGGT
TACGCGCAGCGTGACCGCTACACTTGCCAGCGCCCTAGCGCCCGCTCCTTTCGCTTTCTTCCCT
TCCTTTCTCGCCACGTTCGCCGGCTTTCCCCGTCAAGCTCTAAATCGGGGGCTCCCTTTAGGGTT
CCGATTTAGTGCTTTACGGCACCTCGACCCCAAAAAACTTGATTAGGGTGATGGTTCACGTAGTG
GGCCATCGCCCCGATAGACGGTTTTTCGCCCTTTGACGCTGGAGTTCACGTTCCTCAATAGTGGA
CTCTTGTTCCAAACTGGAACAACACTCAACCCTATCTCGGTCTATTCTTTTGATTTATAAGGGATTT
TTCCGATTTCGGCCTATTGGTTAAAAAATGAGCTGATTTAACAAAAATTTAACGCGAATTTTAACAA
AATATTAACGTTTATAATTTCAGGTGGCATCTTTCGGGGAAATGTGCGCGGAACCCCTATTTGTTT
ATTTTTCTAAATACATTCAAATATGTATCCGCTCATGAGACAATAACCCTGATAAATGCTTCAATAA
TATTGAAAAAGGAAGAGTATGAGTATTCAACATTTCCGTGTCGCCCTTATTCCCTTTTTTGCGGCA
TTTTGCCTTCCTGTTTTTGCTCACCCAGAAACGCTGGTGAAAGTAAAAGATGCTGAAGATCAGTTG
GGTGCACGAGTGGGTTACATCGAACTGGATCTCAATAGTGGTAAGATCCTTGAGAGTTTTCGCCC
CGAAGAACGTTTTCCAATGATGAGCACTTTTAAAGTTCTGCTATGTGGCGCGGTATTATCCCGTAT
TGACGCCGGGCAAGAGCAACTCGGTCGCCGCATACACTATTCTCAGAATGACTTGGTTGAGTACT
CACCAGTCACAGAAAAGCATCTTACGGATGGCATGACAGTAAGAGAATTATGCAGTGCTGCCATA
ACCATGAGTGATAACACTGCGGCCAACTTACTTCTGACAACGATCGGAGGACCGAAGGAGCTAA
CCGCTTTTTTGCACAACATGGGGGATCATGTAACTCGCCTTGATCGTTGGGAACCGGAGCTGAAT
GAAGCCATACCAAACGACGAGCGTGACACCACGATGCCTGTAGTAATGGTAACAACGTTGCGCA
AACTATTAACTGGCGAACTACTTACTCTAGCTTCCCGGCAACAATTAATAGACTGGATGGAGGCG
GATAAAGTTGCAGGACCACTTCTGCGCTCGGCCCTTCCGGCTGGCTGGTTTATTGCTGATAAATC
TGGAGCCGGTGAGCGTGGGTCTCGCGGTATCATTGCAGCACTGGGGCCAGATGGTAAGCCCTC
CCGTATCGTAGTTATCTACACGACGGGGAGTCAGGCAACTATGGATGAACGAAATAGACAGATCG
CTGAGATAGGTGCCTCACTGATTAAGCATTGGTAACTGTCAGACCAAGTTTACTCATATATACTTT
AGATTGATTTAAAACTTCATTTTTAATTTAAAAGGATCTAGGTGAAGATCCTTTTTGATAATCTCATG
ACCAAAATCCCTTAACGTGAGTTTTCGTTCCACTGAGCGTCAGACCCCGTAGAAAAGATCAAAGG
ATCTTCTTGAGATCCTTTTTTTCTGCGCGTAATCTGCTGCTTGCAAACAAAAAAACCACCGCTACC
AGCGGTGGTTTGTTTGCCGGATCAAGAGCTACCAACTCTTTTTCCGAAGGTAACTGGCTTCAGCA
GAGCGCAGATACCAAATACTGTCCTTCTAGTGTAGCCGTAGTTAGGCCACCACTTCAAGAACTCT
GTAGCACCGCCTACATACCTCGCTCTGCTAATCCTGTTACCAGTGGCTGCTGCCAGTGGCGATAA
GTCGTGTCTTACCGGGTTGGACTCAAGACGATAGTTACCGGATAAGGCGCAGCGGTCGGGCTGA
ACGGGGGGTTCGTGCACACAGCCCAGCTTGGAGCGAACGACCTACACCGAACTGAGATACCTAC
AGCGTGAGCTATGAGAAAGCGCCACGCTTCCCGAAGGGAGAAAGGCGGACAGGTATCCGGTAA
GCGGCAGGGTCGGAACAGGAGAGCGCACGAGGGAGCTTCCAGGGGGAAACGCCTGGTATCTTT
ATAGTCCTGTCGGGTTTCGCCACCTCTGACTTGAGCGTCGATTTTTGTGATGCTCGTCAGGGGGG
CGGAGCCTATGGAAAAACGCCAGCAACGCGGCCTTTTTACGGTTCCTGGCCTTTTGCTGCGGTTT
TGCTCACATGTTCTTTCCTGCGTTATCCCCTGATTCTGTGGATAACCGTATTACCGCCTTTGAGTG
AGCTGATACCGCTCGCCGCAGCCGAACGACCGAGCGCAGCGAGTCAGTGAGCGAGGAAGCGGA
AGAGCGCCCAATACGCAAACCGCCTCTCCCCGCGCGTTGGCCGATTCATTAATGCAGCTGGCAC

GACAGGTTTCCCGACTGGAAAGCGGGCAGTGAGCGCAACGCAATTAATGTGAGTTAGCTCACTC
ATTAGGCACCCCAGGCTTTACACTTTATGCTTCCGGCTCGTATGTTGTGTGGAATTGTGAGCGGA
TAACAATTTCACA

2. pAAV-CMV-BD10-SAS-3'dCas9-VPR-synpA (7699 bp)

CAGGAAACAGCTATGACCATGATTACGCCAGATTTAATTAAGGCTGCGCGCTCGCTCGCTCACTG
AGGCCGCCCGGGCAAAGCCCGGGCGTCGGGCGACCTTTGGTCGCCCGGCCTCAGTGAGCGAG
CGAGCGCGCAGAGAGGGAGTGGCCAACTCCATCACTAGGGGTTCCTTGTAGTTAATGATTAACC
CGCCATGCTACTTATCTACGTAGCCATGCTCTAGGAAGATCGGAATTCGCCCTTAAGGGCGCGC
CGTTTAAATAGCTAGCACGCGTTGACATTGATTATTGACTAGTTATTAATAGTAATCAATTACGGG
GTCATTAGTTCATAGCCCATATATGGAGTTCCGCGTTACATAACTTACGGTAAATGGCCCGCCTG
GCTGACCGCCCAACGACCCCCGCCCATTGACGTCAATAATGACGTATGTTCCCATAGTAACGCCA
ATAGGGACTTTCCATTGACGTCAATGGGTGGAGTATTTACGGTAAACTGCCCACTTGGCAGTACA
TCAAGTGTATCATATGCCAAGTACGCCCCCTATTGACGTCAATGACGGTAAATGGCCCGCCTGGC
ATTATGCCCAGTACATGACCTTATGGGACTTTCCTACTTGGCAGTACATCTACGTATTAGTCATCG
CTATTACCATGGTGATGCGGTTTTGGCAGTACATCAATGGGCGTGGATAGCGGTTTGACTCACGG
GGATTTCCAAGTCTCCACCCCATTGACGTCAATGGGAGTTTGTTTTGGCACCAAAATCAACGGGA
CTTTCCAAAATGTCGTAACAACTCCGCCCCATTGACGCAAATGGGCGGTAGGCGTGTACGGTGG
GAGGTCTATATAAGCAGAGCTCGTTTAGTGAACCGTCAGAGGTACCACCGGTCGACTAGAGGAT
CCAGGTGCGGCCGCCATCTGACCACCTGCGAATTTTTGCATCCTGCTGTTTAATCTGCGTTGGTT
TAACCGCCTGTCTGGCTTTTTTTCACTGATGTGATCGCCTTGATGCACTGCGGCCGCCAACGAGT
CTTTTGTCATCTACAGGTAGCCCAGCTATCAAAAAGGGAATACTGCAGACCGTTAAGGTCGTGGA
TGAACTCGTCAAAGTAATGGGAAGGCATAAGCCCGAGAATATCGTTATCGAGATGGCCCGAGAG
AACCAAACTACCCAGAAGGGACAGAAGAACAGTAGGGAAAGGATGAAGAGGATTGAAGAGGGTA
TAAAAGAACTGGGGTCCCAAATCCTTAAGGAACACCCAGTTGAAAACACCCAGCTTCAGAATGAG
AAGCTCTACCTGTACTACCTGCAGAACGGCAGGGACATGTACGTGGATCAGGAACTGGACATCA
ATCGGCTCTCCGACTACGACGTGGATGCTATCGTGCCCCAGTCTTTTCTCAAAGATGATTCTATT
GATAATAAAGTGTTGACAAGATCCGATAAAAATAGAGGGAAGAGTGATAACGTCCCCTCAGAAGA
AGTTGTCAAGAAAATGAAAAATTATTGGCGGCAGCTGCTGAACGCCAAACTGATCACACAACGGA
AGTTCGATAATCTGACTAAGGCTGAACGAGGTGGCCTGTCTGAGTTGGATAAAGCCGGCTTCATC
AAAAGGCAGCTTGTTGAGACACGCCAGATCACCAAGCACGTGGCCCAAATTCTCGATTCACGCAT
GAACACCAAGTACGATGAAAATGACAAACTGATTCGAGAGGTGAAAGTTATTACTCTGAAGTCTAA
GCTGGTCTCAGATTTCAGAAAGGACTTTCAGTTTTATAAGGTGAGAGAGATCAACAATTACCACCA
TGCGCATGATGCCTACCTGAATGCAGTGGTAGGCACTGCACTTATCAAAAAATATCCCAAGCTTG
AATCTGAATTTGTTTACGGAGACTATAAAGTGTACGATGTTAGGAAAATGATCGCAAAGTCTGAGC
AGGAAATAGGCAAGGCCACCGCTAAGTACTTCTTTTACAGCAATATTATGAATTTTTTCAAGACCG
AGATTACACTGGCCAATGGAGAGATTCGGAAGCGACCACTTATCGAAACAAACGGAGAAACAGG
AGAAATCGTGTGGGACAAGGGTAGGGATTTCGCGACAGTCCGGAAGGTCCTGTCCATGCCGCAG
GTGAACATCGTTAAAAAGACCGAAGTACAGACCGGAGGCTTCTCCAAGGAAAGTATCCTCCCGAA
AAGGAACAGCGACAAGCTGATCGCACGCAAAAAAGATTGGGACCCCAAGAAATACGGCGGATTC
GATTCTCCTACAGTCGCTTACAGTGTACTGGTTGTGGCCAAAGTGGAGAAAGGGAAGTCTAAAAA
ACTCAAAAGCGTCAAGGAACTGCTGGGCATCACAATCATGGAGCGATCAAGCTTCGAAAAAAACC
CCATCGACTTTCTCGAGGCGAAAGGATATAAAGAGGTCAAAAAAGACCTCATCATTAAGCTTCCC
AAGTACTCTCTCTTTGAGCTTGAAAACGGCCGGAAACGAATGCTCGCTAGTGCGGGCGAGCTGC
AGAAAGGTAACGAGCTGGCACTGCCCTCTAAATACGTTAATTTCTTGTATCTGGCCAGCCACTAT
GAAAAGCTCAAAGGGTCTCCCGAAGATAATGAGCAGAAGCAGCTGTTCGTGGAACAACACAAAC
ACTACCTTGATGAGATCATCGAGCAAATAAGCGAATTCTCCAAAAGAGTGATCCTCGCCGACGCT
AACCTCGATAAGGTGCTTTCTGCTTACAATAAGCACAGGGATAAGCCCATCAGGGAGCAGGCAG
AAAACATTATCCACTTGTTTACTCTGACCAACTTGGGCGCGCCTGCAGCCTTCAAGTACTTCGACA
CCACCATAGACAGAAAGCGGTACACCTCTACAAAGGAGGTCCTGGACGCCACACTGATTCATCA
GTCAATTACGGGGCTCTATGAAACAAGAATCGACCTCTCTCAGCTCGGTGGAGACAGCAGGGCT
GACCCCAAGAAGAAGAGGAAGGTGTCGCCAGGGATCCGTCGACTTGACGCGTTGATATCAACAA
GTTTGTACAAAAAAGCAGGCTACAAAGAGGCCAGCGGTTCCGGACGGGCTGACGCATTGGACGA
TTTTGATCTGGATATGCTGGGAAGTGACGCCCTCGATGATTTTGACCTTGACATGCTTGGTTCGG
ATGCCCTTGATGACTTTGACCTCGACATGCTCGGCAGTGACGCCCTTGATGATTTCGACCTGGAC
ATGCTGATTAACTCTAGAAGTTCCGGATCTCCGAAAAAGAAACGCAAAGTTGGTAGCCAGTACCT
GCCCGACACCGACGACCGGCACCGGATCGAGGAAAAGCGGAAGCGGACCTACGAGACATTCAA

GAGCATCATGAAGAAGTCCCCCTTCAGCGGCCCCACCGACCCTAGACCTCCACCTAGAAGAATC
GCCGTGCCCAGCAGATCCAGCGCCAGCGTGCCAAAACCTGCCCCCCAGCCTTACCCCTTCACCA
GCAGCCTGAGCACCATCAACTACGACGAGTTCCCTACCATGGTGTTCCCCAGCGGCCAGATCTC
TCAGGCCTCTGCTCTGGCTCCAGCCCCTCCTCAGGTGCTGCCTCAGGCTCCTGCTCCTGCACCA
GCTCCAGCCATGGTGTCTGCACTGGCTCAGGCACCAGCACCCGTGCCTGTGCTGGCTCCTGGA
CCTCCACAGGCTGTGGCTCCACCAGCCCCTAAACCTACACAGGCCGGCGAGGGCACACTGTCT
GAAGCTCTGCTGCAGCTGCAGTTCGACGACGAGGATCTGGGAGCCCTGCTGGGAAACAGCACC
GATCCTGCCGTGTTCACCGACCTGGCCAGCGTGGACAACAGCGAGTTCCAGCAGCTGCTGAACC
AGGGCATCCCTGTGGCCCCTCACACCACCGAGCCCATGCTGATGGAATACCCCGAGGCCATCAC
CCGGCTCGTGACAGGCGCTCAGAGGCCTCCTGATCCAGCTCCTGCCCCTCTGGGAGCACCAGG
CCTGCCTAATGGACTGCTGTCTGGCGACGAGGACTTCAGCTCTATCGCCGATATGGATTTCTCAG
CCTTGCTGGGCTCTGGCAGCGGCAGCCGGGATTCCAGGGAAGGGATGTTTTTGCCGAAGCCTG
AGGCCGGCTCCGCTATTAGTGACGTGTTTGAGGGCCGCGAGGTGTGCCAGCCAAAACGAATCC
GGCCATTTCATCCTCCAGGAAGTCCATGGGCCAACCGCCCACTCCCCGCCAGCCTCGCACCAAC
ACCAACCGGTCCAGTACATGAGCCAGTCGGGTCACTGACCCCGGCACCAGTCCCTCAGCCACTG
GATCCAGCGCCCGCAGTGACTCCCGAGGCCAGTCACCTGTTGGAGGATCCCGATGAAGAGACG
AGCCAGGCTGTCAAAGCCCTTCGGGAGATGGCCGATACTGTGATTCCCCAGAAGGAAGAGGCTG
CAATCTGTGGCCAAATGGACCTTTCCCATCCGCCCCCAAGGGGCCATCTGGATGAGCTGACAAC
CACACTTGAGTCCATGACCGAGGATCTGAACCTGGACTCACCCCTGACCCCGGAATTGAACGAG
ATTCTGGATACCTTCCTGAACGACGAGTGCCTCTTGCATGCCATGCATATCAGCACAGGACTGTC
CATCTTCGACACATCTCTGTTTTGACAATAAAATATCTTTATTTTCATTACATCTGTGTGTTGGTTTT
TTGTGTCTCGAGTTAAGGGCGAATTCCCGATTAGGATCTTCCTAGAGCATGGCTACGTAGATAAG
TAGCATGGCGGGTTAATCATTAACTACAAGGAACCCCTAGTGATGGAGTTGGCCACTCCCTCTCT
GCGCGCTCGCTCGCTCACTGAGGCCGGGCGACCAAAGGTCGCCCGACGCCCGGGCTTTGCCC
GGGCGGCCTCAGTGAGCGAGCGAGCGCGCAGCCTTAATTAACCTAATTCACTGGCCGTCGTTTT
ACAACGTCGTGACTGGGAAAACCCTGGCGTTACCCAACTTAATCGCCTTGCAGCACATCCCCCTT
TCGCCAGCTGGCGTAATAGCGAAGAGGCCCGCACCGATCGCCCTTCCCAACAGTTGCGCAGCCT
GAATGGCGAATGGGACGCGCCCTGTAGCGGCGCATTAAGCGCGGCGGGTGTGGTGGTTACGCG
CAGCGTGACCGCTACACTTGCCAGCGCCCTAGCGCCCGCTCCTTTCGCTTTCTTCCCTTCCTTTC
TCGCCACGTTCGCCGGCTTTCCCCGTCAAGCTCTAAATCGGGGGCTCCCTTTAGGGTTCCGATTT
AGTGCTTTACGGCACCTCGACCCCAAAAAACTTGATTAGGGTGATGGTTCACGTAGTGGGCCATC
GCCCCGATAGACGGTTTTTCGCCCTTTGACGCTGGAGTTCACGTTCCTCAATAGTGGACTCTTGT
TCCAAACTGGAACAACACTCAACCCTATCTCGGTCTATTCTTTTGATTTATAAGGGATTTTCCGAT
TTCGGCCTATTGGTTAAAAAATGAGCTGATTTAACAAAAATTTAACGCGAATTTTAACAAAATATTA
ACGTTTATAATTTCAGGTGGCATCTTTCGGGGAAATGTGCGCGGAACCCCTATTTGTTTATTTTTC
TAAATACATTCAAATATGTATCCGCTCATGAGACAATAACCCTGATAAATGCTTCAATAATATTGAA
AAAGGAAGAGTATGAGTATTCAACATTTCCGTGTCGCCCTTATTCCCTTTTTTGCGGCATTTTGCC
TTCCTGTTTTTGCTCACCCAGAAACGCTGGTGAAAGTAAAAGATGCTGAAGATCAGTTGGGTGCA
CGAGTGGGTTACATCGAACTGGATCTCAATAGTGGTAAGATCCTTGAGAGTTTTCGCCCCGAAGA
ACGTTTTCCAATGATGAGCACTTTTAAAGTTCTGCTATGTGGCGCGGTATTATCCCGTATTGACGC
CGGGCAAGAGCAACTCGGTCGCCGCATACACTATTCTCAGAATGACTTGGTTGAGTACTCACCAG
TCACAGAAAAGCATCTTACGGATGGCATGACAGTAAGAGAATTATGCAGTGCTGCCATAACCATG
AGTGATAACACTGCGGCCAACTTACTTCTGACAACGATCGGAGGACCGAAGGAGCTAACCGCTTT
TTTGCACAACATGGGGGATCATGTAACTCGCCTTGATCGTTGGGAACCGGAGCTGAATGAAGCC
ATACCAAACGACGAGCGTGACACCACGATGCCTGTAGTAATGGTAACAACGTTGCGCAAACTATT
AACTGGCGAACTACTTACTCTAGCTTCCCGGCAACAATTAATAGACTGGATGGAGGCGGATAAAG
TTGCAGGACCACTTCTGCGCTCGGCCCTTCCGGCTGGCTGGTTTATTGCTGATAAATCTGGAGCC
GGTGAGCGTGGGTCTCGCGGTATCATTGCAGCACTGGGGCCAGATGGTAAGCCCTCCCGTATC
GTAGTTATCTACACGACGGGGAGTCAGGCAACTATGGATGAACGAAATAGACAGATCGCTGAGAT
AGGTGCCTCACTGATTAAGCATTGGTAACTGTCAGACCAAGTTTACTCATATATACTTTAGATTGA
TTTAAAACTTCATTTTTAATTTAAAAGGATCTAGGTGAAGATCCTTTTTGATAATCTCATGACCAAAA
TCCCTTAACGTGAGTTTTCGTTCCACTGAGCGTCAGACCCCGTAGAAAAGATCAAAGGATCTTCTT
GAGATCCTTTTTTTCTGCGCGTAATCTGCTGCTTGCAAACAAAAAAACCACCGCTACCAGCGGTG
GTTTGTTTGCCGGATCAAGAGCTACCAACTCTTTTTCCGAAGGTAACTGGCTTCAGCAGAGCGCA
GATACCAAATACTGTCCTTCTAGTGTAGCCGTAGTTAGGCCACCACTTCAAGAACTCTGTAGCAC
CGCCTACATACCTCGCTCTGCTAATCCTGTTACCAGTGGCTGCTGCCAGTGGCGATAAGTCGTGT
CTTACCGGGTTGGACTCAAGACGATAGTTACCGGATAAGGCGCAGCGGTCGGGCTGAACGGGG

GGTTCGTGCACACAGCCCAGCTTGGAGCGAACGACCTACACCGAACTGAGATACCTACAGCGTG
AGCTATGAGAAAGCGCCACGCTTCCCGAAGGGAGAAAGGCGGACAGGTATCCGGTAAGCGGCA
GGGTCGGAACAGGAGAGCGCACGAGGGAGCTTCCAGGGGGAAACGCCTGGTATCTTTATAGTC
CTGTCGGGTTTCGCCACCTCTGACTTGAGCGTCGATTTTTGTGATGCTCGTCAGGGGGGCGGAG
CCTATGGAAAAACGCCAGCAACGCGGCCTTTTTACGGTTCCTGGCCTTTTGCTGCGGTTTTGCTC
ACATGTTCTTTCCTGCGTTATCCCCTGATTCTGTGGATAACCGTATTACCGCCTTTGAGTGAGCTG
ATACCGCTCGCCGCAGCCGAACGACCGAGCGCAGCGAGTCAGTGAGCGAGGAAGCGGAAGAG
CGCCCAATACGCAAACCGCCTCTCCCCGCGCGTTGGCCGATTCATTAATGCAGCTGGCACGACA
GGTTTCCCGACTGGAAAGCGGGCAGTGAGCGCAACGCAATTAATGTGAGTTAGCTCACTCATTA
GGCACCCCAGGCTTTACACTTTATGCTTCCGGCTCGTATGTTGTGTGGAATTGTGAGCGGATAAC
AATTTCACA

3. pAAV-sglacZ-RHO-5’Cas9-SDS-BD10-SV40pA (6351 bp)

CAGGAAACAGCTATGACCATGATTACGCCAGATTTAATTAAGGCTGCGCGCTCGCTCGCTCACTG
AGGCCGCCCGGGCAAAGCCCGGGCGTCGGGCGACCTTTGGTCGCCCGGCCTCAGTGAGCGAG
CGAGCGCGCAGAGAGGGAGTGGCCAACTCCATCACTAGGGGTTCCTTGTAGTTAATGATTAACC
CGCCATGCTACTTATCTACGTAGCCATGCTCTAGGAAGATCGGAATTCGCCCTTAAGGGCGCGC
CGTTTAAACGAGGGCCTATTTCCCATGATTCCTTCATATTTGCATATACGATACAAGGCTGTTAGA
GAGATAATTAGAATTAATTTGACTGTAAACACAAAGATATTAGTACAAAATACGTGACGTAGAAAGT
AATAATTTCTTGGGTAGTTTGCAGTTTTAAAATTATGTTTTAAAATGGACTATCATATGCTTACCGTA
ACTTGAAAGTATTTCGATTTCTTGGCTTTATATATCTTGTGGAAAGGACGAAACACCGTCTGACCG
ATGATCCGCGCGTTTTAGAGCTAGAAATAGCAAGTTAAAATAAGGCTAGTCCGTTATCAACTTGAA
AAAGTGGCACCGAGTCGGTGCTTTTTTTTATTTAAATAGCTAGCCCTCTCCTCCCTGACCTCAGGC
TTCCTCCTAGTGTCACCTTGGCCCCTCTTAGAAGCCAATTAGGCCCTCAGTTTCTGCAGCGGGGA
TTAATATGATTATGAACACCCCCAATCTCCCAGATGCTGATTCAGCCAGGAGCTTAGGAGGGGGA
GGTCACTTTATAAGGGTCTGGGGGGGTCAGAACCCAGAGTCATCGGTACCACCGGTCGCCACCA
TGGCCCCAAAGAAGAAGCGGAAGGTCGGTATCCACGGAGTCCCAGCAGCCGACAAGAAGTACT
CCATTGGGCTCGATATCGGCACAAACAGCGTCGGCTGGGCCGTCATTACGGACGAGTACAAGGT
GCCGAGCAAAAAATTCAAAGTTCTGGGCAATACCGATCGCCACAGCATAAAGAAGAACCTCATTG
GCGCCCTCCTGTTCGACTCCGGGGAAACGGCCGAAGCCACGCGGCTCAAAAGAACAGCACGGC
GCAGATATACCCGCAGAAAGAATCGGATCTGCTACCTGCAGGAGATCTTTAGTAATGAGATGGCT
AAGGTGGATGACTCTTTCTTCCATAGGCTGGAGGAGTCCTTTTTGGTGGAGGAGGATAAAAAGCA
CGAGCGCCACCCAATCTTTGGCAATATCGTGGACGAGGTGGCGTACCATGAAAAGTACCCAACC
ATATATCATCTGAGGAAGAAGCTTGTAGACAGTACTGATAAGGCTGACTTGCGGTTGATCTATCTC
GCGCTGGCGCATATGATCAAATTTCGGGGACACTTCCTCATCGAGGGGGACCTGAACCCAGACA
ACAGCGATGTCGACAAACTCTTTATCCAACTGGTTCAGACTTACAATCAGCTTTTCGAAGAGAACC
CGATCAACGCATCCGGAGTTGACGCCAAAGCAATCCTGAGCGCTAGGCTGTCCAAATCCCGGCG
GCTCGAAAACCTCATCGCACAGCTCCCTGGGGAGAAGAAGAACGGCCTGTTTGGTAATCTTATC
GCCCTGTCACTCGGGCTGACCCCCAACTTTAAATCTAACTTCGACCTGGCCGAAGATGCCAAGCT
TCAACTGAGCAAAGACACCTACGATGATGATCTCGACAATCTGCTGGCCCAGATCGGCGACCAG
TACGCAGACCTTTTTTTGGCGGCAAAGAACCTGTCAGACGCCATTCTGCTGAGTGATATTCTGCG
AGTGAACACGGAGATCACCAAAGCTCCGCTGAGCGCTAGTATGATCAAGCGCTATGATGAGCAC
CACCAAGACTTGACTTTGCTGAAGGCCCTTGTCAGACAGCAACTGCCTGAGAAGTACAAGGAAAT
TTTCTTCGATCAGTCTAAAAATGGCTACGCCGGATACATTGACGGCGGAGCAAGCCAGGAGGAAT
TTTACAAATTTATTAAGCCCATCTTGGAAAAAATGGACGGCACCGAGGAGCTGCTGGTAAAGCTT
AACAGAGAAGATCTGTTGCGCAAACAGCGCACTTTCGACAATGGAAGCATCCCCCACCAGATTCA
CCTGGGCGAACTGCACGCTATCCTCAGGCGGCAAGAGGATTTCTACCCCTTTTTGAAAGATAACA
GGGAAAAGATTGAGAAAATCCTCACATTTCGGATACCCTACTATGTAGGCCCCCTCGCCCGGGG
AAATTCCAGATTCGCGTGGATGACTCGCAAATCAGAAGAGACCATCACTCCCTGGAACTTCGAGG
AAGTCGTGGATAAGGGGGCCTCTGCCCAGTCCTTCATCGAAAGGATGACTAACTTTGATAAAAAT
CTGCCTAACGAAAAGGTGCTTCCTAAACACTCTCTGCTGTACGAGTACTTCACAGTTTATAACGAG
CTCACCAAGGTCAAATACGTCACAGAAGGGATGAGAAAGCCAGCATTCCTGTCTGGAGAGCAGA
AGAAAGCTATCGTGGACCTCCTCTTCAAGACGAACCGGAAAGTTACCGTGAAACAGCTCAAAGAA
GACTATTTCAAAAAGATTGAATGTTTCGACTCTGTTGAAATCAGCGGAGTGGAGGATCGCTTCAAC
GCATCCCTGGGAACGTATCACGATCTCCTGAAAATCATTAAAGACAAGGACTTCCTGGACAATGA
GGAGAACGAGGACATTCTTGAGGACATTGTCCTCACCCTTACGTTGTTTGAAGATAGGGAGATGA
TTGAAGAACGCTTGAAAACTTACGCTCATCTCTTCGACGACAAAGTCATGAAACAGCTCAAGAGG

CGCCGATATACAGGATGGGGGCGGCTGTCAAGAAAACTGATCAATGGGATCCGAGACAAGCAGA
GTGGAAAGACAATCCTGGATTTTCTTAAGTCCGATGGATTTGCCAACCGGAACTTCATGCAGTTG
ATCCATGATGACTCTCTCACCTTTAAGGAGGACATCCAGAAAGCACAAGTTTCTGGCCAGGGGGA
CAGTCTTCACGAGCACATCGCTAATCTTGCAGGTAAGGGCACTGAGCAGAAGGGAAGAAGCTCC
GGGGGCTCTTTGTAGGGTGCGGCCGCAGTGCATCAAGGCGATCACATCAGTGAAAAAAAGCCAG
ACAGGCGGTTAAACCAACGCAGATTAAACAGCAGGATGCAAAAATTCGCAGGTGGTCAGATGGC
GGCCGCTCTAGACTCGATGAGTTTGGACAAACCACAACTAGAATGCAGTGAAAAAAATGCTTTAT
TTGTGAAATTTGTGATGCTATTGCTTTATTTGTAACCATTATAAGCTGCAATAAACAAGTTCTCGAG
TTAAGGGCGAATTCCCGATTAGGATCTTCCTAGAGCATGGCTACGTAGATAAGTAGCATGGCGGG
TTAATCATTAACTACAAGGAACCCCTAGTGATGGAGTTGGCCACTCCCTCTCTGCGCGCTCGCTC
GCTCACTGAGGCCGGGCGACCAAAGGTCGCCCGACGCCCGGGCTTTGCCCGGGCGGCCTCAG
TGAGCGAGCGAGCGCGCAGCCTTAATTAACCTAATTCACTGGCCGTCGTTTTACAACGTCGTGAC
TGGGAAAACCCTGGCGTTACCCAACTTAATCGCCTTGCAGCACATCCCCCTTTCGCCAGCTGGC
GTAATAGCGAAGAGGCCCGCACCGATCGCCCTTCCCAACAGTTGCGCAGCCTGAATGGCGAATG
GGACGCGCCCTGTAGCGGCGCATTAAGCGCGGCGGGTGTGGTGGTTACGCGCAGCGTGACCG
CTACACTTGCCAGCGCCCTAGCGCCCGCTCCTTTCGCTTTCTTCCCTTCCTTTCTCGCCACGTTC
GCCGGCTTTCCCCGTCAAGCTCTAAATCGGGGGCTCCCTTTAGGGTTCCGATTTAGTGCTTTACG
GCACCTCGACCCCAAAAAACTTGATTAGGGTGATGGTTCACGTAGTGGGCCATCGCCCCGATAG
ACGGTTTTTCGCCCTTTGACGCTGGAGTTCACGTTCCTCAATAGTGGACTCTTGTTCCAAACTGG
AACAACACTCAACCCTATCTCGGTCTATTCTTTTGATTTATAAGGGATTTTTCCGATTTCGGCCTAT
TGGTTAAAAAATGAGCTGATTTAACAAAAATTTAACGCGAATTTTAACAAAATATTAACGTTTATAAT
TTCAGGTGGCATCTTTCGGGGAAATGTGCGCGGAACCCCTATTTGTTTATTTTTCTAAATACATTC
AAATATGTATCCGCTCATGAGACAATAACCCTGATAAATGCTTCAATAATATTGAAAAAGGAAGAG
TATGAGTATTCAACATTTCCGTGTCGCCCTTATTCCCTTTTTTGCGGCATTTTGCCTTCCTGTTTTT
GCTCACCCAGAAACGCTGGTGAAAGTAAAAGATGCTGAAGATCAGTTGGGTGCACGAGTGGGTT
ACATCGAACTGGATCTCAATAGTGGTAAGATCCTTGAGAGTTTTCGCCCCGAAGAACGTTTTCCA
ATGATGAGCACTTTTAAAGTTCTGCTATGTGGCGCGGTATTATCCCGTATTGACGCCGGGCAAGA
GCAACTCGGTCGCCGCATACACTATTCTCAGAATGACTTGGTTGAGTACTCACCAGTCACAGAAA
AGCATCTTACGGATGGCATGACAGTAAGAGAATTATGCAGTGCTGCCATAACCATGAGTGATAAC
ACTGCGGCCAACTTACTTCTGACAACGATCGGAGGACCGAAGGAGCTAACCGCTTTTTTGCACAA
CATGGGGGATCATGTAACTCGCCTTGATCGTTGGGAACCGGAGCTGAATGAAGCCATACCAAAC
GACGAGCGTGACACCACGATGCCTGTAGTAATGGTAACAACGTTGCGCAAACTATTAACTGGCGA
ACTACTTACTCTAGCTTCCCGGCAACAATTAATAGACTGGATGGAGGCGGATAAAGTTGCAGGAC
CACTTCTGCGCTCGGCCCTTCCGGCTGGCTGGTTTATTGCTGATAAATCTGGAGCCGGTGAGCG
TGGGTCTCGCGGTATCATTGCAGCACTGGGGCCAGATGGTAAGCCCTCCCGTATCGTAGTTATC
TACACGACGGGGAGTCAGGCAACTATGGATGAACGAAATAGACAGATCGCTGAGATAGGTGCCT
CACTGATTAAGCATTGGTAACTGTCAGACCAAGTTTACTCATATATACTTTAGATTGATTTAAAACT
TCATTTTTAATTTAAAAGGATCTAGGTGAAGATCCTTTTTGATAATCTCATGACCAAAATCCCTTAA
CGTGAGTTTTCGTTCCACTGAGCGTCAGACCCCGTAGAAAAGATCAAAGGATCTTCTTGAGATCC
TTTTTTTCTGCGCGTAATCTGCTGCTTGCAAACAAAAAAACCACCGCTACCAGCGGTGGTTTGTTT
GCCGGATCAAGAGCTACCAACTCTTTTTCCGAAGGTAACTGGCTTCAGCAGAGCGCAGATACCAA
ATACTGTCCTTCTAGTGTAGCCGTAGTTAGGCCACCACTTCAAGAACTCTGTAGCACCGCCTACA
TACCTCGCTCTGCTAATCCTGTTACCAGTGGCTGCTGCCAGTGGCGATAAGTCGTGTCTTACCGG
GTTGGACTCAAGACGATAGTTACCGGATAAGGCGCAGCGGTCGGGCTGAACGGGGGGTTCGTG
CACACAGCCCAGCTTGGAGCGAACGACCTACACCGAACTGAGATACCTACAGCGTGAGCTATGA
GAAAGCGCCACGCTTCCCGAAGGGAGAAAGGCGGACAGGTATCCGGTAAGCGGCAGGGTCGGA
ACAGGAGAGCGCACGAGGGAGCTTCCAGGGGGAAACGCCTGGTATCTTTATAGTCCTGTCGGGT
TTCGCCACCTCTGACTTGAGCGTCGATTTTTGTGATGCTCGTCAGGGGGGCGGAGCCTATGGAA
AAACGCCAGCAACGCGGCCTTTTTACGGTTCCTGGCCTTTTGCTGCGGTTTTGCTCACATGTTCT
TTCCTGCGTTATCCCCTGATTCTGTGGATAACCGTATTACCGCCTTTGAGTGAGCTGATACCGCT
CGCCGCAGCCGAACGACCGAGCGCAGCGAGTCAGTGAGCGAGGAAGCGGAAGAGCGCCCAAT
ACGCAAACCGCCTCTCCCCGCGCGTTGGCCGATTCATTAATGCAGCTGGCACGACAGGTTTCCC
GACTGGAAAGCGGGCAGTGAGCGCAACGCAATTAATGTGAGTTAGCTCACTCATTAGGCACCCC
AGGCTTTACACTTTATGCTTCCGGCTCGTATGTTGTGTGGAATTGTGAGCGGATAACAATTTCACA

4. pAAV-RHO-BD10-SAS-3'Cas9-VPR-synpA (7285 bp)

CAGGAAACAGCTATGACCATGATTACGCCAGATTTAATTAAGGCTGCGCGCTCGCTCGCTCACTG
AGGCCGCCCGGGCAAAGCCCGGGCGTCGGGCGACCTTTGGTCGCCCGGCCTCAGTGAGCGAG
CGAGCGCGCAGAGAGGGAGTGGCCAACTCCATCACTAGGGGTTCCTTGTAGTTAATGATTAACC
CGCCATGCTACTTATCTACGTAGCCATGCTCTAGGAAGATCGGAATTCGCCCTTAAGGGCGCGC
CGTTTAAATAGCTAGCCCTCTCCTCCCTGACCTCAGGCTTCCTCCTAGTGTCACCTTGGCCCCTC
TTAGAAGCCAATTAGGCCCTCAGTTTCTGCAGCGGGGATTAATATGATTATGAACACCCCCAATCT
CCCAGATGCTGATTCAGCCAGGAGCTTAGGAGGGGGAGGTCACTTTATAAGGGTCTGGGGGGG
TCAGAACCCAGAGTCATCGGTACCACCGGTCGACTAGAGGATCCAGGTGCGGCCGCCATCTGAC
CACCTGCGAATTTTTGCATCCTGCTGTTTAATCTGCGTTGGTTTAACCGCCTGTCTGGCTTTTTTT
CACTGATGTGATCGCCTTGATGCACTGCGGCCGCCAACGAGTCTTTTGTCATCTACAGGTAGCCC
AGCTATCAAAAAGGGAATACTGCAGACCGTTAAGGTCGTGGATGAACTCGTCAAAGTAATGGGAA
GGCATAAGCCCGAGAATATCGTTATCGAGATGGCCCGAGAGAACCAAACTACCCAGAAGGGACA
GAAGAACAGTAGGGAAAGGATGAAGAGGATTGAAGAGGGTATAAAAGAACTGGGGTCCCAAATC
CTTAAGGAACACCCAGTTGAAAACACCCAGCTTCAGAATGAGAAGCTCTACCTGTACTACCTGCA
GAACGGCAGGGACATGTACGTGGATCAGGAACTGGACATCAATCGGCTCTCCGACTACGACGTG
GATCATATCGTGCCCCAGTCTTTTCTCAAAGATGATTCTATTGATAATAAAGTGTTGACAAGATCC
GATAAAAATAGAGGGAAGAGTGATAACGTCCCCTCAGAAGAAGTTGTCAAGAAAATGAAAAATTAT
TGGCGGCAGCTGCTGAACGCCAAACTGATCACACAACGGAAGTTCGATAATCTGACTAAGGCTG
AACGAGGTGGCCTGTCTGAGTTGGATAAAGCCGGCTTCATCAAAAGGCAGCTTGTTGAGACACG
CCAGATCACCAAGCACGTGGCCCAAATTCTCGATTCACGCATGAACACCAAGTACGATGAAAATG
ACAAACTGATTCGAGAGGTGAAAGTTATTACTCTGAAGTCTAAGCTGGTCTCAGATTTCAGAAAGG
ACTTTCAGTTTTATAAGGTGAGAGAGATCAACAATTACCACCATGCGCATGATGCCTACCTGAATG
CAGTGGTAGGCACTGCACTTATCAAAAAATATCCCAAGCTTGAATCTGAATTTGTTTACGGAGACT
ATAAAGTGTACGATGTTAGGAAAATGATCGCAAAGTCTGAGCAGGAAATAGGCAAGGCCACCGCT
AAGTACTTCTTTTACAGCAATATTATGAATTTTTTCAAGACCGAGATTACACTGGCCAATGGAGAG
ATTCGGAAGCGACCACTTATCGAAACAAACGGAGAAACAGGAGAAATCGTGTGGGACAAGGGTA
GGGATTTCGCGACAGTCCGGAAGGTCCTGTCCATGCCGCAGGTGAACATCGTTAAAAAGACCGA
AGTACAGACCGGAGGCTTCTCCAAGGAAAGTATCCTCCCGAAAAGGAACAGCGACAAGCTGATC
GCACGCAAAAAAGATTGGGACCCCAAGAAATACGGCGGATTCGATTCTCCTACAGTCGCTTACAG
TGTACTGGTTGTGGCCAAAGTGGAGAAAGGGAAGTCTAAAAAACTCAAAAGCGTCAAGGAACTGC
TGGGCATCACAATCATGGAGCGATCAAGCTTCGAAAAAAACCCCATCGACTTTCTCGAGGCGAAA
GGATATAAAGAGGTCAAAAAAGACCTCATCATTAAGCTTCCCAAGTACTCTCTCTTTGAGCTTGAA
AACGGCCGGAAACGAATGCTCGCTAGTGCGGGCGAGCTGCAGAAAGGTAACGAGCTGGCACTG
CCCTCTAAATACGTTAATTTCTTGTATCTGGCCAGCCACTATGAAAAGCTCAAAGGGTCTCCCGAA
GATAATGAGCAGAAGCAGCTGTTCGTGGAACAACACAAACACTACCTTGATGAGATCATCGAGCA
AATAAGCGAATTCTCCAAAAGAGTGATCCTCGCCGACGCTAACCTCGATAAGGTGCTTTCTGCTT
ACAATAAGCACAGGGATAAGCCCATCAGGGAGCAGGCAGAAAACATTATCCACTTGTTTACTCTG
ACCAACTTGGGCGCGCCTGCAGCCTTCAAGTACTTCGACACCACCATAGACAGAAAGCGGTACA
CCTCTACAAAGGAGGTCCTGGACGCCACACTGATTCATCAGTCAATTACGGGGCTCTATGAAACA
AGAATCGACCTCTCTCAGCTCGGTGGAGACAGCAGGGCTGACCCCAAGAAGAAGAGGAAGGTGT
CGCCAGGGATCCGTCGACTTGACGCGTTGATATCAACAAGTTTGTACAAAAAAGCAGGCTACAAA
GAGGCCAGCGGTTCCGGACGGGCTGACGCATTGGACGATTTTGATCTGGATATGCTGGGAAGTG
ACGCCCTCGATGATTTTGACCTTGACATGCTTGGTTCGGATGCCCTTGATGACTTTGACCTCGAC
ATGCTCGGCAGTGACGCCCTTGATGATTTCGACCTGGACATGCTGATTAACTCTAGAAGTTCCGG
ATCTCCGAAAAAGAAACGCAAAGTTGGTAGCCAGTACCTGCCCGACACCGACGACCGGCACCGG
ATCGAGGAAAAGCGGAAGCGGACCTACGAGACATTCAAGAGCATCATGAAGAAGTCCCCCTTCA
GCGGCCCCACCGACCCTAGACCTCCACCTAGAAGAATCGCCGTGCCCAGCAGATCCAGCGCCA
GCGTGCCAAAACCTGCCCCCCAGCCTTACCCCTTCACCAGCAGCCTGAGCACCATCAACTACGA
CGAGTTCCCTACCATGGTGTTCCCCAGCGGCCAGATCTCTCAGGCCTCTGCTCTGGCTCCAGCC
CCTCCTCAGGTGCTGCCTCAGGCTCCTGCTCCTGCACCAGCTCCAGCCATGGTGTCTGCACTGG
CTCAGGCACCAGCACCCGTGCCTGTGCTGGCTCCTGGACCTCCACAGGCTGTGGCTCCACCAG
CCCCTAAACCTACACAGGCCGGCGAGGGCACACTGTCTGAAGCTCTGCTGCAGCTGCAGTTCGA
CGACGAGGATCTGGGAGCCCTGCTGGGAAACAGCACCGATCCTGCCGTGTTCACCGACCTGGC
CAGCGTGGACAACAGCGAGTTCCAGCAGCTGCTGAACCAGGGCATCCCTGTGGCCCCTCACAC
CACCGAGCCCATGCTGATGGAATACCCCGAGGCCATCACCCGGCTCGTGACAGGCGCTCAGAG
GCCTCCTGATCCAGCTCCTGCCCCTCTGGGAGCACCAGGCCTGCCTAATGGACTGCTGTCTGGC

GACGAGGACTTCAGCTCTATCGCCGATATGGATTTCTCAGCCTTGCTGGGCTCTGGCAGCGGCA
GCCGGGATTCCAGGGAAGGGATGTTTTTGCCGAAGCCTGAGGCCGGCTCCGCTATTAGTGACGT
GTTTGAGGGCCGCGAGGTGTGCCAGCCAAAACGAATCCGGCCATTTCATCCTCCAGGAAGTCCA
TGGGCCAACCGCCCACTCCCCGCCAGCCTCGCACCAACACCAACCGGTCCAGTACATGAGCCA
GTCGGGTCACTGACCCCGGCACCAGTCCCTCAGCCACTGGATCCAGCGCCCGCAGTGACTCCC
GAGGCCAGTCACCTGTTGGAGGATCCCGATGAAGAGACGAGCCAGGCTGTCAAAGCCCTTCGG
GAGATGGCCGATACTGTGATTCCCCAGAAGGAAGAGGCTGCAATCTGTGGCCAAATGGACCTTT
CCCATCCGCCCCCAAGGGGCCATCTGGATGAGCTGACAACCACACTTGAGTCCATGACCGAGGA
TCTGAACCTGGACTCACCCCTGACCCCGGAATTGAACGAGATTCTGGATACCTTCCTGAACGACG
AGTGCCTCTTGCATGCCATGCATATCAGCACAGGACTGTCCATCTTCGACACATCTCTGTTTTGAC
AATAAAATATCTTTATTTTCATTACATCTGTGTGTTGGTTTTTTGTGTCTCGAGTTAAGGGCGAATT
CCCGATTAGGATCTTCCTAGAGCATGGCTACGTAGATAAGTAGCATGGCGGGTTAATCATTAACT
ACAAGGAACCCCTAGTGATGGAGTTGGCCACTCCCTCTCTGCGCGCTCGCTCGCTCACTGAGGC
CGGGCGACCAAAGGTCGCCCGACGCCCGGGCTTTGCCCGGGCGGCCTCAGTGAGCGAGCGAG
CGCGCAGCCTTAATTAACCTAATTCACTGGCCGTCGTTTTACAACGTCGTGACTGGGAAAACCCT
GGCGTTACCCAACTTAATCGCCTTGCAGCACATCCCCCTTTCGCCAGCTGGCGTAATAGCGAAGA
GGCCCGCACCGATCGCCCTTCCCAACAGTTGCGCAGCCTGAATGGCGAATGGGACGCGCCCTG
TAGCGGCGCATTAAGCGCGGCGGGTGTGGTGGTTACGCGCAGCGTGACCGCTACACTTGCCAG
CGCCCTAGCGCCCGCTCCTTTCGCTTTCTTCCCTTCCTTTCTCGCCACGTTCGCCGGCTTTCCCC
GTCAAGCTCTAAATCGGGGGCTCCCTTTAGGGTTCCGATTTAGTGCTTTACGGCACCTCGACCCC
AAAAAACTTGATTAGGGTGATGGTTCACGTAGTGGGCCATCGCCCCGATAGACGGTTTTTCGCCC
TTTGACGCTGGAGTTCACGTTCCTCAATAGTGGACTCTTGTTCCAAACTGGAACAACACTCAACC
CTATCTCGGTCTATTCTTTTGATTTATAAGGGATTTTTCCGATTTCGGCCTATTGGTTAAAAAATGA
GCTGATTTAACAAAAATTTAACGCGAATTTTAACAAAATATTAACGTTTATAATTTCAGGTGGCATC
TTTCGGGGAAATGTGCGCGGAACCCCTATTTGTTTATTTTTCTAAATACATTCAAATATGTATCCGC
TCATGAGACAATAACCCTGATAAATGCTTCAATAATATTGAAAAAGGAAGAGTATGAGTATTCAAC
ATTTCCGTGTCGCCCTTATTCCCTTTTTTGCGGCATTTTGCCTTCCTGTTTTTGCTCACCCAGAAA
CGCTGGTGAAAGTAAAAGATGCTGAAGATCAGTTGGGTGCACGAGTGGGTTACATCGAACTGGA
TCTCAATAGTGGTAAGATCCTTGAGAGTTTTCGCCCCGAAGAACGTTTTCCAATGATGAGCACTTT
TAAAGTTCTGCTATGTGGCGCGGTATTATCCCGTATTGACGCCGGGCAAGAGCAACTCGGTCGC
CGCATACACTATTCTCAGAATGACTTGGTTGAGTACTCACCAGTCACAGAAAAGCATCTTACGGAT
GGCATGACAGTAAGAGAATTATGCAGTGCTGCCATAACCATGAGTGATAACACTGCGGCCAACTT
ACTTCTGACAACGATCGGAGGACCGAAGGAGCTAACCGCTTTTTTGCACAACATGGGGGATCAT
GTAACTCGCCTTGATCGTTGGGAACCGGAGCTGAATGAAGCCATACCAAACGACGAGCGTGACA
CCACGATGCCTGTAGTAATGGTAACAACGTTGCGCAAACTATTAACTGGCGAACTACTTACTCTA
GCTTCCCGGCAACAATTAATAGACTGGATGGAGGCGGATAAAGTTGCAGGACCACTTCTGCGCT
CGGCCCTTCCGGCTGGCTGGTTTATTGCTGATAAATCTGGAGCCGGTGAGCGTGGGTCTCGCGG
TATCATTGCAGCACTGGGGCCAGATGGTAAGCCCTCCCGTATCGTAGTTATCTACACGACGGGG
AGTCAGGCAACTATGGATGAACGAAATAGACAGATCGCTGAGATAGGTGCCTCACTGATTAAGCA
TTGGTAACTGTCAGACCAAGTTTACTCATATATACTTTAGATTGATTTAAAACTTCATTTTTAATTTA
AAAGGATCTAGGTGAAGATCCTTTTTGATAATCTCATGACCAAAATCCCTTAACGTGAGTTTTCGT
TCCACTGAGCGTCAGACCCCGTAGAAAAGATCAAAGGATCTTCTTGAGATCCTTTTTTTCTGCGC
GTAATCTGCTGCTTGCAAACAAAAAAACCACCGCTACCAGCGGTGGTTTGTTTGCCGGATCAAGA
GCTACCAACTCTTTTTCCGAAGGTAACTGGCTTCAGCAGAGCGCAGATACCAAATACTGTCCTTC
TAGTGTAGCCGTAGTTAGGCCACCACTTCAAGAACTCTGTAGCACCGCCTACATACCTCGCTCTG
CTAATCCTGTTACCAGTGGCTGCTGCCAGTGGCGATAAGTCGTGTCTTACCGGGTTGGACTCAAG
ACGATAGTTACCGGATAAGGCGCAGCGGTCGGGCTGAACGGGGGGTTCGTGCACACAGCCCAG
CTTGGAGCGAACGACCTACACCGAACTGAGATACCTACAGCGTGAGCTATGAGAAAGCGCCACG
CTTCCCGAAGGGAGAAAGGCGGACAGGTATCCGGTAAGCGGCAGGGTCGGAACAGGAGAGCGC
ACGAGGGAGCTTCCAGGGGGAAACGCCTGGTATCTTTATAGTCCTGTCGGGTTTCGCCACCTCT
GACTTGAGCGTCGATTTTTGTGATGCTCGTCAGGGGGGCGGAGCCTATGGAAAAACGCCAGCAA
CGCGGCCTTTTTACGGTTCCTGGCCTTTTGCTGCGGTTTTGCTCACATGTTCTTTCCTGCGTTATC
CCCTGATTCTGTGGATAACCGTATTACCGCCTTTGAGTGAGCTGATACCGCTCGCCGCAGCCGAA
CGACCGAGCGCAGCGAGTCAGTGAGCGAGGAAGCGGAAGAGCGCCCAATACGCAAACCGCCTC
TCCCCGCGCGTTGGCCGATTCATTAATGCAGCTGGCACGACAGGTTTCCCGACTGGAAAGCGGG
CAGTGAGCGCAACGCAATTAATGTGAGTTAGCTCACTCATTAGGCACCCCAGGCTTTACACTTTA
TGCTTCCGGCTCGTATGTTGTGTGGAATTGTGAGCGGATAACAATTTCACA

5. **pAAV-sgRho-2xsgOpn1mw-RHO-5'Cas9-SDS-BD10-SV40pA (7056 bp)**

CAGGAAACAGCTATGACCATGATTACGCCAGATTTAATTAAGGCTGCGCGCTCGCTCGCTCACTG
AGGCCGCCCGGGCAAAGCCCGGGCGTCGGGCGACCTTTGGTCGCCCGGCCTCAGTGAGCGAG
CGAGCGCGCAGAGAGGGAGTGGCCAACTCCATCACTAGGGGTTCCTTGTAGTTAATGATTAACC
CGCCATGCTACTTATCTACGTAGCCATGCTCTAGGAAGATCGGAATTCGCCCTTAAGGGCGCGC
CGTTTAAACGAGGGCCTATTTCCCATGATTCCTTCATATTTGCATATACGATACAAGGCTGTTAGA
GAGATAATTGGAATTAATTTGACTGTAAACACAAAGATATTAGTACAAAATACGTGACGTAGAAAG
TAATAATTTCTTGGGTAGTTTGCAGTTTTAAAATTATGTTTTAAAATGGACTATCATATGCTTACCGT
AACTTGAAAGTATTTCGATTTCTTGGCTTTATATATCTTGTGGAAAGGACGAAACACCGTACGGTG
ACGTAGAGCGTGGTTTTAGAGCTAGAAATAGCAAGTTAAAATAAGGCTAGTCCGTTATCAACTTGA
AAAAGTGGCACCGAGTCGGTGCTTTTTTTGTATACGAGGGCCTATTTCCCATGATTCCTTCATATT
TGCATATACGATACAAGGCTGTTAGAGAGATAATTGGAATTAATTTGACTGTAAACACAAAGATAT
TAGTACAAAATACGTGACGTAGAAAGTAATAATTTCTTGGGTAGTTTGCAGTTTTAAAATTATGTTT
TAAAATGGACTATCATATGCTTACCGTAACTTGAAAGTATTTCGATTTCTTGGCTTTATATATCTTG
TGGAAAGGACGAAACACCGGGGCCTTTAAGGTAGTTTTAGAGCTAGAAATAGCAAGTTAAAATAA
GGCTAGTCCGTTATCAACTTGAAAAAGTGGCACCGAGTCGGTGCTTTTTTTGTTAACGAGGGCCT
ATTTCCCATGATTCCTTCATATTTGCATATACGATACAAGGCTGTTAGAGAGATAATTGGAATTAAT
TTGACTGTAAACACAAAGATATTAGTACAAAATACGTGACGTAGAAAGTAATAATTTCTTGGGTAG
TTTGCAGTTTTAAAATTATGTTTTAAAATGGACTATCATATGCTTACCGTAACTTGAAAGTATTTCGA
TTTCTTGGCTTTATATATCTTGTGGAAAGGACGAAACACCGCCACCCCTGTGGATGTTTTAGAGCT
AGAAATAGCAAGTTAAAATAAGGCTAGTCCGTTATCAACTTGAAAAAGTGGCACCGAGTCGGTGC
TTTTTTTATTTAAATAGCTAGCCCTCTCCTCCCTGACCTCAGGCTTCCTCCTAGTGTCACCTTGGC
CCCTCTTAGAAGCCAATTAGGCCCTCAGTTTCTGCAGCGGGGATTAATATGATTATGAACACCCC
CAATCTCCCAGATGCTGATTCAGCCAGGAGCTTAGGAGGGGGAGGTCACTTTATAAGGGTCTGG
GGGGGTCAGAACCCAGAGTCATCGGTACCACCGGTCGCCACCATGGCCCCAAAGAAGAAGCGG
AAGGTCGGTATCCACGGAGTCCCAGCAGCCGACAAGAAGTACTCCATTGGGCTCGATATCGGCA
CAAACAGCGTCGGCTGGGCCGTCATTACGGACGAGTACAAGGTGCCGAGCAAAAAATTCAAAGT
TCTGGGCAATACCGATCGCCACAGCATAAAGAAGAACCTCATTGGCGCCCTCCTGTTCGACTCC
GGGGAAACGGCCGAAGCCACGCGGCTCAAAAGAACAGCACGGCGCAGATATACCCGCAGAAAG
AATCGGATCTGCTACCTGCAGGAGATCTTTAGTAATGAGATGGCTAAGGTGGATGACTCTTTCTT
CCATAGGCTGGAGGAGTCCTTTTTGGTGGAGGAGGATAAAAAGCACGAGCGCCACCCAATCTTT
GGCAATATCGTGGACGAGGTGGCGTACCATGAAAAGTACCCAACCATATATCATCTGAGGAAGAA
GCTTGTAGACAGTACTGATAAGGCTGACTTGCGGTTGATCTATCTCGCGCTGGCGCATATGATCA
AATTTCGGGGACACTTCCTCATCGAGGGGGACCTGAACCCAGACAACAGCGATGTCGACAAACT
CTTTATCCAACTGGTTCAGACTTACAATCAGCTTTTCGAAGAGAACCCGATCAACGCATCCGGAG
TTGACGCCAAAGCAATCCTGAGCGCTAGGCTGTCCAAATCCCGGCGGCTCGAAAACCTCATCGC
ACAGCTCCCTGGGGAGAAGAAGAACGGCCTGTTTGGTAATCTTATCGCCCTGTCACTCGGGCTG
ACCCCCAACTTTAAATCTAACTTCGACCTGGCCGAAGATGCCAAGCTTCAACTGAGCAAAGACAC
CTACGATGATGATCTCGACAATCTGCTGGCCCAGATCGGCGACCAGTACGCAGACCTTTTTTTGG
CGGCAAAGAACCTGTCAGACGCCATTCTGCTGAGTGATATTCTGCGAGTGAACACGGAGATCAC
CAAAGCTCCGCTGAGCGCTAGTATGATCAAGCGCTATGATGAGCACCACCAAGACTTGACTTTGC
TGAAGGCCCTTGTCAGACAGCAACTGCCTGAGAAGTACAAGGAAATTTTCTTCGATCAGTCTAAA
AATGGCTACGCCGGATACATTGACGGCGGAGCAAGCCAGGAGGAATTTTACAAATTTATTAAGCC
CATCTTGGAAAAAATGGACGGCACCGAGGAGCTGCTGGTAAAGCTTAACAGAGAAGATCTGTTG
CGCAAACAGCGCACTTTCGACAATGGAAGCATCCCCCACCAGATTCACCTGGGCGAACTGCACG
CTATCCTCAGGCGGCAAGAGGATTTCTACCCCTTTTTGAAAGATAACAGGGAAAAGATTGAGAAA
ATCCTCACATTTCGGATACCCTACTATGTAGGCCCCCTCGCCCGGGGAAATTCCAGATTCGCGTG
GATGACTCGCAAATCAGAAGAGACCATCACTCCCTGGAACTTCGAGGAAGTCGTGGATAAGGGG
GCCTCTGCCCAGTCCTTCATCGAAAGGATGACTAACTTTGATAAAAATCTGCCTAACGAAAAGGT
GCTTCCTAAACACTCTCTGCTGTACGAGTACTTCACAGTTTATAACGAGCTCACCAAGGTCAAATA
CGTCACAGAAGGGATGAGAAAGCCAGCATTCCTGTCTGGAGAGCAGAAGAAAGCTATCGTGGAC
CTCCTCTTCAAGACGAACCGGAAAGTTACCGTGAAACAGCTCAAAGAAGACTATTTCAAAAAGATT
GAATGTTTCGACTCTGTTGAAATCAGCGGAGTGGAGGATCGCTTCAACGCATCCCTGGGAACGTA
TCACGATCTCCTGAAAATCATTAAAGACAAGGACTTCCTGGACAATGAGGAGAACGAGGACATTC
TTGAGGACATTGTCCTCACCCTTACGTTGTTTGAAGATAGGGAGATGATTGAAGAACGCTTGAAA
ACTTACGCTCATCTCTTCGACGACAAAGTCATGAAACAGCTCAAGAGGCGCCGATATACAGGATG
GGGGCGGCTGTCAAGAAAACTGATCAATGGGATCCGAGACAAGCAGAGTGGAAAGACAATCCTG

GATTTTCTTAAGTCCGATGGATTTGCCAACCGGAACTTCATGCAGTTGATCCATGATGACTCTCTC
ACCTTTAAGGAGGACATCCAGAAAGCACAAGTTTCTGGCCAGGGGGACAGTCTTCACGAGCACA
TCGCTAATCTTGCAGGTAAGGGCACTGAGCAGAAGGGAAGAAGCTCCGGGGGCTCTTTGTAGGG
TGCGGCCGCAGTGCATCAAGGCGATCACATCAGTGAAAAAAAGCCAGACAGGCGGTTAAACCAA
CGCAGATTAAACAGCAGGATGCAAAAATTCGCAGGTGGTCAGATGGCGGCCGCTCTAGACTCGA
TGAGTTTGGACAAACCACAACTAGAATGCAGTGAAAAAAATGCTTTATTTGTGAAATTTGTGATGC
TATTGCTTTATTTGTAACCATTATAAGCTGCAATAAACAAGTTCTCGAGTTAAGGGCGAATTCCCG
ATTAGGATCTTCCTAGAGCATGGCTACGTAGATAAGTAGCATGGCGGGTTAATCATTAACTACAA
GGAACCCCTAGTGATGGAGTTGGCCACTCCCTCTCTGCGCGCTCGCTCGCTCACTGAGGCCGG
GCGACCAAAGGTCGCCCGACGCCCGGGCTTTGCCCGGGCGGCCTCAGTGAGCGAGCGAGCGC
GCAGCCTTAATTAACCTAATTCACTGGCCGTCGTTTTACAACGTCGTGACTGGGAAAACCCTGGC
GTTACCCAACTTAATCGCCTTGCAGCACATCCCCCTTTCGCCAGCTGGCGTAATAGCGAAGAGGC
CCGCACCGATCGCCCTTCCCAACAGTTGCGCAGCCTGAATGGCGAATGGGACGCGCCCTGTAG
CGGCGCATTAAGCGCGGCGGGTGTGGTGGTTACGCGCAGCGTGACCGCTACACTTGCCAGCGC
CCTAGCGCCCGCTCCTTTCGCTTTCTTCCCTTCCTTTCTCGCCACGTTCGCCGGCTTTCCCCGTC
AAGCTCTAAATCGGGGGCTCCCTTTAGGGTTCCGATTTAGTGCTTTACGGCACCTCGACCCCAAA
AAACTTGATTAGGGTGATGGTTCACGTAGTGGGCCATCGCCCCGATAGACGGTTTTTCGCCCTTT
GACGCTGGAGTTCACGTTCCTCAATAGTGGACTCTTGTTCCAAACTGGAACAACACTCAACCCTA
TCTCGGTCTATTCTTTTGATTTATAAGGGATTTTCCGATTTCGGCCTATTGGTTAAAAAATGAGCT
GATTTAACAAAAATTTAACGCGAATTTTAACAAAATATTAACGTTTATAATTTCAGGTGGCATCTTTC
GGGGAAATGTGCGCGGAACCCCTATTTGTTTATTTTTCTAAATACATTCAAATATGTATCCGCTCA
TGAGACAATAACCCTGATAAATGCTTCAATAATATTGAAAAAGGAAGAGTATGAGTATTCAACATTT
CCGTGTCGCCCTTATTCCCTTTTTTGCGGCATTTTGCCTTCCTGTTTTTGCTCACCCAGAAACGCT
GGTGAAAGTAAAAGATGCTGAAGATCAGTTGGGTGCACGAGTGGGTTACATCGAACTGGATCTCA
ATAGTGGTAAGATCCTTGAGAGTTTTCGCCCCGAAGAACGTTTTCCAATGATGAGCACTTTTAAAG
TTCTGCTATGTGGCGCGGTATTATCCCGTATTGACGCCGGGCAAGAGCAACTCGGTCGCCGCAT
ACACTATTCTCAGAATGACTTGGTTGAGTACTCACCAGTCACAGAAAAGCATCTTACGGATGGCAT
GACAGTAAGAGAATTATGCAGTGCTGCCATAACCATGAGTGATAACACTGCGGCCAACTTACTTC
TGACAACGATCGGAGGACCGAAGGAGCTAACCGCTTTTTTGCACAACATGGGGGATCATGTAACT
CGCCTTGATCGTTGGGAACCGGAGCTGAATGAAGCCATACCAAACGACGAGCGTGACACCACGA
TGCCTGTAGTAATGGTAACAACGTTGCGCAAACTATTAACTGGCGAACTACTTACTCTAGCTTCCC
GGCAACAATTAATAGACTGGATGGAGGCGGATAAAGTTGCAGGACCACTTCTGCGCTCGGCCCT
TCCGGCTGGCTGGTTTATTGCTGATAAATCTGGAGCCGGTGAGCGTGGGTCTCGCGGTATCATT
GCAGCACTGGGGCCAGATGGTAAGCCCTCCCGTATCGTAGTTATCTACACGACGGGGAGTCAGG
CAACTATGGATGAACGAAATAGACAGATCGCTGAGATAGGTGCCTCACTGATTAAGCATTGGTAA
CTGTCAGACCAAGTTTACTCATATATACTTTAGATTGATTTAAAACTTCATTTTTAATTTAAAAGGAT
CTAGGTGAAGATCCTTTTTGATAATCTCATGACCAAAATCCCTTAACGTGAGTTTTCGTTCCACTG
AGCGTCAGACCCCGTAGAAAAGATCAAAGGATCTTCTTGAGATCCTTTTTTTCTGCGCGTAATCTG
CTGCTTGCAAACAAAAAAACCACCGCTACCAGCGGTGGTTTGTTTGCCGGATCAAGAGCTACCAA
CTCTTTTTCCGAAGGTAACTGGCTTCAGCAGAGCGCAGATACCAAATACTGTCCTTCTAGTGTAG
CCGTAGTTAGGCCACCACTTCAAGAACTCTGTAGCACCGCCTACATACCTCGCTCTGCTAATCCT
GTTACCAGTGGCTGCTGCCAGTGGCGATAAGTCGTGTCTTACCGGGTTGGACTCAAGACGATAG
TTACCGGATAAGGCGCAGCGGTCGGGCTGAACGGGGGGTTCGTGCACACAGCCCAGCTTGGAG
CGAACGACCTACACCGAACTGAGATACCTACAGCGTGAGCTATGAGAAAGCGCCACGCTTCCCG
AAGGGAGAAAGGCGGACAGGTATCCGGTAAGCGGCAGGGTCGGAACAGGAGAGCGCACGAGG
GAGCTTCCAGGGGGAAACGCCTGGTATCTTTATAGTCCTGTCGGGTTTCGCCACCTCTGACTTGA
GCGTCGATTTTTGTGATGCTCGTCAGGGGGGCGGAGCCTATGGAAAAACGCCAGCAACGCGGC
CTTTTTACGGTTCCTGGCCTTTTGCTGCGGTTTTGCTCACATGTTCTTTCCTGCGTTATCCCCTGA
TTCTGTGGATAACCGTATTACCGCCTTTGAGTGAGCTGATACCGCTCGCCGCAGCCGAACGACC
GAGCGCAGCGAGTCAGTGAGCGAGGAAGCGGAAGAGCGCCCAATACGCAAACCGCCTCTCCCC
GCGCGTTGGCCGATTCATTAATGCAGCTGGCACGACAGGTTTCCCGACTGGAAAGCGGGCAGTG
AGCGCAACGCAATTAATGTGAGTTAGCTCACTCATTAGGCACCCCAGGCTTTACACTTTATGCTTC
CGGCTCGTATGTTGTGTGGAATTGTGAGCGGATAACAATTTCACA

6. pAAV-sgRho-3xsgOpn1mw-RHO-5’Cas9-SDS-BD10-SV40pA (7413 bp)

CAGGAAACAGCTATGACCATGATTACGCCAGATTTAATTAAGGCTGCGCGCTCGCTCGCTCACTG
AGGCCGCCCGGGCAAAGCCCGGGCGTCGGGCGACCTTTGGTCGCCCGGCCTCAGTGAGCGAG

CGAGCGCGCAGAGAGGGAGTGGCCAACTCCATCACTAGGGGTTCCTTGTAGTTAATGATTAACC
CGCCATGCTACTTATCTACGTAGCCATGCTCTAGGAAGATCGGAATTCGCCCTTAAGGGCGCGC
CGTTTAAACGAGGGCCTATTTCCCATGATTCCTTCATATTTGCATATACGATACAAGGCTGTTAGA
GAGATAATTGGAATTAATTTGACTGTAAACACAAAGATATTAGTACAAAATACGTGACGTAGAAAG
TAATAATTTCTTGGGTAGTTTGCAGTTTTAAAATTATGTTTTAAAATGGACTATCATATGCTTACCGT
AACTTGAAAGTATTTCGATTTCTTGGCTTTATATATCTTGTGGAAAGGACGAAACACCGTACGGTG
ACGTAGAGCGTGGTTTTAGAGCTAGAAATAGCAAGTTAAAATAAGGCTAGTCCGTTATCAACTTGA
AAAAGTGGCACCGAGTCGGTGCTTTTTTTGTATACGAGGGCCTATTTCCCATGATTCCTTCATATT
TGCATATACGATACAAGGCTGTTAGAGAGATAATTGGAATTAATTTGACTGTAAACACAAAGATAT
TAGTACAAAATACGTGACGTAGAAAGTAATAATTTCTTGGGTAGTTTGCAGTTTTAAAATTATGTTT
TAAAATGGACTATCATATGCTTACCGTAACTTGAAAGTATTTCGATTTCTTGGCTTTATATATCTTG
TGGAAAGGACGAAACACCGGGGCCTTTAAGGTAGTTTTAGAGCTAGAAATAGCAAGTTAAAATAA
GGCTAGTCCGTTATCAACTTGAAAAAGTGGCACCGAGTCGGTGCTTTTTTTGTTAACGAGGGCCT
ATTTCCCATGATTCCTTCATATTTGCATATACGATACAAGGCTGTTAGAGAGATAATTGGAATTAAT
TTGACTGTAAACACAAAGATATTAGTACAAAATACGTGACGTAGAAAGTAATAATTTCTTGGGTAG
TTTGCAGTTTTAAAATTATGTTTTAAAATGGACTATCATATGCTTACCGTAACTTGAAAGTATTTCGA
TTTCTTGGCTTTATATATCTTGTGGAAAGGACGAAACACCGCCACCCCTGTGGATGTTTTAGAGCT
AGAAATAGCAAGTTAAAATAAGGCTAGTCCGTTATCAACTTGAAAAAGTGGCACCGAGTCGGTGC
TTTTTTTATTTAAATAGCTAGGAGGGCCTATTTCCCATGATTCCTTCATATTTGGCATATACGATAC
AAGGCTGTTAGAGAGATAATTGGAATTAATTTGACTGTAAACACAAAGATATTAGTACAAAATACG
TGACGTAGAAAGTAATAATTTCTTGGGTAGTTTGCAGTTTTAAAATTATGTTTTAAAATGGACTATC
ATATGCTTACCGTAACTTGAAAGTATTTCGATTTCTTGGCTTTATATATCTTGTGGAAAGGACGAAA
CAGCTTGCTTGTTTACAAGTTTTAGAGCTAGAAATAGCAAGTTAAAATAAGGCTAGTCCGTTATCA
ACTTGAAAAAGTGGCACCGAGTCGGTGCTTTTTTTCACGTGCTAGCCCTCTCCTCCCTGACCTCA
GGCTTCCTCCTAGTGTCACCTTGGCCCCTCTTAGAAGCCAATTAGGCCCTCAGTTTCTGCAGCGG
GGATTAATATGATTATGAACACCCCCAATCTCCCAGATGCTGATTCAGCCAGGAGCTTAGGAGGG
GGAGGTCACTTTATAAGGGTCTGGGGGGGTCAGAACCCAGAGTCATCGGTACCACCGGTCGCCA
CCATGGCCCCAAAGAAGAAGCGGAAGGTCGGTATCCACGGAGTCCCAGCAGCCGACAAGAAGT
ACTCCATTGGGCTCGATATCGGCACAAACAGCGTCGGCTGGGCCGTCATTACGGACGAGTACAA
GGTGCCGAGCAAAAAATTCAAAGTTCTGGGCAATACCGATCGCCACAGCATAAAGAAGAACCTCA
TTGGCGCCCTCCTGTTCGACTCCGGGGAAACGGCCGAAGCCACGCGGCTCAAAAGAACAGCAC
GGCGCAGATATACCCGCAGAAAGAATCGGATCTGCTACCTGCAGGAGATCTTTAGTAATGAGATG
GCTAAGGTGGATGACTCTTTCTTCCATAGGCTGGAGGAGTCCTTTTTGGTGGAGGAGGATAAAAA
GCACGAGCGCCACCCAATCTTTGGCAATATCGTGGACGAGGTGGCGTACCATGAAAAGTACCCA
ACCATATATCATCTGAGGAAGAAGCTTGTAGACAGTACTGATAAGGCTGACTTGCGGTTGATCTAT
CTCGCGCTGGCGCATATGATCAAATTTCGGGGACACTTCCTCATCGAGGGGGACCTGAACCCAG
ACAACAGCGATGTCGACAAACTCTTTATCCAACTGGTTCAGACTTACAATCAGCTTTTCGAAGAGA
ACCCGATCAACGCATCCGGAGTTGACGCCAAAGCAATCCTGAGCGCTAGGCTGTCCAAATCCCG
GCGGCTCGAAAACCTCATCGCACAGCTCCCTGGGGAGAAGAAGAACGGCCTGTTTGGTAATCTT
ATCGCCCTGTCACTCGGGCTGACCCCCAACTTTAAATCTAACTTCGACCTGGCCGAAGATGCCAA
GCTTCAACTGAGCAAAGACACCTACGATGATGATCTCGACAATCTGCTGGCCCAGATCGGCGAC
CAGTACGCAGACCTTTTTTTGGCGGCAAAGAACCTGTCAGACGCCATTCTGCTGAGTGATATTCT
GCGAGTGAACACGGAGATCACCAAAGCTCCGCTGAGCGCTAGTATGATCAAGCGCTATGATGAG
CACCACCAAGACTTGACTTTGCTGAAGGCCCTTGTCAGACAGCAACTGCCTGAGAAGTACAAGGA
AATTTTCTTCGATCAGTCTAAAAATGGCTACGCCGGATACATTGACGGCGGAGCAAGCCAGGAGG
AATTTTACAAATTTATTAAGCCCATCTTGGAAAAAATGGACGGCACCGAGGAGCTGCTGGTAAAG
CTTAACAGAGAAGATCTGTTGCGCAAACAGCGCACTTTCGACAATGGAAGCATCCCCCACCAGAT
TCACCTGGGCGAACTGCACGCTATCCTCAGGCGGCAAGAGGATTTCTACCCCTTTTTGAAAGATA
ACAGGGAAAAGATTGAGAAAATCCTCACATTTCGGATACCCTACTATGTAGGCCCCCTCGCCCGG
GGAAATTCCAGATTCGCGTGGATGACTCGCAAATCAGAAGAGACCATCACTCCCTGGAACTTCGA
GGAAGTCGTGGATAAGGGGGCCTCTGCCCAGTCCTTCATCGAAAGGATGACTAACTTTGATAAAA
ATCTGCCTAACGAAAAGGTGCTTCCTAAACACTCTCTGCTGTACGAGTACTTCACAGTTTATAACG
AGCTCACCAAGGTCAAATACGTCACAGAAGGGATGAGAAAGCCAGCATTCCTGTCTGGAGAGCA
GAAGAAAGCTATCGTGGACCTCCTCTTCAAGACGAACCGGAAAGTTACCGTGAAACAGCTCAAAG
AAGACTATTTCAAAAAGATTGAATGTTTCGACTCTGTTGAAATCAGCGGAGTGGAGGATCGCTTCA
ACGCATCCCTGGGAACGTATCACGATCTCCTGAAAATCATTAAAGACAAGGACTTCCTGGACAAT
GAGGAGAACGAGGACATTCTTGAGGACATTGTCCTCACCCTTACGTTGTTTGAAGATAGGGAGAT

GATTGAAGAACGCTTGAAAACTTACGCTCATCTCTTCGACGACAAAGTCATGAAACAGCTCAAGA
GGCGCCGATATACAGGATGGGGGCGGCTGTCAAGAAAACTGATCAATGGGATCCGAGACAAGCA
GAGTGGAAAGACAATCCTGGATTTTCTTAAGTCCGATGGATTTGCCAACCGGAACTTCATGCAGT
TGATCCATGATGACTCTCTCACCTTTAAGGAGGACATCCAGAAAGCACAAGTTTCTGGCCAGGGG
GACAGTCTTCACGAGCACATCGCTAATCTTGCAGGTAAGGGCACTGAGCAGAAGGGAAGAAGCT
CCGGGGGCTCTTTGTAGGGTGCGGCCGCAGTGCATCAAGGCGATCACATCAGTGAAAAAAAGCC
AGACAGGCGGTTAAACCAACGCAGATTAAACAGCAGGATGCAAAAATTCGCAGGTGGTCAGATG
GCGGCCGCTCTAGACTCGATGAGTTTGGACAAACCACAACTAGAATGCAGTGAAAAAAATGCTTT
ATTTGTGAAATTTGTGATGCTATTGCTTTATTTGTAACCATTATAAGCTGCAATAAACAAGTTCTCG
AGTTAAGGGCGAATTCCCGATTAGGATCTTCCTAGAGCATGGCTACGTAGATAAGTAGCATGGCG
GGTTAATCATTAACTACAAGGAACCCCTAGTGATGGAGTTGGCCACTCCCTCTCTGCGCGCTCGC
TCGCTCACTGAGGCCGGGCGACCAAAGGTCGCCCGACGCCCGGGCTTTGCCCGGGCGGCCTC
AGTGAGCGAGCGAGCGCGCAGCCTTAATTAACCTAATTCACTGGCCGTCGTTTTACAACGTCGTG
ACTGGGAAAACCCTGGCGTTACCCAACTTAATCGCCTTGCAGCACATCCCCCTTTCGCCAGCTGG
CGTAATAGCGAAGAGGCCCGCACCGATCGCCCTTCCCAACAGTTGCGCAGCCTGAATGGCGAAT
GGGACGCGCCCTGTAGCGGCGCATTAAGCGCGGCGGGTGTGGTGGTTACGCGCAGCGTGACC
GCTACACTTGCCAGCGCCCTAGCGCCCGCTCCTTTCGCTTTCTTCCCTTCCTTTCTCGCCACGTT
CGCCGGCTTTCCCCGTCAAGCTCTAAATCGGGGGCTCCCTTTAGGGTTCCGATTTAGTGCTTTAC
GGCACCTCGACCCCAAAAAACTTGATTAGGGTGATGGTTCACGTAGTGGGCCATCGCCCCGATA
GACGGTTTTTCGCCCTTTGACGCTGGAGTTCACGTTCCTCAATAGTGGACTCTTGTTCCAAACTG
GAACAACACTCAACCCTATCTCGGTCTATTCTTTTGATTTATAAGGGATTTTCCGATTTCGGCCTA
TTGGTTAAAAAATGAGCTGATTTAACAAAAATTTAACGCGAATTTTAACAAAATATTAACGTTTATAA
TTTCAGGTGGCATCTTTCGGGGAAATGTGCGCGGAACCCCTATTTGTTTATTTTTCTAAATACATT
CAAATATGTATCCGCTCATGAGACAATAACCCTGATAAATGCTTCAATAATATTGAAAAAGGAAGA
GTATGAGTATTCAACATTTCCGTGTCGCCCTTATTCCCTTTTTTGCGGCATTTTGCCTTCCTGTTTT
TGCTCACCCAGAAACGCTGGTGAAAGTAAAAGATGCTGAAGATCAGTTGGGTGCACGAGTGGGT
TACATCGAACTGGATCTCAATAGTGGTAAGATCCTTGAGAGTTTTCGCCCCGAAGAACGTTTTCCA
ATGATGAGCACTTTTAAAGTTCTGCTATGTGGCGCGGTATTATCCCGTATTGACGCCGGGCAAGA
GCAACTCGGTCGCCGCATACACTATTCTCAGAATGACTTGGTTGAGTACTCACCAGTCACAGAAA
AGCATCTTACGGATGGCATGACAGTAAGAGAATTATGCAGTGCTGCCATAACCATGAGTGATAAC
ACTGCGGCCAACTTACTTCTGACAACGATCGGAGGACCGAAGGAGCTAACCGCTTTTTTGCACAA
CATGGGGGATCATGTAACTCGCCTTGATCGTTGGGAACCGGAGCTGAATGAAGCCATACCAAAC
GACGAGCGTGACACCACGATGCCTGTAGTAATGGTAACAACGTTGCGCAAACTATTAACTGGCGA
ACTACTTACTCTAGCTTCCCGGCAACAATTAATAGACTGGATGGAGGCGGATAAAGTTGCAGGAC
CACTTCTGCGCTCGGCCCTTCCGGCTGGCTGGTTTATTGCTGATAAATCTGGAGCCGGTGAGCG
TGGGTCTCGCGGTATCATTGCAGCACTGGGGCCAGATGGTAAGCCCTCCCGTATCGTAGTTATC
TACACGACGGGGAGTCAGGCAACTATGGATGAACGAAATAGACAGATCGCTGAGATAGGTGCCT
CACTGATTAAGCATTGGTAACTGTCAGACCAAGTTTACTCATATATACTTTAGATTGATTTAAAACT
TCATTTTTAATTTAAAAGGATCTAGGTGAAGATCCTTTTTGATAATCTCATGACCAAAATCCCTTAA
CGTGAGTTTTCGTTCCACTGAGCGTCAGACCCCGTAGAAAAGATCAAAGGATCTTCTTGAGATCC
TTTTTTTCTGCGCGTAATCTGCTGCTTGCAAACAAAAAAACCACCGCTACCAGCGGTGGTTTGTTT
GCCGGATCAAGAGCTACCAACTCTTTTTCCGAAGGTAACTGGCTTCAGCAGAGCGCAGATACCAA
ATACTGTCCTTCTAGTGTAGCCGTAGTTAGGCCACCACTTCAAGAACTCTGTAGCACCGCCTACA
TACCTCGCTCTGCTAATCCTGTTACCAGTGGCTGCTGCCAGTGGCGATAAGTCGTGTCTTACCGG
GTTGGACTCAAGACGATAGTTACCGGATAAGGCGCAGCGGTCGGGCTGAACGGGGGGTTCGTG
CACACAGCCCAGCTTGGAGCGAACGACCTACACCGAACTGAGATACCTACAGCGTGAGCTATGA
GAAAGCGCCACGCTTCCCGAAGGGAGAAAGGCGGACAGGTATCCGGTAAGCGGCAGGGTCGGA
ACAGGAGAGCGCACGAGGGAGCTTCCAGGGGGAAACGCCTGGTATCTTTATAGTCCTGTCGGGT
TTCGCCACCTCTGACTTGAGCGTCGATTTTTGTGATGCTCGTCAGGGGGGCGGAGCCTATGGAA
AAACGCCAGCAACGCGGCCTTTTTACGGTTCCTGGCCTTTTGCTGCGGTTTTGCTCACATGTTCT
TTCCTGCGTTATCCCCTGATTCTGTGGATAACCGTATTACCGCCTTTGAGTGAGCTGATACCGCT
CGCCGCAGCCGAACGACCGAGCGCAGCGAGTCAGTGAGCGAGGAAGCGGAAGAGCGCCCAAT
ACGCAAACCGCCTCTCCCCGCGCGTTGGCCGATTCATTAATGCAGCTGGCACGACAGGTTTCCC
GACTGGAAAGCGGGCAGTGAGCGCAACGCAATTAATGTGAGTTAGCTCACTCATTAGGCACCCC
AGGCTTTACACTTTATGCTTCCGGCTCGTATGTTGTGTGGAATTGTGAGCGGATAACAATTTCACA

7. **pAAV-sgRho-2xsgOpn1mw-RHO-Cas9N-RmaIntN-SV40pA (7154 bp)**

CAGGAAACAGCTATGACCATGATTACGCCAGATTTAATTAAGGCTGCGCGCTCGCTCGCTCACTG
AGGCCGCCCGGGCAAAGCCCGGGCGTCGGGCGACCTTTGGTCGCCCGGCCTCAGTGAGCGAG
CGAGCGCGCAGAGAGGGAGTGGCCAACTCCATCACTAGGGGTTCCTTGTAGTTAATGATTAACC
CGCCATGCTACTTATCTACGTAGCCATGCTCTAGGAAGATCGGAATTCGCCCTTAAGGGCGCGC
CGTTTAAACGAGGGCCTATTTCCCATGATTCCTTCATATTTGCATATACGATACAAGGCTGTTAGA
GAGATAATTGGAATTAATTTGACTGTAAACACAAAGATATTAGTACAAAATACGTGACGTAGAAAG
TAATAATTTCTTGGGTAGTTTGCAGTTTTAAAATTATGTTTTAAAATGGACTATCATATGCTTACCGT
AACTTGAAAGTATTTCGATTTCTTGGCTTTATATATCTTGTGGAAAGGACGAAACACCGTACGGTG
ACGTAGAGCGTGGTTTTAGAGCTAGAAATAGCAAGTTAAAATAAGGCTAGTCCGTTATCAACTTGA
AAAAGTGGCACCGAGTCGGTGCTTTTTTTGTATACGAGGGCCTATTTCCCATGATTCCTTCATATT
TGCATATACGATACAAGGCTGTTAGAGAGATAATTGGAATTAATTTGACTGTAAACACAAAGATAT
TAGTACAAAATACGTGACGTAGAAAGTAATAATTTCTTGGGTAGTTTGCAGTTTTAAAATTATGTTT
TAAAATGGACTATCATATGCTTACCGTAACTTGAAAGTATTTCGATTTCTTGGCTTTATATATCTTG
TGGAAAGGACGAAACACCGGGGCCTTTAAGGTAGTTTTAGAGCTAGAAATAGCAAGTTAAAATAA
GGCTAGTCCGTTATCAACTTGAAAAAGTGGCACCGAGTCGGTGCTTTTTTTGTTAACGAGGGCCT
ATTTCCCATGATTCCTTCATATTTGCATATACGATACAAGGCTGTTAGAGAGATAATTGGAATTAAT
TTGACTGTAAACACAAAGATATTAGTACAAAATACGTGACGTAGAAAGTAATAATTTCTTGGGTAG
TTTGCAGTTTTAAAATTATGTTTTAAAATGGACTATCATATGCTTACCGTAACTTGAAAGTATTTCGA
TTTCTTGGCTTTATATATCTTGTGGAAAGGACGAAACACCGCCACCCCTGTGGATGTTTTAGAGCT
AGAAATAGCAAGTTAAAATAAGGCTAGTCCGTTATCAACTTGAAAAAGTGGCACCGAGTCGGTGC
TTTTTTTATTTAAATAGCGAGCCCTCTCCTCCCTGACCTCAGGCTTCCTCCTAGTGTCACCTTGGC
CCCTCTTAGAAGCCAATTAGGCCCTCAGTTTCTGCAGCGGGGATTAATATGATTATGAACACCCC
CAATCTCCCAGATGCTGATTCAGCCAGGAGCTTAGGAGGGGGAGGTCACTTTATAAGGGTCTGG
GGGGGTCAGAACCCAGAGTCATCGGTACCACCGGTCGCCACCATGGCCCCAAAGAAGAAGCGG
AAGGTCGGTATCCACGGAGTCCCAGCAGCCGACAAGAAGTACTCCATTGGGCTCGATATCGGCA
CAAACAGCGTCGGCTGGGCCGTCATTACGGACGAGTACAAGGTGCCGAGCAAAAAATTCAAAGT
TCTGGGCAATACCGATCGCCACAGCATAAAGAAGAACCTCATTGGCGCCCTCCTGTTCGACTCC
GGGGAAACGGCCGAAGCCACGCGGCTCAAAAGAACAGCACGGCGCAGATATACCCGCAGAAAG
AATCGGATCTGCTACCTGCAGGAGATCTTTAGTAATGAGATGGCTAAGGTGGATGACTCTTTCTT
CCATAGGCTGGAGGAGTCCTTTTTGGTGGAGGAGGATAAAAAGCACGAGCGCCACCCAATCTTT
GGCAATATCGTGGACGAGGTGGCGTACCATGAAAAGTACCCAACCATATATCATCTGAGGAAGAA
GCTTGTAGACAGTACTGATAAGGCTGACTTGCGGTTGATCTATCTCGCGCTGGCGCATATGATCA
AATTTCGGGGACACTTCCTCATCGAGGGGGACCTGAACCCAGACAACAGCGATGTCGACAAACT
CTTTATCCAACTGGTTCAGACTTACAATCAGCTTTTCGAAGAGAACCCGATCAACGCATCCGGAG
TTGACGCCAAAGCAATCCTGAGCGCTAGGCTGTCCAAATCCCGGCGGCTCGAAAACCTCATCGC
ACAGCTCCCTGGGGAGAAGAAGAACGGCCTGTTTGGTAATCTTATCGCCCTGTCACTCGGGCTG
ACCCCCAACTTTAAATCTAACTTCGACCTGGCCGAAGATGCCAAGCTTCAACTGAGCAAAGACAC
CTACGATGATGATCTCGACAATCTGCTGGCCCAGATCGGCGACCAGTACGCAGACCTTTTTTTGG
CGGCAAAGAACCTGTCAGACGCCATTCTGCTGAGTGATATTCTGCGAGTGAACACGGAGATCAC
CAAAGCTCCGCTGAGCGCTAGTATGATCAAGCGCTATGATGAGCACCACCAAGACTTGACTTTGC
TGAAGGCCCTTGTCAGACAGCAACTGCCTGAGAAGTACAAGGAAATTTTCTTCGATCAGTCTAAA
AATGGCTACGCCGGATACATTGACGGCGGAGCAAGCCAGGAGGAATTTTACAAATTTATTAAGCC
CATCTTGGAAAAAATGGACGGCACCGAGGAGCTGCTGGTAAAGCTTAACAGAGAAGATCTGTTG
CGCAAACAGCGCACTTTCGACAATGGAAGCATCCCCCACCAGATTCACCTGGGCGAACTGCACG
CTATCCTCAGGCGGCAAGAGGATTTCTACCCCTTTTTGAAAGATAACAGGGAAAAGATTGAGAAA
ATCCTCACATTTCGGATACCCTACTATGTAGGCCCCCTCGCCCGGGGAAATTCCAGATTCGCGTG
GATGACTCGCAAATCAGAAGAGACCATCACTCCCTGGAACTTCGAGGAAGTCGTGGATAAGGGG
GCCTCTGCCCAGTCCTTCATCGAAAGGATGACTAACTTTGATAAAAATCTGCCTAACGAAAAGGT
GCTTCCTAAACACTCTCTGCTGTACGAGTACTTCACAGTTTATAACGAGCTCACCAAGGTCAAATA
CGTCACAGAAGGGATGAGAAAGCCAGCATTCCTGTCTGGAGAGCAGAAGAAAGCTATCGTGGAC
CTCCTCTTCAAGACGAACCGGAAAGTTACCGTGAAACAGCTCAAAGAAGACTATTTCAAAAAGATT
GAATGTTTCGACTCTGTTGAAATCAGCGGAGTGGAGGATCGCTTCAACGCATCCCTGGGAACGTA
TCACGATCTCCTGAAAATCATTAAAGACAAGGACTTCCTGGACAATGAGGAGAACGAGGACATTC
TTGAGGACATTGTCCTCACCCTTACGTTGTTTGAAGATAGGGAGATGATTGAAGAACGCTTGAAA
ACTTACGCTCATCTCTTCGACGACAAAGTCATGAAACAGCTCAAGAGGCGCCGATATACAGGATG
GGGGCGGCTGTCAAGAAAACTGATCAATGGGATCCGAGACAAGCAGAGTGGAAAGACAATCCTG

GATTTTCTTAAGTCCGATGGATTTGCCAACCGGAACTTCATGCAGTTGATCCATGATGACTCTCTC
ACCTTTAAGGAGGACATCCAGAAAGCACAAGTTTGTCTGGCTGGCGATACTCTCATTACCCTGGC
CGATGGACGACGAGTGCCTATTAGAGAACTGGTGTCACAGCAGAATTTTTCCGTGTGGGCTCTGA
ATCCTCAGACTTACCGCCTGGAGAGGGCTAGAGTGAGTAGAGCTTTCTGTACCGGCATCAAACCT
GTGTACCGCCTCACCACTAGACTGGGGAGATCCATTAGGGCCACTGCCAACCACCGATTTCTCA
CACCTCAGGGCTGGAAACGAGTCGATGAACTCCAGCCTGGAGATTACCTGGCTCTGCCTAGGAG
AATCCCTACTGCCTCCTGATCTCGAGCTCGATGAGTTTGGACAAACCACAACTAGAATGCAGTGA
AAAAAATGCTTTATTTGTGAAATTTGTGATGCTATTGCTTTATTTGTAACCATTATAAGCTGCAATAA
ACAAGTTCTCGAGTTAAGGGCGAATTCCCGATTAGGATCTTCCTAGAGCATGGCTACGTAGATAA
GTAGCATGGCGGGTTAATCATTAACTACAAGGAACCCCTAGTGATGGAGTTGGCCACTCCCTCTC
TGCGCGCTCGCTCGCTCACTGAGGCCGGGCGACCAAAGGTCGCCCGACGCCCGGGCTTTGCCC
GGGCGGCCTCAGTGAGCGAGCGAGCGCGCAGCCTTAATTAACCTAATTCACTGGCCGTCGTTTT
ACAACGTCGTGACTGGGAAAACCCTGGCGTTACCCAACTTAATCGCCTTGCAGCACATCCCCCTT
TCGCCAGCTGGCGTAATAGCGAAGAGGCCCGCACCGATCGCCCTTCCCAACAGTTGCGCAGCCT
GAATGGCGAATGGGACGCGCCCTGTAGCGGCGCATTAAGCGCGGCGGGTGTGGTGGTTACGCG
CAGCGTGACCGCTACACTTGCCAGCGCCCTAGCGCCCGCTCCTTTCGCTTTCTTCCCTTCCTTTC
TCGCCACGTTCGCCGGCTTTCCCCGTCAAGCTCTAAATCGGGGGCTCCCTTTAGGGTTCCGATTT
AGTGCTTTACGGCACCTCGACCCCAAAAAACTTGATTAGGGTGATGGTTCACGTAGTGGGCCATC
GCCCCGATAGACGGTTTTTCGCCCTTTGACGCTGGAGTTCACGTTCCTCAATAGTGGACTCTTGT
TCCAAACTGGAACAACACTCAACCCTATCTCGGTCTATTCTTTTGATTTATAAGGGATTTTCCGAT
TTCGGCCTATTGGTTAAAAAATGAGCTGATTTAACAAAAATTTAACGCGAATTTTAACAAAATATTA
ACGTTTATAATTTCAGGTGGCATCTTTCGGGGAAATGTGCGCGGAACCCCTATTTGTTTATTTTTC
TAAATACATTCAAATATGTATCCGCTCATGAGACAATAACCCTGATAAATGCTTCAATAATATTGAA
AAAGGAAGAGTATGAGTATTCAACATTTCCGTGTCGCCCTTATTCCCTTTTTTGCGGCATTTTGCC
TTCCTGTTTTTGCTCACCCAGAAACGCTGGTGAAAGTAAAAGATGCTGAAGATCAGTTGGGTGCA
CGAGTGGGTTACATCGAACTGGATCTCAATAGTGGTAAGATCCTTGAGAGTTTTCGCCCCGAAGA
ACGTTTTCCAATGATGAGCACTTTTAAAGTTCTGCTATGTGGCGCGGTATTATCCCGTATTGACGC
CGGGCAAGAGCAACTCGGTCGCCGCATACACTATTCTCAGAATGACTTGGTTGAGTACTCACCAG
TCACAGAAAAGCATCTTACGGATGGCATGACAGTAAGAGAATTATGCAGTGCTGCCATAACCATG
AGTGATAACACTGCGGCCAACTTACTTCTGACAACGATCGGAGGACCGAAGGAGCTAACCGCTTT
TTTGCACAACATGGGGGATCATGTAACTCGCCTTGATCGTTGGGAACCGGAGCTGAATGAAGCC
ATACCAAACGACGAGCGTGACACCACGATGCCTGTAGTAATGGTAACAACGTTGCGCAAACTATT
AACTGGCGAACTACTTACTCTAGCTTCCCGGCAACAATTAATAGACTGGATGGAGGCGGATAAAG
TTGCAGGACCACTTCTGCGCTCGGCCCTTCCGGCTGGCTGGTTTATTGCTGATAAATCTGGAGCC
GGTGAGCGTGGGTCTCGCGGTATCATTGCAGCACTGGGGCCAGATGGTAAGCCCTCCCGTATC
GTAGTTATCTACACGACGGGGAGTCAGGCAACTATGGATGAACGAAATAGACAGATCGCTGAGAT
AGGTGCCTCACTGATTAAGCATTGGTAACTGTCAGACCAAGTTTACTCATATATACTTTAGATTGA
TTTAAAACTTCATTTTTAATTTAAAAGGATCTAGGTGAAGATCCTTTTTGATAATCTCATGACCAAAA
TCCCTTAACGTGAGTTTTCGTTCCACTGAGCGTCAGACCCCGTAGAAAAGATCAAAGGATCTTCTT
GAGATCCTTTTTTTCTGCGCGTAATCTGCTGCTTGCAAACAAAAAAACCACCGCTACCAGCGGTG
GTTTGTTTGCCGGATCAAGAGCTACCAACTCTTTTTCCGAAGGTAACTGGCTTCAGCAGAGCGCA
GATACCAAATACTGTCCTTCTAGTGTAGCCGTAGTTAGGCCACCACTTCAAGAACTCTGTAGCAC
CGCCTACATACCTCGCTCTGCTAATCCTGTTACCAGTGGCTGCTGCCAGTGGCGATAAGTCGTGT
CTTACCGGGTTGGACTCAAGACGATAGTTACCGGATAAGGCGCAGCGGTCGGGCTGAACGGGG
GGTTCGTGCACACAGCCCAGCTTGGAGCGAACGACCTACACCGAACTGAGATACCTACAGCGTG
AGCTATGAGAAAGCGCCACGCTTCCCGAAGGGAGAAAGGCGGACAGGTATCCGGTAAGCGGCA
GGGTCGGAACAGGAGAGCGCACGAGGGAGCTTCCAGGGGGAAACGCCTGGTATCTTTATAGTC
CTGTCGGGTTTCGCCACCTCTGACTTGAGCGTCGATTTTTGTGATGCTCGTCAGGGGGGCGGAG
CCTATGGAAAAACGCCAGCAACGCGGCCTTTTTACGGTTCCTGGCCTTTTGCTGCGGTTTTGCTC
ACATGTTCTTTCCTGCGTTATCCCCTGATTCTGTGGATAACCGTATTACCGCCTTTGAGTGAGCTG
ATACCGCTCGCCGCAGCCGAACGACCGAGCGCAGCGAGTCAGTGAGCGAGGAAGCGGAAGAG
CGCCCAATACGCAAACCGCCTCTCCCCGCGCGTTGGCCGATTCATTAATGCAGCTGGCACGACA
GGTTTCCCGACTGGAAAGCGGGCAGTGAGCGCAACGCAATTAATGTGAGTTAGCTCACTCATTA
GGCACCCCAGGCTTTACACTTTATGCTTCCGGCTCGTATGTTGTGTGGAATTGTGAGCGGATAAC
AATTTCACA

8. pAAV-RHO-RmaIntC-Cas9C-VPR-synpA (7343 bp)

CAGGAAACAGCTATGACCATGATTACGCCAGATTTAATTAAGGCTGCGCGCTCGCTCGCTCACTG
AGGCCGCCCGGGCAAAGCCCGGGCGTCGGGCGACCTTTGGTCGCCCGGCCTCAGTGAGCGAG
CGAGCGCGCAGAGAGGGAGTGGCCAACTCCATCACTAGGGGTTCCTTGTAGTTAATGATTAACC
CGCCATGCTACTTATCTACGTAGCCATGCTCTAGGAAGATCGGAATTCGCCCTTAAGGGCGCGC
CGTTTAAATAGCTAGCCCTCTCCTCCCTGACCTCAGGCTTCCTCCTAGTGTCACCTTGGCCCCTC
TTAGAAGCCAATTAGGCCCTCAGTTTCTGCAGCGGGGATTAATATGATTATGAACACCCCCAATCT
CCCAGATGCTGATTCAGCCAGGAGCTTAGGAGGGGGAGGTCACTTTATAAGGGTCTGGGGGGG
TCAGAACCCAGAGTCATCGGTACCACCGGTCGACTAGAGGATCCATGGCGGCGGCGTGCCCGG
AACTGCGTCAGCTGGCGCAGAGCGATGTGTATTGGGATCCGATTGTGAGCATTGAACCGGATGG
CGTGGAAGAAGTGTTTGATCTGACCGTGCCGGGCCCGCATAACTTTGTGGCGAACGATATTATTG
CGCATAACTCTGGCCAGGGGGACAGTCTTCACGAGCACATCGCTAATCTTGCAGGTAGCCCAGC
TATCAAAAAGGGAATACTGCAGACCGTTAAGGTCGTGGATGAACTCGTCAAAGTAATGGGAAGGC
ATAAGCCCGAGAATATCGTTATCGAGATGGCCCGAGAGAACCAAACTACCCAGAAGGGACAGAA
GAACAGTAGGGAAAGGATGAAGAGGATTGAAGAGGGTATAAAAGAACTGGGGTCCCAAATCCTT
AAGGAACACCCAGTTGAAAACACCCAGCTTCAGAATGAGAAGCTCTACCTGTACTACCTGCAGAA
CGGCAGGGACATGTACGTGGATCAGGAACTGGACATCAATCGGCTCTCCGACTACGACGTGGAT
CATATCGTGCCCCAGTCTTTTCTCAAAGATGATTCTATTGATAATAAAGTGTTGACAAGATCCGAT
AAAAATAGAGGGAAGAGTGATAACGTCCCCTCAGAAGAAGTTGTCAAGAAAATGAAAAATTATTG
GCGGCAGCTGCTGAACGCCAAACTGATCACACAACGGAAGTTCGATAATCTGACTAAGGCTGAA
CGAGGTGGCCTGTCTGAGTTGGATAAAGCCGGCTTCATCAAAAGGCAGCTTGTTGAGACACGCC
AGATCACCAAGCACGTGGCCCAAATTCTCGATTCACGCATGAACACCAAGTACGATGAAAATGAC
AAACTGATTCGAGAGGTGAAAGTTATTACTCTGAAGTCTAAGCTGGTCTCAGATTTCAGAAAGGAC
TTTCAGTTTTATAAGGTGAGAGAGATCAACAATTACCACCATGCGCATGATGCCTACCTGAATGCA
GTGGTAGGCACTGCACTTATCAAAAAATATCCCAAGCTTGAATCTGAATTTGTTTACGGAGACTAT
AAAGTGTACGATGTTAGGAAAATGATCGCAAAGTCTGAGCAGGAAATAGGCAAGGCCACCGCTAA
GTACTTCTTTTACAGCAATATTATGAATTTTTTCAAGACCGAGATTACACTGGCCAATGGAGAGATT
CGGAAGCGACCACTTATCGAAACAAACGGAGAAACAGGAGAAATCGTGTGGGACAAGGGTAGGG
ATTTCGCGACAGTCCGGAAGGTCCTGTCCATGCCGCAGGTGAACATCGTTAAAAAGACCGAAGT
ACAGACCGGAGGCTTCTCCAAGGAAAGTATCCTCCCGAAAAGGAACAGCGACAAGCTGATCGCA
CGCAAAAAAGATTGGGACCCCAAGAAATACGGCGGATTCGATTCTCCTACAGTCGCTTACAGTGT
ACTGGTTGTGGCCAAAGTGGAGAAAGGGAAGTCTAAAAAACTCAAAAGCGTCAAGGAACTGCTG
GGCATCACAATCATGGAGCGATCAAGCTTCGAAAAAAACCCCATCGACTTTCTCGAGGCGAAAGG
ATATAAAGAGGTCAAAAAAGACCTCATCATTAAGCTTCCCAAGTACTCTCTCTTTGAGCTTGAAAA
CGGCCGGAAACGAATGCTCGCTAGTGCGGGCGAGCTGCAGAAAGGTAACGAGCTGGCACTGCC
CTCTAAATACGTTAATTTCTTGTATCTGGCCAGCCACTATGAAAAGCTCAAAGGGTCTCCCGAAGA
TAATGAGCAGAAGCAGCTGTTCGTGGAACAACACAAACACTACCTTGATGAGATCATCGAGCAAA
TAAGCGAATTCTCCAAAAGAGTGATCCTCGCCGACGCTAACCTCGATAAGGTGCTTTCTGCTTAC
AATAAGCACAGGGATAAGCCCATCAGGGAGCAGGCAGAAAACATTATCCACTTGTTTACTCTGAC
CAACTTGGGCGCGCCTGCAGCCTTCAAGTACTTCGACACCACCATAGACAGAAAGCGGTACACC
TCTACAAAGGAGGTCCTGGACGCCACACTGATTCATCAGTCAATTACGGGGCTCTATGAAACAAG
AATCGACCTCTCTCAGCTCGGTGGAGACAGCAGGGCTGACCCCAAGAAGAAGAGGAAGGTGTC
GCCAGGGATCCGTCGACTTGACGCGTTGATATCAACAAGTTTGTACAAAAAAGCAGGCTACAAAG
AGGCCAGCGGTTCCGGACGGGCTGACGCATTGGACGATTTTGATCTGGATATGCTGGGAAGTGA
CGCCCTCGATGATTTTGACCTTGACATGCTTGGTTCGGATGCCCTTGATGACTTTGACCTCGACA
TGCTCGGCAGTGACGCCCTTGATGATTTCGACCTGGACATGCTGATTAACTCTAGAAGTTCCGGA
TCTCCGAAAAAGAAACGCAAAGTTGGTAGCCAGTACCTGCCCGACACCGACGACCGGCACCGGA
TCGAGGAAAAGCGGAAGCGGACCTACGAGACATTCAAGAGCATCATGAAGAAGTCCCCCTTCAG
CGGCCCCACCGACCCTAGACCTCCACCTAGAAGAATCGCCGTGCCCAGCAGATCCAGCGCCAG
CGTGCCAAAACCTGCCCCCCAGCCTTACCCCTTCACCAGCAGCCTGAGCACCATCAACTACGAC
GAGTTCCCTACCATGGTGTTCCCCAGCGGCCAGATCTCTCAGGCCTCTGCTCTGGCTCCAGCCC
CTCCTCAGGTGCTGCCTCAGGCTCCTGCTCCTGCACCAGCTCCAGCCATGGTGTCTGCACTGGC
TCAGGCACCAGCACCCGTGCCTGTGCTGGCTCCTGGACCTCCACAGGCTGTGGCTCCACCAGC
CCCTAAACCTACACAGGCCGGCGAGGGCACACTGTCTGAAGCTCTGCTGCAGCTGCAGTTCGAC
GACGAGGATCTGGGAGCCCTGCTGGGAAACAGCACCGATCCTGCCGTGTTCACCGACCTGGCC
AGCGTGGACAACAGCGAGTTCCAGCAGCTGCTGAACCAGGGCATCCCTGTGGCCCCTCACACCA
CCGAGCCCATGCTGATGGAATACCCCGAGGCCATCACCCGGCTCGTGACAGGCGCTCAGAGGC

CTCCTGATCCAGCTCCTGCCCCTCTGGGAGCACCAGGCCTGCCTAATGGACTGCTGTCTGGCGA
CGAGGACTTCAGCTCTATCGCCGATATGGATTTCTCAGCCTTGCTGGGCTCTGGCAGCGGCAGC
CGGGATTCCAGGGAAGGGATGTTTTTGCCGAAGCCTGAGGCCGGCTCCGCTATTAGTGACGTGT
TTGAGGGCCGCGAGGTGTGCCAGCCAAAACGAATCCGGCCATTTCATCCTCCAGGAAGTCCATG
GGCCAACCGCCCACTCCCCGCCAGCCTCGCACCAACACCAACCGGTCCAGTACATGAGCCAGT
CGGGTCACTGACCCCGGCACCAGTCCCTCAGCCACTGGATCCAGCGCCCGCAGTGACTCCCGA
GGCCAGTCACCTGTTGGAGGATCCCGATGAAGAGACGAGCCAGGCTGTCAAAGCCCTTCGGGA
GATGGCCGATACTGTGATTCCCCAGAAGGAAGAGGCTGCAATCTGTGGCCAAATGGACCTTTCC
CATCCGCCCCCAAGGGGCCATCTGGATGAGCTGACAACCACACTTGAGTCCATGACCGAGGATC
TGAACCTGGACTCACCCCTGACCCCGGAATTGAACGAGATTCTGGATACCTTCCTGAACGACGA
GTGCCTCTTGCATGCCATGCATATCAGCACAGGACTGTCCATCTTCGACACATCTCTGTTTTGACA
ATAAAATATCTTTATTTTCATTACATCTGTGTGTTGGTTTTTTGTGTCTCGAGTTAAGGGCGAATTC
CCGATTAGGATCTTCCTAGAGCATGGCTACGTAGATAAGTAGCATGGCGGGTTAATCATTAACTA
CAAGGAACCCCTAGTGATGGAGTTGGCCACTCCCTCTCTGCGCGCTCGCTCGCTCACTGAGGCC
GGGCGACCAAAGGTCGCCCGACGCCCGGGCTTTGCCCGGGCGGCCTCAGTGAGCGAGCGAGC
GCGCAGCCTTAATTAACCTAATTCACTGGCCGTCGTTTTACAACGTCGTGACTGGGAAAACCCTG
GCGTTACCCAACTTAATCGCCTTGCAGCACATCCCCCTTTCGCCAGCTGGCGTAATAGCGAAGAG
GCCCGCACCGATCGCCCTTCCCAACAGTTGCGCAGCCTGAATGGCGAATGGGACGCGCCCTGT
AGCGGCGCATTAAGCGCGGCGGGTGTGGTGGTTACGCGCAGCGTGACCGCTACACTTGCCAGC
GCCCTAGCGCCCGCTCCTTTCGCTTTCTTCCCTTCCTTTCTCGCCACGTTCGCCGGCTTTCCCCG
TCAAGCTCTAAATCGGGGGCTCCCTTTAGGGTTCCGATTTAGTGCTTTACGGCACCTCGACCCCA
AAAAACTTGATTAGGGTGATGGTTCACGTAGTGGGCCATCGCCCCGATAGACGGTTTTTCGCCCT
TTGACGCTGGAGTTCACGTTCCTCAATAGTGGACTCTTGTTCCAAACTGGAACAACACTCAACCC
TATCTCGGTCTATTCTTTTGATTTATAAGGGATTTTTCCGATTTCGGCCTATTGGTTAAAAAATGAG
CTGATTTAACAAAAATTTAACGCGAATTTTAACAAAATATTAACGTTTATAATTTCAGGTGGCATCTT
TCGGGGAAATGTGCGCGGAACCCCTATTTGTTTATTTTTCTAAATACATTCAAATATGTATCCGCT
CATGAGACAATAACCCTGATAAATGCTTCAATAATATTGAAAAAGGAAGAGTATGAGTATTCAACA
TTTCCGTGTCGCCCTTATTCCCTTTTTTGCGGCATTTTGCCTTCCTGTTTTTGCTCACCCAGAAAC
GCTGGTGAAAGTAAAAGATGCTGAAGATCAGTTGGGTGCACGAGTGGGTTACATCGAACTGGAT
CTCAATAGTGGTAAGATCCTTGAGAGTTTTCGCCCCGAAGAACGTTTTCCAATGATGAGCACTTTT
AAAGTTCTGCTATGTGGCGCGGTATTATCCCGTATTGACGCCGGGCAAGAGCAACTCGGTCGCC
GCATACACTATTCTCAGAATGACTTGGTTGAGTACTCACCAGTCACAGAAAAGCATCTTACGGATG
GCATGACAGTAAGAGAATTATGCAGTGCTGCCATAACCATGAGTGATAACACTGCGGCCAACTTA
CTTCTGACAACGATCGGAGGACCGAAGGAGCTAACCGCTTTTTTGCACAACATGGGGGATCATGT
AACTCGCCTTGATCGTTGGGAACCGGAGCTGAATGAAGCCATACCAAACGACGAGCGTGACACC
ACGATGCCTGTAGTAATGGTAACAACGTTGCGCAAACTATTAACTGGCGAACTACTTACTCTAGCT
TCCCGGCAACAATTAATAGACTGGATGGAGGCGGATAAAGTTGCAGGACCACTTCTGCGCTCGG
CCCTTCCGGCTGGCTGGTTTATTGCTGATAAATCTGGAGCCGGTGAGCGTGGGTCTCGCGGTAT
CATTGCAGCACTGGGGCCAGATGGTAAGCCCTCCCGTATCGTAGTTATCTACACGACGGGGAGT
CAGGCAACTATGGATGAACGAAATAGACAGATCGCTGAGATAGGTGCCTCACTGATTAAGCATTG
GTAACTGTCAGACCAAGTTTACTCATATATACTTTAGATTGATTTAAAACTTCATTTTTAATTTAAAA
GGATCTAGGTGAAGATCCTTTTTGATAATCTCATGACCAAAATCCCTTAACGTGAGTTTTCGTTCC
ACTGAGCGTCAGACCCCGTAGAAAAGATCAAAGGATCTTCTTGAGATCCTTTTTTTCTGCGCGTAA
TCTGCTGCTTGCAAACAAAAAAACCACCGCTACCAGCGGTGGTTTGTTTGCCGGATCAAGAGCTA
CCAACTCTTTTTCCGAAGGTAACTGGCTTCAGCAGAGCGCAGATACCAAATACTGTCCTTCTAGT
GTAGCCGTAGTTAGGCCACCACTTCAAGAACTCTGTAGCACCGCCTACATACCTCGCTCTGCTAA
TCCTGTTACCAGTGGCTGCTGCCAGTGGCGATAAGTCGTGTCTTACCGGGTTGGACTCAAGACG
ATAGTTACCGGATAAGGCGCAGCGGTCGGGCTGAACGGGGGGTTCGTGCACACAGCCCAGCTT
GGAGCGAACGACCTACACCGAACTGAGATACCTACAGCGTGAGCTATGAGAAAGCGCCACGCTT
CCCGAAGGGAGAAAGGCGGACAGGTATCCGGTAAGCGGCAGGGTCGGAACAGGAGAGCGCAC
GAGGGAGCTTCCAGGGGGAAACGCCTGGTATCTTTATAGTCCTGTCGGGTTTCGCCACCTCTGA
CTTGAGCGTCGATTTTTGTGATGCTCGTCAGGGGGGCGGAGCCTATGGAAAAACGCCAGCAACG
CGGCCTTTTTACGGTTCCTGGCCTTTTGCTGCGGTTTTGCTCACATGTTCTTTCCTGCGTTATCCC
CTGATTCTGTGGATAACCGTATTACCGCCTTTGAGTGAGCTGATACCGCTCGCCGCAGCCGAAC
GACCGAGCGCAGCGAGTCAGTGAGCGAGGAAGCGGAAGAGCGCCCAATACGCAAACCGCCTCT
CCCCGCGCGTTGGCCGATTCATTAATGCAGCTGGCACGACAGGTTTCCCGACTGGAAAGCGGGC

AGTGAGCGCAACGCAATTAATGTGAGTTAGCTCACTCATTAGGCACCCCAGGCTTTACACTTTAT
GCTTCCGGCTCGTATGTTGTGTGGAATTGTGAGCGGATAACAATTTCACA

9. pGL2-RHO-5'ABCA4-SDS-BD10-SV40pA (9742 bp)

TTGGCCACTCCCTCTCTGCGCGCTCGCTCGCTCACTGAGGCCGCCCGGGCAAAGCCCGGGCGT
CGGGCGACCTTTGGTCGCCCGGCCTCAGTGAGCGAGCGAGCGCGCAGAGAGGGAGTGGCCAA
CTCCATCACTAGGGGTTCCTTGTAGTTAATGATTAACCCGCCATGCTACTTATCTACGTAGCCATG
CTCTAGGAAGATCGGAATTCGCCCTTAAGGGCGCGCCGTTTAAATAGCTAGCCCTCTCCTCCCTG
ACCTCAGGCTTCCTCCTAGTGTCACCTTGGCCCCTCTTAGAAGCCAATTAGGCCCTCAGTTTCTG
CAGCGGGGATTAATATGATTATGAACACCCCCAATCTCCCAGATGCTGATTCAGCCAGGAGCTTA
GGAGGGGGAGGTCACTTTATAAGGGTCTGGGGGGGTCAGAACCCAGAGTCATCGGTACCACCG
GTGCCACCATGGGCTTCGTGAGACAGATACAGCTTTTGCTCTGGAAGAACTGGACCCTGCGGAA
AAGGCAAAAGATTCGCTTTGTGGTGGAACTCGTGTGGCCTTTATCTTTATTTCTGGTCTTGATCTG
GTTAAGGAATGCCAACCCACTCTACAGCCATCATGAATGCCATTTCCCCAACAAGGCGATGCCCT
CAGCAGGAATGCTGCCGTGGCTCCAGGGGATCTTCTGCAATGTGAACAATCCCTGTTTTCAAAGC
CCCACCCCAGGAGAATCTCCTGGAATTGTGTCAAACTATAACAACTCCATCTTGGCAAGGGTATA
TCGAGATTTTCAAGAACTCCTCATGAATGCACCAGAGAGCCAGCACCTTGGCCGTATTTGGACAG
AGCTACACATCTTGTCCCAATTCATGGACACCCTCCGGACTCACCCGGAGAGAATTGCAGGAAGA
GGAATACGAATAAGGGATATCTTGAAAGATGAAGAAACACTGACACTATTTCTCATTAAAAACATC
GGCCTGTCTGACTCAGTGGTCTACCTTCTGATCAACTCTCAAGTCCGTCCAGAGCAGTTCGCTCA
TGGAGTCCCGGACCTGGCGCTGAAGGACATCGCCTGCAGCGAGGCCCTCCTGGAGCGCTTCAT
CATCTTCAGCCAGAGACGCGGGGCAAAGACGGTGCGCTATGCCCTGTGCTCCCTCTCCCAGGG
CACCCTACAGTGGATAGAAGACACTCTGTATGCCAACGTGGACTTCTTCAAGCTCTTCCGTGTGC
TTCCCACACTCCTAGACAGCCGTTCTCAAGGTATCAATCTGAGATCTTGGGGAGGAATATTATCT
GATATGTCACCAAGAATTCAAGAGTTTATCCATCGGCCGAGTATGCAGGACTTGCTGTGGGTGAC
CAGGCCCCTCATGCAGAATGGTGGTCCAGAGACCTTTACAAAGCTGATGGGCATCCTGTCTGAC
CTCCTGTGTGGCTACCCCGAGGGAGGTGGCTCTCGGGTGCTCTCCTTCAACTGGTATGAAGACA
ATAACTATAAGGCCTTTCTGGGGATTGACTCCACAAGGAAGGATCCTATCTATTCTTATGACAGAA
GAACAACATCCTTTTGTAATGCATTGATCCAGAGCCTGGAGTCAAATCCTTTAACCAAAATCGCTT
GGAGGGCGGCAAAGCCTTTGCTGATGGAAAAATCCTGTACACTCCTGATTCACCTGCAGCACG
AAGGATACTGAAGAATGCCAACTCAACTTTTGAAGAACTGGAACACGTTAGGAAGTTGGTCAAAG
CCTGGGAAGAAGTAGGGCCCCAGATCTGGTACTTCTTTGACAACAGCACACAGATGAACATGATC
AGAGATACCCTGGGGAACCCAACAGTAAAAGACTTTTTGAATAGGCAGCTTGGTGAAGAAGGTAT
TACTGCTGAAGCCATCCTAAACTTCCTCTACAAGGGCCCTCGGGAAAGCCAGGCTGACGACATG
GCCAACTTCGACTGGAGGGACATATTTAACATCACTGATCGCACCCTCCGCCTGGTCAATCAATA
CCTGGAGTGCTTGGTCCTGGATAAGTTTGAAAGCTACAATGATGAAACTCAGCTCACCCAACGTG
CCCTCTCTCTACTGGAGGAAAACATGTTCTGGGCCGGAGTGGTATTCCCTGACATGTATCCCTGG
ACCAGCTCTCTACCACCCCACGTGAAGTATAAGATCCGAATGGACATAGACGTGGTGGAGAAAAC
CAATAAGATTAAAGACAGGTATTGGGATTCTGGTCCCAGAGCTGATCCCGTGGAAGATTTCCGGT
ACATCTGGGGCGGGTTTGCCTATCTGCAGGACATGGTTGAACAGGGGATCACAAGGAGCCAGGT
GCAGGCGGAGGCTCCAGTTGGAATCTACCTCCAGCAGATGCCCTACCCCTGCTTCGTGGACGAT
TCTTTCATGATCATCCTGAACCGCTGTTTCCCTATCTTCATGGTGCTGGCATGGATCTACTCTGTC
TCCATGACTGTGAAGAGCATCGTCTTGGAGAAGGAGTTGCGACTGAAGGAGACCTTGAAAAATCA
GGGTGTCTCCAATGCAGTGATTTGGTGTACCTGGTTCCTGGACAGCTTCTCCATCATGTCGATGA
GCATCTTCCTCCTGACGATATTCATCATGCATGGAAGAATCCTACATTACAGCGACCCATTCATCC
TCTTCCTGTTCTTGTTGGCTTTCTCCACTGCCACCATCATGCTGTGCTTTCTGCTCAGCACCTTCT
TCTCCAAGGCCAGTCTGGCAGCAGCCTGTAGTGGTGTCATCTATTTCACCCTCTACCTGCCACAC
ATCCTGTGCTTCGCCTGGCAGGACCGCATGACCGCTGAGCTGAAGAAGGCTGTGAGCTTACTGT
CTCCGGTGGCATTTGGATTTGGCACTGAGTACCTGGTTCGCTTTGAAGAGCAAGGCCTGGGGCT
GCAGTGGAGCAACATCGGGAACAGTCCCACGGAAGGGGACGAATTCAGCTTCCTGCTGTCCATG
CAGATGATGCTCCTTGATGCTGCTGTCTATGGCTTACTCGCTTGGTACCTTGATCAGGTGTTTCCA
GGAGACTATGGAACCCCACTTCCTTGGTACTTTCTTCTACAAGAGTCGTATTGGCTTGGCGGTGA
AGGGTGTTCAACCAGAGAAGAAAGAGCCCTGGAAAAGACCGAGCCCCTAACAGAGGAAACGGA
GGATCCAGAGCACCCAGAAGGAATACACGACTCCTTCTTTGAACGTGAGCATCCAGGGTGGGTT
CCTGGGGTATGCGTGAAGAATCTGGTAAAGATTTTTGAGCCCTGTGGCCGGCCAGCTGTGGACC
GTCTGAACATCACCTTCTACGAGAACCAGATCACCGCATTCCTGGGCCACAATGGAGCTGGGAA
AACCACCACCTTGTCCATCCTGACGGGTCTGTTGCCACCAACCTCTGGGACTGTGCTCGTTGGG

GGAAGGGACATTGAAACCAGCCTGGATGCAGTCCGGCAGAGCCTTGGCATGTGTCCACAGCACA
ACATCCTGTTCCACCACCTCACGGTGGCTGAGCACATGCTGTTCTATGCCCAGCTGAAAGGAAAG
TCCCAGGAGGAGGCCCAGCTGGAGATGGAAGCCATGTTGGAGGACACAGGCCTCCACCACAAG
CGGAATGAAGAGGCTCAGGACCTATCAGGTGGCATGCAGAGAAAGCTGTCGGTTGCCATTGCCT
TTGTGGGAGATGCCAAGGTGGTGATTCTGGACGAACCCACCTCTGGGGTGGACCCTTACTCGAG
ACGCTCAATCTGGGATCTGCTCCTGAAGTATCGCTCAGGTAAGTGCACTGAGCAGAAGGGAAGA
AGCTCCGGGGGCTCTTTGTAGGGTCCTAGGAGTGCATCAAGGCGATCACATCAGTGAAAAAAAG
CCAGACAGGCGGTTAAACCAACGCAGATTAAACAGCAGGATGCAAAAATTCGCAGGTGGTCAGA
TGCCTCCTGCAGGGGGCGGCCGCGCAGTGAAAAAAATGCTTTATTTGTGAAATTTGTGATGCTAT
TGCTTTATTTGTAACCATTATAAGCTGCAATAAACAAGTTGCTCGAGTTAAGGGCGAATTCCCGAT
AAGGATCTTCCTAGAGCATGGCTACGTAGATAAGTAGCATGGCGGGTTAATCATTAACTACAAGG
AACCCCTAGTGATGGAGTTGGCCACTCCCTCTCTGCGCGCTCGCTCGCTCACTGAGGCCGGGC
GACCAAAGGTCGCCCGACGCCCGGGCTTTGCCCGGGCGGCCTCAGTGAGCGAGCGAGCGCGC
AGAGAGGGAGTGGCCAATTCGGCTGCGGCGAGCGGTATCAGCTCACTCAAAGGCGGTAATACG
GTTATCCACAGAATCAGGGGATAACGCAGGAAAGAACATGTCGCGTTGCTGGCGTTTTTCCATAG
GCTCCGCCCCCCTGACGAGCATCACAAAAATCGACGCTCAAGTCAGAGGTGGCGAAACCCGACA
GGACTATAAAGATACCAGGCGTTTCCCCCTGGAAGCTCCCTCGTGCGCTCTCCTGTTCCGACCCT
GCCGCTTACCGGATACCTGTCCGCCTTTCTCCCTTCGGGAAGCGTGGCGCTTTCTCATAGCTCAC
GCTGTAGGTATCTCAGTTCGGTGTAGGTCGTTCGCTCCAAGCTGGGCTGTGTGCACGAACCCCC
CGTTCAGCCCGACCGCTGCGCCTTATCCGGTAACTATCGTCTTGAGTCCAACCCGGTAAGACAC
GACTTATCGCCACTGGCAGCAGCCACTGGTAACAGGATTAGCAGAGCGAGGTATGTAGGCGGTG
CTACAGAGTTCTTGAAGTGGTGGCCTAACTACGGCTACACTAGAAGAACAGTATTTGGTATCTGC
GCTCTGCTGAAGCCAGTTACCTTCGGAAAAAGAGTTGGTAGCTCTTGATCCGGCAAACAAACCAC
CGCTGGTAGCGGTGGTTTTTTTGTTTGCAAGCAGCAGATTACGCGCAGAAAAAAAGGATCTCAAG
AAGATCCTTTGATCTTTTCTACGGGGTCTGACGCTCAGTGGAACGAAAACTCACGTTAAGGGATT
TTGGTCATGACTGTGGAATGTGTGTCAGTTAGGCGACATAGGTGATCTATGTAGAAGCCTAGTGG
AACAGGTTAGTTTGAGTAGCTTTAGAATGTAAATTCTGGGATCATAGTGTAGTAATCTCTAATTAAC
GGTGACGGTTTGTAAGACAGGTCTTCGCAAAATCAAGCGGCAGGTGATTTCAACAGATTCTTGCT
GATGGTTTAGGCGTACAATGCCCTGAAGAATAAGTAAGAGAATAGCACTCCTCGTCGCCTAGAAT
TACCTACCGGCGTCCACCATACCTTCGATTATCGCGCCCACTCTCCCATTAGTCGGCACAGGTGG
ATGTGTTGCGATAGCCCGCTAAGATATTCTAAGGCGTAACGCAGATGAATATTCTACAGAGTTGC
CATAGGCGTTGAACGCTTCACGGACGATAGGAATGTTGCGTATAGAGCGTGAGTCATCGAAGTG
GTGTATACACTCGTACTTAACATCTAGCCCGGCTCTATCAGTACACCAGTGCCTTGAATGACATAC
TCATCATTAAACTTTCTCAACAGTCAAACGACCAAGTGCATTTCCAAGGAGTGCGAAGGAGATTCA
TTCTCTCGCCAGCACTGTAATAGGCACTAAAAGAGTGAAGATAAGCTAGAGTGCCGTGCTAAGAC
GGTGTCGGAACAAAGCGGTCTTACGGTCAGTCGTATTTCCTGTCGAGTCCCGTCCAGTTGAGCG
TATCACTCCCAGTGTACTAGCAAGCCGAGAAGGCTGTGCTTGGAGTCAATCGGATGTAGGATGG
TCTCCAGACACCGGGCCACCACTCTTCACGCCTAGAAGCATAGAACGTCGAGCAGACATCAAAG
TCTTAGTACCGGACGTGCCGTTTCACTGCGAATATTACCTGAAGCTGTACCGTTATTGCGGAGCA
AAGTGACAGTGCTGCTCTTATCATATTTGTATTGACGACAGCCGCCTTCGCGGTTTCCTCAGACT
CTAGATCGAATACAGGCTTATTGTAGGCAGAGGCACGCCCTTGTTAGTGGCTGCGGCAATATCTT
CCGATCCCCTTGTCTAACCATGAATCAATTCTCTCATTTGAAGACCCTAATATGTCATCATTAGTGT
TTCAAATGCCACCAAATACCGCCTAGAAATGTCTATGATGTGTGTCCACTAGAAGTTGATTCACAA
ACGACTGCTAGAATCGCGTGATAGGGCATCTTGAAGTTTACATTGTTGTATCGCAAGGTACTCCG
ATCTTAATGGATGCGAAGTGGTACGGATGCAATCAAGCGCGTGAGAGCGGTACATTAGAGCGTT
CACCTACGCTACGCTAACGGGCGATTCTGATAAGAATGCACATTGCGTCGATTCATAAGATGTCT
CGACCGCATGCGCAACTTGTGAAGTGTCTACTATCCCTAAGCGCATATCTCGCACAGTAACCGAA
TATGTCGGCATCTGATGTTACCGTTGAGTTAGTGTTCAGCTCACGGAACTTATTGTATGAGTAGAG
ATTTGTAAGAGCTGTTAGTTAGCTCGCTCAGCTAATAGTTGCCCACACAACGTCAAATTAGAGAAC
GGTCGTAACATTATCGGTGGTTCTCTAACTACTATCAGTACCCACGACTCGACTCTGCCGCAGCT
AGGTATCGCCTGAAAGCCAGTCAGCGTTAAGGAGTGCTCTGACCAGGACAACAGGCGTAGTGAG
AGTTACTTGTTCGTTGCTCTTCCGACTCGGACCTGAGTTCGCCAACGACCCACTTGAGGTCTGAG
CCGGTGAAGAGAAGTAAGCATCTCGTTCGCAGCTTGCCAGCACTTTCAGAACATGACCCCTATTT
GTTTATTTTTCTAAATACATTCAAATATGTATCCGCTCATGAGACAATAACCCTGATAAATGCTTCA
ATAATATTGAAAAAGGAAGAGTGGCCGCCTCGGCCTAGGCTTTTGCAAAGATCGATCAAGAGACA
GGATGAGGATCGTTTCGCATGATTGAACAAGATGGATTGCACGCAGGTTCTCCGGCCGCTTGGG
TGGAGAGGCTATTCGGCTATGACTGGGCACAACAGACAATCGGCTGCTCTGATGCCGCCGTGTT

CCGGCTGTCAGCGCAGGGGCGCCCGGTTCTTTTTGTCAAGACCGACCTGTCCGGTGCCCTGAAT
GAACTGCAAGACGAGGCAGCGCGGCTATCGTGGCTGGCCACGACGGGCGTTCCTTGCGCAGCT
GTGCTCGACGTTGTCACTGAAGCGGGAAGGGACTGGCTGCTATTGGGCGAAGTGCCGGGGCAG
GATCTCCTGTCATCTCACCTTGCTCCTGCCGAGAAAGTATCCATCATGGCTGATGCAATGCGGCG
GCTGCATACGCTTGATCCGGCTACCTGCCCATTCGACCACCAAGCGAAACATCGCATCGAGCGA
GCACGTACTCGGATGGAAGCCGGTCTTGTCGATCAGGATGATCTGGACGAAGAGCATCAGGGGC
TCGCGCCAGCCGAACTGTTCGCCAGGCTCAAGGCGAGCATGCCCGACGGCGAGGATCTCGTCG
TGACCCATGGCGATGCCTGCTTGCCGAATATCATGGTGGAAAATGGCCGCTTTTCTGGATTCATC
GACTGTGGCCGGCTGGGTGTGGCGGACCGCTATCAGGACATAGCGTTGGCTACCCGTGATATTG
CTGAAGAGCTTGGCGGCGAATGGGCTGACCGCTTCCTCGTGCTTTACGGTATCGCCGCTCCCGA
TTCGCAGCGCATCGCCTTCTATCGCCTTCTTGACGAGTTCTTCTGAGGTACCATGATGCGTGCAT
GGTAGAATGACTCTTGATAACGGACTTCGACTAGGCAATATCCCTTGTCAACTTGTCGAGGAGAA
AAGTATTGACTGAAGCGCTCCCGGCACAACGGCCAAAGAAGTCTCAGCAATGTTCTTATTTCCGA
ATGACATGCGTCTCCTTGCGGGTAAATCGCCGACCGCAAAACTTAGGAGCCAGGATACAGATAG
GTCTAACTTAGGTTAAGGGAGTAAATCCTGGGATCGTTCAGTTGTAACCATATACTTACGCTGGG
GCTTCTCCGGCGGATGTTACTGTCACCAACCACGAGATTTGAAGTAAACGCATGATTGAGCACAT
AGCCGCGCTATCCGACAATCTCCAAATTGATAACATACCGTTCCATGAAGGCCAGAATTACTTAC
CGGCCCTTTCCATGCGTGCGCCATACCGCACTCTGCGCTTATCCGTCCGAGGGGAGAGTGTGCG
ATCCTCCGTTAAGATATTCTCACGTATGACGTAGCTATGTATTGTGCAGAGGTAGCGAAGGCGTT
GAACACTTCACAGATGGTGGGGATTCGGGCAAAGGGCGTGATAACTTGGGGACTAACATAGGCG
TAAACTACGATGGCACCAACTCAATCGCAGCTCGTGCGCCCTGAATCAACGTACTCATCTCAACT
GATTCTCGGCAATCTACGGAGCGACTTGATTATCAACACCTGTCTAGCAGTTCTAATCTTCTGCCA
ACATCGTACATAGCCTCCAAGAGATTATCATACCTATCGGCACAGAAGTGACACGACGCCGAAGG
GTAGCGGACTTCTGGTCAACCACAATTCCCCAGGGGACAGGTCCTGCGGTGCGCATCACTTTGT
AAGTGCAAGCAACCCAAGTGAGCCCAGCCTGGACTGAGCTGGTTCCTGTGTCAGGTCGAGGCTG
GGGATGACAGCTCTTGTAAACATAGTGATCAAGCGTGGCGTCGAACGGTCGAGAAACTCATAGTA
CCTCGGGTAGCAACTTACTCAGGTTATTGCTTGAAGCTGTACTATTTCAGGAGCGCTGAAGGTCT
CTTCTTCTGTAGACTGAACTCGCAAGGGTCGTGAAGTCGGTTCCTTCAATGCTTAACAAGAACAA
AGGCTTACTGTGCAGACTGGAACGCCCATCTAGCGGCTCGCGTCTTGAATGCTCGGTCCCCTTT
GTCATTGCGGATACAATCCATTTCCCTCATTCACCAGCTTGCGAAGTCTACATTGAGTAGACGAAT
GCGACCTAGAAGAGGTGCGCTTCAGAACTTGTGAGGAGTGGTTGATGCTCTATACTCCATTTGGT
GTTTCGTGCATCACCGCGATAGGCTGACAAGAGGTCTTGAACATTGAATAGCAAGGCACTTCCGG
TCTCATAGAAGAGAGCACGGGATAAGGTACGCGCGTGGTACGGGAGGATCAAGGGGCTACACG
ATAGAAAGGCTTCTCCCTCACTCGCTAGGAGGCAAATGCAGAACGCTGGTTACTACTACGATACG
TGAAACTTGTCCAACGGTTGCCCAAAGTGTTAAGTGTCTATCACCCTAGTGCCGTTTCCCGGAGA
AAACGCCAGGTTGAATCCGCATTTGAAGCTACGATGGTGAAGTCTGGGTCGAGCGCGCCGCATG
TTGATTGCGTGAGTAGGCTCGACCAAGAACCGCTAGTAGCGTCGCTGTAGAAATAGTTCTCGACA
GACCGTCGAGTTTAGAAAATGGTAGCAGCATTGTTCGCATCTCAATCAAGTATGGATTACGGTGT
TTACACTGTCCTGCGGCTACCCATCGCCTGAAATCCAGCTCGTGTCAAGCCATTGCCTCTCCGGG
ACGCCGCATGAAGTAACTACATATACCTTGCACGGGTTGACTGCGGTCCGTTCAGACTCGACCAA
GGACACAATCCAGCGATCGGTGCGGGCCTCTTCGCTATTACGC

10. pGL2-RHO-BD10-SAS-3'ABCA4-SV40pA (9858 bp)

TTGGCCACTCCCTCTCTGCGCGCTCGCTCGCTCACTGAGGCCGCCCGGGCAAAGCCCGGGCGT
CGGGCGACCTTTGGTCGCCCGGCCTCAGTGAGCGAGCGAGCGCGCAGAGAGGGAGTGGCCAA
CTCCATCACTAGGGGTTCCTTGTAGTTAATGATTAACCCGCCATGCTACTTATCTACGTAGCCATG
CTCTAGGAAGATCGGAATTCGCCCTTAAGCTAGCCCTCTCCTCCCTGACCTCAGGCTTCCTCCTA
GTGTCACCTTGGCCCCTCTTAGAAGCCAATTAGGCCCTCAGTTTCTGCAGCGGGGATTAATATGA
TTATGAACACCCCCAATCTCCCAGATGCTGATTCAGCCAGGAGCTTAGGAGGGGGAGGTCACTTT
ATAAGGGTCTGGGGGGGTCAGAACCCAGAGTCATCGGTACCACCGGTCATCTGACCACCTGCGA
ATTTTTGCATCCTGCTGTTTAATCTGCGTTGGTTTAACCGCCTGTCTGGCTTTTTTTCACTGATGTG
ATCGCCTTGATGCACTTCTAGAGGATCCCAACGAGTCTTTTGTCATCTACAGGTAGAACCATCATC
ATGTCCACTCACCACATGGACGAGGCCGACCTCCTTGGGGACCGCATTGCCATCATTGCCCAGG
GAAGGCTCTACTGCTCAGGCACCCCACTCTTCCTGAAGAACTGCTTTGGCACAGGCTTGTACTTA
ACCTTGGTGCGCAAGATGAAAAACATCCAGAGCCAAAGGAAAGGCAGTGAGGGGACCTGCAGCT
GCTCGTCTAAGGGTTTCTCCACCACGTGTCCAGCCCACGTCGATGACCTAACTCCAGAACAAGTC
CTGGATGGGGATGTAAATGAGCTGATGGATGTAGTTCTCCACCATGTTCCAGAGGCAAAGCTGGT

GGAGTGCATTGGTCAAGAACTTATCTTCCTTCTTCCAAATAAGAACTTCAAGCACAGAGCATATGC
CAGCCTTTTCAGAGAGCTGGAGGAGACGCTGGCTGACCTTGGTCTCAGCAGTTTTGGAATTTCTG
ACACTCCCCTGGAAGAGATTTTTCTGAAGGTCACGGAGGATTCTGATTCAGGACCTCTGTTTGCG
GGTGGCGCTCAGCAGAAAAGAGAAAACGTCAACCCCCGACACCCCTGCTTGGGTCCCAGAGAG
AAGGCTGGACAGACACCCCAGGACTCCAATGTCTGCTCCCCAGGGGCGCCGGCTGCTCACCCA
GAGGGCCAGCCTCCCCCAGAGCCAGAGTGCCCAGGCCCGCAGCTCAACACGGGGACACAGCT
GGTCCTCCAGCATGTGCAGGCGCTGCTGGTCAAGAGATTCCAACACACCATCCGCAGCCACAAG
GACTTCCTGGCGCAGATCGTGCTCCCGGCTACCTTTGTGTTTTTGGCTCTGATGCTTTCTATTGTT
ATCCCTCCTTTTGGCGAATACCCCGCTTTGACCCTTCACCCCTGGATATATGGGCAGCAGTACAC
CTTCTTCAGCATGGATGAACCAGGCAGTGAGCAGTTCACGGTACTTGCAGACGTCCTCCTGAATA
AGCCAGGCTTTGGCAACCGCTGCCTGAAGGAAGGGTGGCTTCCGGAGTACCCCTGTGGCAACT
CAACACCCTGGAAGACTCCTTCTGTGTCCCCAAACATCACCCAGCTGTTCCAGAAGCAGAAATGG
ACACAGGTCAACCCTTCACCATCCTGCAGGTGCAGCACCAGGGAGAAGCTCACCATGCTGCCAG
AGTGCCCCGAGGGTGCCGGGGGCCTCCCGCCCCCCCAGAGAACACAGCGCAGCACGGAAATTC
TACAAGACCTGACGGACAGGAACATCTCCGACTTCTTGGTAAAAACGTATCCTGCTCTTATAAGAA
GCAGCTTAAAGAGCAAATTCTGGGTCAATGAACAGAGGTATGGAGGAATTTCCATTGGAGGAAAG
CTCCCAGTCGTCCCCATCACGGGGGAAGCACTTGTTGGGTTTTTAAGCGACCTTGGCCGGATCA
TGAATGTGAGCGGGGGCCCTATCACTAGAGAGGCCTCTAAAGAAATACCTGATTTCCTTAAACAT
CTAGAAACTGAAGACAACATTAAGGTGTGGTTTAATAACAAAGGCTGGCATGCCCTGGTCAGCTT
TCTCAATGTGGCCCACAACGCCATCTTACGGGCCAGCCTGCCTAAGGACAGGAGCCCCGAGGA
GTATGGAATCACCGTCATTAGCCAACCCCTGAACCTGACCAAGGAGCAGCTCTCAGAGATTACAG
TGCTGACCACTTCAGTGGATGCTGTGGTTGCCATCTGCGTGATTTTCTCCATGTCCTTCGTCCCA
GCCAGCTTTGTCCTTTATTTGATCCAGGAGCGGGTGAACAAATCCAAGCACCTCCAGTTTATCAG
TGGAGTGAGCCCCACCACCTACTGGGTGACCAACTTCCTCTGGGACATCATGAATTATTCCGTGA
GTGCTGGGCTGGTGGTGGGCATCTTCATCGGGTTTCAGAAGAAAGCCTACACTTCTCCAGAAAA
CCTTCCTGCCCTTGTGGCACTGCTCCTGCTGTATGGATGGGCGGTCATTCCCATGATGTACCCAG
CATCCTTCCTGTTTGATGTCCCCAGCACAGCCTATGTGGCTTTATCTTGTGCTAATCTGTTCATCG
GCATCAACAGCAGTGCTATTACCTTCATCTTGGAATTATTTGAGAATAACCGGACGCTGCTCAGGT
TCAACGCCGTGCTGAGGAAGCTGCTCATTGTCTTCCCCCACTTCTGCCTGGGCCGGGGCCTCAT
TGACCTTGCACTGAGCCAGGCTGTGACAGATGTCTATGCCCGGTTTGGTGAGGAGCACTCTGCA
AATCCGTTCCACTGGGACCTGATTGGGAAGAACCTGTTTGCCATGGTGGTGGAAGGGGTGGTGT
ACTTCCTCCTGACCCTGCTGGTCCAGCGCCACTTCTTCCTCTCCCAATGGATTGCCGAGCCCACT
AAGGAGCCCATTGTTGATGAAGATGATGATGTGGCTGAAGAAAGACAAAGAATTATTACTGGTGG
AAATAAAACTGACATCTTAAGGCTACATGAACTAACCAAGATTTATCCAGGCACCTCCAGCCCAGC
AGTGGACAGGCTGTGTGTCGGAGTTCGCCCTGGAGAGTGCTTTGGCCTCCTGGGAGTGAATGGT
GCCGGCAAAACAACCACATTCAAGATGCTCACTGGGGACACCACAGTGACCTCAGGGGATGCCA
CCGTAGCAGGCAAGAGTATTTTAACCAATATTTCTGAAGTCCATCAAAATATGGGCTACTGTCCTC
AGTTTGATGCAATTGATGAGCTGCTCACAGGACGAGAACATCTTTACCTTTATGCCCGGCTTCGA
GGTGTACCAGCAGAAGAAATCGAAAAGGTTGCAAACTGGAGTATTAAGAGCCTGGGCCTGACTG
TCTACGCCGACTGCCTGGCTGGCACGTACAGTGGGGGCAACAAGCGGAAACTCTCCACAGCCAT
CGCACTCATTGGCTGCCCACCGCTGGTGCTGCTGGATGAGCCCACCACAGGGATGGACCCCCA
GGCACGCCGCATGCTGTGGAACGTCATCGTGAGCATCATCAGAGAAGGGAGGGCTGTGGTCCT
CACATCCCACAGCATGGAAGAATGTGAGGCACTGTGTACCCGGCTGGCCATCATGGTAAAGGGC
GCCTTTCGATGTATGGGCACCATTCAGCATCTCAAGTCCAAATTTGGAGATGGCTATATCGTCAC
AATGAAGATCAAATCCCCGAAGGACGACCTGCTTCCTGACCTGAACCCTGTGGAGCAGTTCTTCC
AGGGGAACTTCCCAGGCAGTGTGCAGAGGGAGAGGCACTACAACATGCTCCAGTTCCAGGTCTC
CTCCTCCTCCCTGGCGAGGATCTTCCAGCTCCTCCTCTCCCACAAGGACAGCCTGCTCATCGAG
GAGTACTCAGTCACACAGACCACACTGGACCAGGTGTTTGTAAATTTTGCTAAACAGCAGACTGA
AAGTCATGACCTCCCTCTGCACCCTCGAGCTGCTGGAGCCAGTCGACAAGCCCAGGACTAACCT
GCAGGGCAGTGAAAAAAATGCTTTATTTGTGAAATTTGTGATGCTATTGCTTTATTTGTAACCATTA
TAAGCTGCAATAAACAAGTTGCGGCCGCGTCGAGTTAAGGGCGAATTCCCGATAAGGATCTTCCT
AGAGCATGGCTACGTAGATAAGTAGCATGGCGGGTTAATCATTAACTACAAGGAACCCCTAGTGA
TGGAGTTGGCCACTCCCTCTCTGCGCGCTCGCTCGCTCACTGAGGCCGGGCGACCAAAGGTCG
CCCGACGCCCGGGCTTTGCCCGGGCGGCCTCAGTGAGCGAGCGAGCGCGCAGAGAGGGAGTG
GCCAATTCGGCTGCGGCGAGCGGTATCAGCTCACTCAAAGGCGGTAATACGGTTATCCACAGAA
TCAGGGGATAACGCAGGAAAGAACATGTCGCGTTGCTGGCGTTTTTCCATAGGCTCCGCCCCCC
TGACGAGCATCACAAAAATCGACGCTCAAGTCAGAGGTGGCGAAACCCGACAGGACTATAAAGA

TACCAGGCGTTTCCCCCTGGAAGCTCCCTCGTGCGCTCTCCTGTTCCGACCCTGCCGCTTACCG
GATACCTGTCCGCCTTTCTCCCTTCGGGAAGCGTGGCGCTTTCTCATAGCTCACGCTGTAGGTAT
CTCAGTTCGGTGTAGGTCGTTCGCTCCAAGCTGGGCTGTGTGCACGAACCCCCCGTTCAGCCCG
ACCGCTGCGCCTTATCCGGTAACTATCGTCTTGAGTCCAACCCGGTAAGACACGACTTATCGCCA
CTGGCAGCAGCCACTGGTAACAGGATTAGCAGAGCGAGGTATGTAGGCGGTGCTACAGAGTTCT
TGAAGTGGTGGCCTAACTACGGCTACACTAGAAGAACAGTATTTGGTATCTGCGCTCTGCTGAAG
CCAGTTACCTTCGGAAAAAGAGTTGGTAGCTCTTGATCCGGCAAACAAACCACCGCTGGTAGCG
GTGGTTTTTTTGTTTGCAAGCAGCAGATTACGCGCAGAAAAAAAGGATCTCAAGAAGATCCTTTGA
TCTTTTCTACGGGGTCTGACGCTCAGTGGAACGAAAACTCACGTTAAGGGATTTTGGTCATGACT
GTGGAATGTGTGTCAGTTAGGCGACATAGGTGATCTATGTAGAAGCCTAGTGGAACAGGTTAGTT
TGAGTAGCTTTAGAATGTAAATTCTGGGATCATAGTGTAGTAATCTCTAATTAACGGTGACGGTTT
GTAAGACAGGTCTTCGCAAAATCAAGCGGCAGGTGATTTCAACAGATTCTTGCTGATGGTTTAGG
CGTACAATGCCCTGAAGAATAAGTAAGAGAATAGCACTCCTCGTCGCCTAGAATTACCTACCGGC
GTCCACCATACCTTCGATTATCGCGCCCACTCTCCCATTAGTCGGCACAGGTGGATGTGTTGCGA
TAGCCCGCTAAGATATTCTAAGGCGTAACGCAGATGAATATTCTACAGAGTTGCCATAGGCGTTG
AACGCTTCACGGACGATAGGAATGTTGCGTATAGAGCGTGAGTCATCGAAGTGGTGTATACACTC
GTACTTAACATCTAGCCCGGCTCTATCAGTACACCAGTGCCTTGAATGACATACTCATCATTAAAC
TTTCTCAACAGTCAAACGACCAAGTGCATTTCCAAGGAGTGCGAAGGAGATTCATTCTCTCGCCA
GCACTGTAATAGGCACTAAAAGAGTGAAGATAAGCTAGAGTGCCGTGCTAAGACGGTGTCGGAA
CAAAGCGGTCTTACGGTCAGTCGTATTTCCTGTCGAGTCCCGTCCAGTTGAGCGTATCACTCCCA
GTGTACTAGCAAGCCGAGAAGGCTGTGCTTGGAGTCAATCGGATGTAGGATGGTCTCCAGACAC
CGGGCCACCACTCTTCACGCCTAGAAGCATAGAACGTCGAGCAGACATCAAAGTCTTAGTACCG
GACGTGCCGTTTCACTGCGAATATTACCTGAAGCTGTACCGTTATTGCGGAGCAAAGTGACAGTG
CTGCTCTTATCATATTTGTATTGACGACAGCCGCCTTCGCGGTTTCCTCAGACTCTAGATCGAATA
CAGGCTTATTGTAGGCAGAGGCACGCCCTTGTTAGTGGCTGCGGCAATATCTTCCGATCCCCTTG
TCTAACCATGAATCAATTCTCTCATTTGAAGACCCTAATATGTCATCATTAGTGTTTCAAATGCCAC
CAAATACCGCCTAGAAATGTCTATGATGTGTGTCCACTAGAAGTTGATTCACAAACGACTGCTAGA
ATCGCGTGATAGGGCATCTTGAAGTTTACATTGTTGTATCGCAAGGTACTCCGATCTTAATGGATG
CGAAGTGGTACGGATGCAATCAAGCGCGTGAGAGCGGTACATTAGAGCGTTCACCTACGCTACG
CTAACGGGCGATTCTGATAAGAATGCACATTGCGTCGATTCATAAGATGTCTCGACCGCATGCGC
AACTTGTGAAGTGTCTACTATCCCTAAGCGCATATCTCGCACAGTAACCGAATATGTCGGCATCT
GATGTTACCGTTGAGTTAGTGTTCAGCTCACGGAACTTATTGTATGAGTAGAGATTTGTAAGAGCT
GTTAGTTAGCTCGCTCAGCTAATAGTTGCCCACACAACGTCAAATTAGAGAACGGTCGTAACATTA
TCGGTGGTTCTCTAACTACTATCAGTACCCACGACTCGACTCTGCCGCAGCTAGGTATCGCCTGA
AAGCCAGTCAGCGTTAAGGAGTGCTCTGACCAGGACAACAGGCGTAGTGAGAGTTACTTGTTCG
TTGCTCTTCCGACTCGGACCTGAGTTCGCCAACGACCCACTTGAGGTCTGAGCCGGTGAAGAGA
AGTAAGCATCTCGTTCGCAGCTTGCCAGCACTTTCAGAACATGACCCCTATTTGTTTATTTTTCTA
AATACATTCAAATATGTATCCGCTCATGAGACAATAACCCTGATAAATGCTTCAATAATATTGAAAA
AGGAAGAGTGGCCGCCTCGGCCTAGGCTTTTGCAAAGATCGATCAAGAGACAGGATGAGGATCG
TTTCGCATGATTGAACAAGATGGATTGCACGCAGGTTCTCCGGCCGCTTGGGTGGAGAGGCTAT
TCGGCTATGACTGGGCACAACAGACAATCGGCTGCTCTGATGCCGCCGTGTTCCGGCTGTCAGC
GCAGGGGCGCCCGGTTCTTTTTGTCAAGACCGACCTGTCCGGTGCCCTGAATGAACTGCAAGAC
GAGGCAGCGCGGCTATCGTGGCTGGCCACGACGGGCGTTCCTTGCGCAGCTGTGCTCGACGTT
GTCACTGAAGCGGGAAGGGACTGGCTGCTATTGGGCGAAGTGCCGGGGCAGGATCTCCTGTCA
TCTCACCTTGCTCCTGCCGAGAAAGTATCCATCATGGCTGATGCAATGCGGCGGCTGCATACGCT
TGATCCGGCTACCTGCCCATTCGACCACCAAGCGAAACATCGCATCGAGCGAGCACGTACTCGG
ATGGAAGCCGGTCTTGTCGATCAGGATGATCTGGACGAAGAGCATCAGGGGCTCGCGCCAGCC
GAACTGTTCGCCAGGCTCAAGGCGAGCATGCCCGACGGCGAGGATCTCGTCGTGACCCATGGC
GATGCCTGCTTGCCGAATATCATGGTGGAAAATGGCCGCTTTTCTGGATTCATCGACTGTGGCCG
GCTGGGTGTGGCGGACCGCTATCAGGACATAGCGTTGGCTACCCGTGATATTGCTGAAGAGCTT
GGCGGCGAATGGGCTGACCGCTTCCTCGTGCTTTACGGTATCGCCGCTCCCGATTCGCAGCGCA
TCGCCTTCTATCGCCTTCTTGACGAGTTCTTCTGAGGTACCATGATGCGTGCATGGTAGAATGAC
TCTTGATAACGGACTTCGACTAGGCAATATCCCTTGTCAACTTGTCGAGGAGAAAAGTATTGACTG
AAGCGCTCCCGGCACAACGGCCAAAGAAGTCTCAGCAATGTTCTTATTTCCGAATGACATGCGTC
TCCTTGCGGGTAAATCGCCGACCGCAAAACTTAGGAGCCAGGATACAGATAGGTCTAACTTAGGT
TAAGGGAGTAAATCCTGGGATCGTTCAGTTGTAACCATATACTTACGCTGGGGCTTCTCCGGCGG
ATGTTACTGTCACCAACCACGAGATTTGAAGTAAACGCATGATTGAGCACATAGCCGCGCTATCC

GACAATCTCCAAATTGATAACATACCGTTCCATGAAGGCCAGAATTACTTACCGGCCCTTTCCATG
CGTGCGCCATACCGCACTCTGCGCTTATCCGTCCGAGGGGAGAGTGTGCGATCCTCCGTTAAGA
TATTCTCACGTATGACGTAGCTATGTATTGTGCAGAGGTAGCGAAGGCGTTGAACACTTCACAGA
TGGTGGGGATTCGGGCAAAGGGCGTGATAACTTGGGGACTAACATAGGCGTAAACTACGATGGC
ACCAACTCAATCGCAGCTCGTGCGCCCTGAATCAACGTACTCATCTCAACTGATTCTCGGCAATC
TACGGAGCGACTTGATTATCAACACCTGTCTAGCAGTTCTAATCTTCTGCCAACATCGTACATAGC
CTCCAAGAGATTATCATACCTATCGGCACAGAAGTGACACGACGCCGAAGGGTAGCGGACTTCT
GGTCAACCACAATTCCCCAGGGGACAGGTCCTGCGGTGCGCATCACTTTGTAAGTGCAAGCAAC
CCAAGTGAGCCCAGCCTGGACTGAGCTGGTTCCTGTGTCAGGTCGAGGCTGGGGATGACAGCT
CTTGTAAACATAGTGATCAAGCGTGGCGTCGAACGGTCGAGAAACTCATAGTACCTCGGGTAGCA
ACTTACTCAGGTTATTGCTTGAAGCTGTACTATTTCAGGAGCGCTGAAGGTCTCTTCTTCTGTAGA
CTGAACTCGCAAGGGTCGTGAAGTCGGTTCCTTCAATGCTTAACAAGAACAAAGGCTTACTGTGC
AGACTGGAACGCCCATCTAGCGGCTCGCGTCTTGAATGCTCGGTCCCCTTTGTCATTGCGGATA
CAATCCATTTCCCTCATTCACCAGCTTGCGAAGTCTACATTGAGTAGACGAATGCGACCTAGAAG
AGGTGCGCTTCAGAACTTGTGAGGAGTGGTTGATGCTCTATACTCCATTTGGTGTTTCGTGCATC
ACCGCGATAGGCTGACAAGAGGTCTTGAACATTGAATAGCAAGGCACTTCCGGTCTCATAGAAGA
GAGCACGGGATAAGGTACGCGCGTGGTACGGGAGGATCAAGGGGCTACACGATAGAAAGGCTT
CTCCCTCACTCGCTAGGAGGCAAATGCAGAACGCTGGTTACTACTACGATACGTGAAACTTGTCC
AACGGTTGCCCAAAGTGTTAAGTGTCTATCACCCTAGTGCCGTTTCCCGGAGAAAACGCCAGGTT
GAATCCGCATTTGAAGCTACGATGGTGAAGTCTGGGTCGAGCGCGCCGCATGTTGATTGCGTGA
GTAGGCTCGACCAAGAACCGCTAGTAGCGTCGCTGTAGAAATAGTTCTCGACAGACCGTCGAGT
TTAGAAAATGGTAGCAGCATTGTTCGCATCTCAATCAAGTATGGATTACGGTGTTTACACTGTCCT
GCGGCTACCCATCGCCTGAAATCCAGCTCGTGTCAAGCCATTGCCTCTCCGGGACGCCGCATGA
AGTAACTACATATACCTTGCACGGGTTGACTGCGGTCCGTTCAGACTCGACCAAGGACACAATCC
AGCGATCGGTGCGGGCCTCTTCGCTATTACGC

11. **pGL2-GRK1-5’ABCA4-BD10-SDS-SV40pA (9929 bp)**

CAGCTGCGCGCTCGCTCGCTCACTGAGGCCGCCCGGGCAAAGCCCGGGCGTCGGGCGACCTTT
GGTCGCCCGGCCTCAGTGAGCGAGCGAGCGCGCAGAGAGGGAGTGGCCAACTCCATCACTAGG
GGTTCCTTGTAGTTAATGATTAACCCGCCATGCTACTTATCTACGTAGCCATGCTCTAGGAAGATC
GGAATTCGCCCTTAAGGGCGCGCCGTTTAAATAGCTAGCGGGCCCCAGAAGCCTGGTGGTTGTT
TGTCCTTCTCAGGGGAAAAGTGAGGCGGCCCCTTGGAGGAAGGGGCCGGGCAGAATGATCTAA
TCGGATTCCAAGCAGCTCAGGGGATTGTCTTTTCTAGCACCTTCTTGCCACTCCTAAGCGTCCT
CCGTGACCCCGGCTGGGATTTAGCCTGGTGCTGTGTCAGCCCCGGTCTCCCAGGGGCTTCCCA
GTGGTCCCCAGGAACCCTCGACAGGGCCCGGTCTCTCTCGTCCAGCAAGGGCAGGGACGGGCC
ACAGGCCAAGGGCACCGGTGCCACCATGGGCTTCGTGAGACAGATACAGCTTTTGCTCTGGAAG
AACTGGACCCTGCGGAAAAGGCAAAAGATTCGCTTTGTGGTGGAACTCGTGTGGCCTTTATCTTT
ATTTCTGGTCTTGATCTGGTTAAGGAATGCCAACCCACTCTACAGCCATCATGAATGCCATTTCCC
CAACAAGGCGATGCCCTCAGCAGGAATGCTGCCGTGGCTCCAGGGGATCTTCTGCAATGTGAAC
AATCCCTGTTTTCAAAGCCCCACCCCAGGAGAATCTCCTGGAATTGTGTCAAACTATAACAACTCC
ATCTTGGCAAGGGTATATCGAGATTTTCAAGAACTCCTCATGAATGCACCAGAGAGCCAGCACCT
TGGCCGTATTTGGACAGAGCTACACATCTTGTCCCAATTCATGGACACCCTCCGGACTCACCCGG
AGAGAATTGCAGGAAGAGGAATACGAATAAGGGATATCTTGAAAGATGAAGAAACACTGACACTA
TTTCTCATTAAAAACATCGGCCTGTCTGACTCAGTGGTCTACCTTCTGATCAACTCTCAAGTCCGT
CCAGAGCAGTTCGCTCATGGAGTCCCGGACCTGGCGCTGAAGGACATCGCCTGCAGCGAGGCC
CTCCTGGAGCGCTTCATCATCTTCAGCCAGAGACGCGGGGCAAAGACGGTGCGCTATGCCCTGT
GCTCCCTCTCCCAGGGCACCCTACAGTGGATAGAAGACACTCTGTATGCCAACGTGGACTTCTTC
AAGCTCTTCCGTGTGCTTCCCACACTCCTAGACAGCCGTTCTCAAGGTATCAATCTGAGATCTTG
GGGAGGAATATTATCTGATATGTCACCAAGAATTCAAGAGTTTATCCATCGGCCGAGTATGCAGG
ACTTGCTGTGGGTGACCAGGCCCCTCATGCAGAATGGTGGTCCAGAGACCTTTACAAAGCTGAT
GGGCATCCTGTCTGACCTCCTGTGTGGCTACCCCGAGGGAGGTGGCTCTCGGGTGCTCTCCTTC
AACTGGTATGAAGACAATAACTATAAGGCCTTTCTGGGGATTGACTCCACAAGGAAGGATCCTAT
CTATTCTTATGACAGAAGAACAACATCCTTTTGTAATGCATTGATCCAGAGCCTGGAGTCAAATCC
TTTAACCAAAATCGCTTGGAGGGCGGCAAAGCCTTTGCTGATGGAAAAATCCTGTACACTCCTG
ATTCACCTGCAGCACGAAGGATACTGAAGAATGCCAACTCAACTTTTGAAGAACTGGAACACGTT
AGGAAGTTGGTCAAAGCCTGGGAAGAAGTAGGGCCCCAGATCTGGTACTTCTTTGACAACAGCA
CACAGATGAACATGATCAGAGATACCCTGGGGAACCCAACAGTAAAAGACTTTTTGAATAGGCAG

CTTGGTGAAGAAGGTATTACTGCTGAAGCCATCCTAAACTTCCTCTACAAGGGCCCTCGGGAAAG
CCAGGCTGACGACATGGCCAACTTCGACTGGAGGGACATATTTAACATCACTGATCGCACCCTCC
GCCTGGTCAATCAATACCTGGAGTGCTTGGTCCTGGATAAGTTTGAAAGCTACAATGATGAAACT
CAGCTCACCCAACGTGCCCTCTCTCTACTGGAGGAAAACATGTTCTGGGCCGGAGTGGTATTCC
CTGACATGTATCCCTGGACCAGCTCTCTACCACCCCACGTGAAGTATAAGATCCGAATGGACATA
GACGTGGTGGAGAAAACCAATAAGATTAAAGACAGGTATTGGGATTCTGGTCCCAGAGCTGATCC
CGTGGAAGATTTCCGGTACATCTGGGGCGGGTTTGCCTATCTGCAGGACATGGTTGAACAGGGG
ATCACAAGGAGCCAGGTGCAGGCGGAGGCTCCAGTTGGAATCTACCTCCAGCAGATGCCCTACC
CCTGCTTCGTGGACGATTCTTTCATGATCATCCTGAACCGCTGTTTCCCTATCTTCATGGTGCTGG
CATGGATCTACTCTGTCTCCATGACTGTGAAGAGCATCGTCTTGGAGAAGGAGTTGCGACTGAAG
GAGACCTTGAAAAATCAGGGTGTCTCCAATGCAGTGATTTGGTGTACCTGGTTCCTGGACAGCTT
CTCCATCATGTCGATGAGCATCTTCCTCCTGACGATATTCATCATGCATGGAAGAATCCTACATTA
CAGCGACCCATTCATCCTCTTCCTGTTCTTGTTGGCTTTCTCCACTGCCACCATCATGCTGTGCTT
TCTGCTCAGCACCTTCTTCTCCAAGGCCAGTCTGGCAGCAGCCTGTAGTGGTGTCATCTATTTCA
CCCTCTACCTGCCACACATCCTGTGCTTCGCCTGGCAGGACCGCATGACCGCTGAGCTGAAGAA
GGCTGTGAGCTTACTGTCTCCGGTGGCATTTGGATTTGGCACTGAGTACCTGGTTCGCTTTGAAG
AGCAAGGCCTGGGGCTGCAGTGGAGCAACATCGGGAACAGTCCCACGGAAGGGGACGAATTCA
GCTTCCTGCTGTCCATGCAGATGATGCTCCTTGATGCTGCTGTCTATGGCTTACTCGCTTGGTAC
CTTGATCAGGTGTTTCCAGGAGACTATGGAACCCCACTTCCTTGGTACTTTCTTCTACAAGAGTCG
TATTGGCTTGGCGGTGAAGGGTGTTCAACCAGAGAAGAAAGAGCCCTGGAAAAGACCGAGCCCC
TAACAGAGGAAACGGAGGATCCAGAGCACCCAGAAGGAATACACGACTCCTTCTTTGAACGTGA
GCATCCAGGGTGGGTTCCTGGGGTATGCGTGAAGAATCTGGTAAAGATTTTTGAGCCCTGTGGC
CGGCCAGCTGTGGACCGTCTGAACATCACCTTCTACGAGAACCAGATCACCGCATTCCTGGGCC
ACAATGGAGCTGGGAAAACCACCACCTTGTCCATCCTGACGGGTCTGTTGCCACCAACCTCTGG
GACTGTGCTCGTTGGGGGAAGGGACATTGAAACCAGCCTGGATGCAGTCCGGCAGAGCCTTGG
CATGTGTCCACAGCACAACATCCTGTTCCACCACCTCACGGTGGCTGAGCACATGCTGTTCTATG
CCCAGCTGAAAGGAAAGTCCCAGGAGGAGGCCCAGCTGGAGATGGAAGCCATGTTGGAGGACA
CAGGCCTCCACCACAAGCGGAATGAAGAGGCTCAGGACCTATCAGGTGGCATGCAGAGAAAGCT
GTCGGTTGCCATTGCCTTTGTGGGAGATGCCAAGGTGGTGATTCTGGACGAACCCACCTCTGGG
GTGGACCCTTACTCGAGACGCTCAATCTGGGATCTGCTCCTGAAGTATCGCTCAGGTAAGTGCAC
TGAGCAGAAGGGAAGAAGCTCCGGGGGCTCTTTGTAGGGTCCTAGGAGTGCATCAAGGCGATCA
CATCAGTGAAAAAAAGCCAGACAGGCGGTTAAACCAACGCAGATTAAACAGCAGGATGCAAAAAT
TCGCAGGTGGTCAGATGCCTCCTGCAGGGGGCGGCCGCGCAGTGAAAAAAATGCTTTATTTGTG
AAATTTGTGATGCTATTGCTTTATTTGTAACCATTATAAGCTGCAATAAACAAGTTGCTCGAGTTAA
GGGCGAATTCCCGATAAGGATCTTCCTAGAGCATGGCTACGTAGATAAGTAGCATGGCGGGTTA
ATCATTAACTACAAGGAACCCCTAGTGATGGAGTTGGCCACTCCCTCTCTGCGCGCTCGCTCGCT
CACTGAGGCCGGGCGACCAAAGGTCGCCCGACGCCCGGGCTTTGCCCGGGCGGCCTCAGTGA
GCGAGCGAGCGCGCAGCTGGGCCTCAGTGAGCGAGCGAGCGCGCAGCTGCATTAATGAATCGG
CCAACGCGCGGGGAGAGGCGGTTTGCGTATTGGGCGCTCTTCCGCTTCCTCGCTCACTGACTCG
CTGCGCTCGGTCGTTCGGCTGCGGCGAGCGGTATCAGCTCACTCAAAGGCGGTAATACGGTTAT
CCACAGAATCAGGGGATAACGCAGGAAAGAACATGTCGCGTTGCTGGCGTTTTTCCATAGGCTC
CGCCCCCCTGACGAGCATCACAAAAATCGACGCTCAAGTCAGAGGTGGCGAAACCCGACAGGAC
TATAAAGATACCAGGCGTTTCCCCCTGGAAGCTCCCTCGTGCGCTCTCCTGTTCCGACCCTGCC
GCTTACCGGATACCTGTCCGCCTTTCTCCCTTCGGGAAGCGTGGCGCTTTCTCATAGCTCACGCT
GTAGGTATCTCAGTTCGGTGTAGGTCGTTCGCTCCAAGCTGGGCTGTGTGCACGAACCCCCCGT
TCAGCCCGACCGCTGCGCCTTATCCGGTAACTATCGTCTTGAGTCCAACCCGGTAAGACACGAC
TTATCGCCACTGGCAGCAGCCACTGGTAACAGGATTAGCAGAGCGAGGTATGTAGGCGGTGCTA
CAGAGTTCTTGAAGTGGTGGCCTAACTACGGCTACACTAGAAGAACAGTATTTGGTATCTGCGCT
CTGCTGAAGCCAGTTACCTTCGGAAAAAGAGTTGGTAGCTCTTGATCCGGCAAACAAACCACCGC
TGGTAGCGGTGGTTTTTTTGTTTGCAAGCAGCAGATTACGCGCAGAAAAAAAGGATCTCAAGAAG
ATCCTTTGATCTTTTCTACGGGGTCTGACGCTCAGTGGAACGAAAACTCACGTTAAGGGATTTTG
GTCATGACTGTGGAATGTGTGTCAGTTAGGCGACATAGGTGATCTATGTAGAAGCCTAGTGGAAC
AGGTTAGTTTGAGTAGCTTTAGAATGTAAATTCTGGGATCATAGTGTAGTAATCTCTAATTAACGG
TGACGGTTTGTAAGACAGGTCTTCGCAAAATCAAGCGGCAGGTGATTTCAACAGATTCTTGCTGA
TGGTTTAGGCGTACAATGCCCTGAAGAATAAGTAAGAGAATAGCACTCCTCGTCGCCTAGAATTA
CCTACCGGCGTCCACCATACCTTCGATTATCGCGCCCACTCTCCCATTAGTCGGCACAGGTGGAT
GTGTTGCGATAGCCCGCTAAGATATTCTAAGGCGTAACGCAGATGAATATTCTACAGAGTTGCCA

TAGGCGTTGAACGCTTCACGGACGATAGGAATGTTGCGTATAGAGCGTGAGTCATCGAAGTGGT
GTATACACTCGTACTTAACATCTAGCCCGGCTCTATCAGTACACCAGTGCCTTGAATGACATACTC
ATCATTAAACTTTCTCAACAGTCAAACGACCAAGTGCATTTCCAAGGAGTGCGAAGGAGATTCATT
CTCTCGCCAGCACTGTAATAGGCACTAAAAGAGTGAAGATAAGCTAGAGTGCCGTGCTAAGACG
GTGTCGGAACAAAGCGGTCTTACGGTCAGTCGTATTTCCTGTCGAGTCCCGTCCAGTTGAGCGTA
TCACTCCCAGTGTACTAGCAAGCCGAGAAGGCTGTGCTTGGAGTCAATCGGATGTAGGATGGTC
TCCAGACACCGGGCCACCACTCTTCACGCCTAGAAGCATAGAACGTCGAGCAGACATCAAAGTC
TTAGTACCGGACGTGCCGTTTCACTGCGAATATTACCTGAAGCTGTACCGTTATTGCGGAGCAAA
GTGACAGTGCTGCTCTTATCATATTTGTATTGACGACAGCCGCCTTCGCGGTTTCCTCAGACTCTA
GATCGAATACAGGCTTATTGTAGGCAGAGGCACGCCCTTGTTAGTGGCTGCGGCAATATCTTCCG
ATCCCCTTGTCTAACCATGAATCAATTCTCTCATTTGAAGACCCTAATATGTCATCATTAGTGTTTC
AAATGCCACCAAATACCGCCTAGAAATGTCTATGATGTGTGTCCACTAGAAGTTGATTCACAAACG
ACTGCTAGAATCGCGTGATAGGGCATCTTGAAGTTTACATTGTTGTATCGCAAGGTACTCCGATCT
TAATGGATGCGAAGTGGTACGGATGCAATCAAGCGCGTGAGAGCGGTACATTAGAGCGTTCACC
TACGCTACGCTAACGGGCGATTCTGATAAGAATGCACATTGCGTCGATTCATAAGATGTCTCGAC
CGCATGCGCAACTTGTGAAGTGTCTACTATCCCTAAGCGCATATCTCGCACAGTAACCGAATATG
TCGGCATCTGATGTTACCGTTGAGTTAGTGTTCAGCTCACGGAACTTATTGTATGAGTAGAGATTT
GTAAGAGCTGTTAGTTAGCTCGCTCAGCTAATAGTTGCCCACACAACGTCAAATTAGAGAACGGT
CGTAACATTATCGGTGGTTCTCTAACTACTATCAGTACCCACGACTCGACTCTGCCGCAGCTAGG
TATCGCCTGAAAGCCAGTCAGCGTTAAGGAGTGCTCTGACCAGGACAACAGGCGTAGTGAGAGT
TACTTGTTCGTTGCTCTTCCGACTCGGACCTGAGTTCGCCAACGACCCACTTGAGGTCTGAGCCG
GTGAAGAGAAGTAAGCATCTCGTTCGCAGCTTGCCAGCACTTTCAGAACATGACCCCTATTTGTT
TATTTTTCTAAATACATTCAAATATGTATCCGCTCATGAGACAATAACCCTGATAAATGCTTCAATA
ATATTGAAAAAGGAAGAGTGGCCGCCTCGGCCTAGGCTTTTGCAAAGATCGATCAAGAGACAGG
ATGAGGATCGTTTCGCATGATTGAACAAGATGGATTGCACGCAGGTTCTCCGGCCGCTTGGGTG
GAGAGGCTATTCGGCTATGACTGGGCACAACAGACAATCGGCTGCTCTGATGCCGCCGTGTTCC
GGCTGTCAGCGCAGGGGCGCCCGGTTCTTTTTGTCAAGACCGACCTGTCCGGTGCCCTGAATGA
ACTGCAAGACGAGGCAGCGCGGCTATCGTGGCTGGCCACGACGGGCGTTCCTTGCGCAGCTGT
GCTCGACGTTGTCACTGAAGCGGGAAGGGACTGGCTGCTATTGGGCGAAGTGCCGGGGCAGGA
TCTCCTGTCATCTCACCTTGCTCCTGCCGAGAAAGTATCCATCATGGCTGATGCAATGCGGCGGC
TGCATACGCTTGATCCGGCTACCTGCCCATTCGACCACCAAGCGAAACATCGCATCGAGCGAGC
ACGTACTCGGATGGAAGCCGGTCTTGTCGATCAGGATGATCTGGACGAAGAGCATCAGGGGCTC
GCGCCAGCCGAACTGTTCGCCAGGCTCAAGGCGAGCATGCCCGACGGCGAGGATCTCGTCGTG
ACCCATGGCGATGCCTGCTTGCCGAATATCATGGTGGAAAATGGCCGCTTTTCTGGATTCATCGA
CTGTGGCCGGCTGGGTGTGGCGGACCGCTATCAGGACATAGCGTTGGCTACCCGTGATATTGCT
GAAGAGCTTGGCGGCGAATGGGCTGACCGCTTCCTCGTGCTTTACGGTATCGCCGCTCCCGATT
CGCAGCGCATCGCCTTCTATCGCCTTCTTGACGAGTTCTTCTGAGGTACCATGATGCGTGCATGG
TAGAATGACTCTTGATAACGGACTTCGACTAGGCAATATCCCTTGTCAACTTGTCGAGGAGAAAA
GTATTGACTGAAGCGCTCCCGGCACAACGGCCAAAGAAGTCTCAGCAATGTTCTTATTTCCGAAT
GACATGCGTCTCCTTGCGGGTAAATCGCCGACCGCAAAACTTAGGAGCCAGGATACAGATAGGT
CTAACTTAGGTTAAGGGAGTAAATCCTGGGATCGTTCAGTTGTAACCATATACTTACGCTGGGGC
TTCTCCGGCGGATGTTACTGTCACCAACCACGAGATTTGAAGTAAACGCATGATTGAGCACATAG
CCGCGCTATCCGACAATCTCCAAATTGATAACATACCGTTCCATGAAGGCCAGAATTACTTACCG
GCCCTTTCCATGCGTGCGCCATACCGCACTCTGCGCTTATCCGTCCGAGGGGAGAGTGTGCGAT
CCTCCGTTAAGATATTCTCACGTATGACGTAGCTATGTATTGTGCAGAGGTAGCGAAGGCGTTGA
ACACTTCACAGATGGTGGGGATTCGGGCAAAGGGCGTGATAACTTGGGGACTAACATAGGCGTA
AACTACGATGGCACCAACTCAATCGCAGCTCGTGCGCCCTGAATCAACGTACTCATCTCAACTGA
TTCTCGGCAATCTACGGAGCGACTTGATTATCAACACCTGTCTAGCAGTTCTAATCTTCTGCCAAC
ATCGTACATAGCCTCCAAGAGATTATCATACCTATCGGCACAGAAGTGACACGACGCCGAAGGGT
AGCGGACTTCTGGTCAACCACAATTCCCCAGGGGACAGGTCCTGCGGTGCGCATCACTTTGTAA
GTGCAAGCAACCCAAGTGAGCCCAGCCTGGACTGAGCTGGTTCCTGTGTCAGGTCGAGGCTGG
GGATGACAGCTCTTGTAAACATAGTGATCAAGCGTGGCGTCGAACGGTCGAGAAACTCATAGTAC
CTCGGGTAGCAACTTACTCAGGTTATTGCTTGAAGCTGTACTATTTCAGGAGCGCTGAAGGTCTC
TTCTTCTGTAGACTGAACTCGCAAGGGTCGTGAAGTCGGTTCCTTCAATGCTTAACAAGAACAAA
GGCTTACTGTGCAGACTGGAACGCCCATCTAGCGGCTCGCGTCTTGAATGCTCGGTCCCCTTTG
TCATTGCGGATACAATCCATTTCCCTCATTCACCAGCTTGCGAAGTCTACATTGAGTAGACGAATG
CGACCTAGAAGAGGTGCGCTTCAGAACTTGTGAGGAGTGGTTGATGCTCTATACTCCATTTGGTG

TTTCGTGCATCACCGCGATAGGCTGACAAGAGGTCTTGAACATTGAATAGCAAGGCACTTCCGGT
CTCATAGAAGAGAGCACGGGATAAGGTACGCGCGTGGTACGGGAGGATCAAGGGGCTACACGA
TAGAAAGGCTTCTCCCTCACTCGCTAGGAGGCAAATGCAGAACGCTGGTTACTACTACGATACGT
GAAACTTGTCCAACGGTTGCCCAAAGTGTTAAGTGTCTATCACCCTAGTGCCGTTTCCCGGAGAA
AACGCCAGGTTGAATCCGCATTTGAAGCTACGATGGTGAAGTCTGGGTCGAGCGCGCCGCATGT
TGATTGCGTGAGTAGGCTCGACCAAGAACCGCTAGTAGCGTCGCTGTAGAAATAGTTCTCGACA
GACCGTCGAGTTTAGAAAATGGTAGCAGCATTGTTCGCATCTCAATCAAGTATGGATTACGGTGT
TTACACTGTCCTGCGGCTACCCATCGCCTGAAATCCAGCTCGTGTCAAGCCATTGCCTCTCCGGG
ACGCCGCATGAAGTAACTACATATACCTTGCACGGGTTGACTGCGGTCCGTTCAGACTCGACCAA
GGACACAATCCAGCGATCGGTGCGGGCCTCTTCGCTATTACGC

12. pGL2-GRK1-BD10-SAS-3’ABCA4-SV40pA (10045 bp)

CAGCTGCGCGCTCGCTCGCTCACTGAGGCCGCCCGGGCAAAGCCCGGGCGTCGGGCGACCTTT
GGTCGCCCGGCCTCAGTGAGCGAGCGAGCGCGCAGAGAGGGAGTGGCCAACTCCATCACTAGG
GGTTCCTTGTAGTTAATGATTAACCCGCCATGCTACTTATCTACGTAGCCATGCTCTAGGAAGATC
GGAATTCGCCCTTAAGCTAGCGGGCCCCAGAAGCCTGGTGGTTGTTTGTCCTTCTCAGGGGAAA
AGTGAGGCGGCCCCTTGGAGGAAGGGGCCGGGCAGAATGATCTAATCGGATTCCAAGCAGCTC
AGGGGATTGTCTTTTTCTAGCACCTTCTTGCCACTCCTAAGCGTCCTCCGTGACCCCGGCTGGGA
TTTAGCCTGGTGCTGTGTCAGCCCCGGTCTCCCAGGGGCTTCCCAGTGGTCCCCAGGAACCCTC
GACAGGGCCCGGTCTCTCTCGTCCAGCAAGGGCAGGGACGGGCCACAGGCCAAGGGCACCGG
TCATCTGACCACCTGCGAATTTTTGCATCCTGCTGTTTAATCTGCGTTGGTTTAACCGCCTGTCTG
GCTTTTTTTCACTGATGTGATCGCCTTGATGCACTTCTAGAGGATCCCAACGAGTCTTTGTCATC
TACAGGTAGAACCATCATCATGTCCACTCACCACATGGACGAGGCCGACCTCCTTGGGGACCGC
ATTGCCATCATTGCCCAGGGAAGGCTCTACTGCTCAGGCACCCCACTCTTCCTGAAGAACTGCTT
TGGCACAGGCTTGTACTTAACCTTGGTGCGCAAGATGAAAAACATCCAGAGCCAAAGGAAAGGC
AGTGAGGGGACCTGCAGCTGCTCGTCTAAGGGTTTCTCCACCACGTGTCCAGCCCACGTCGATG
ACCTAACTCCAGAACAAGTCCTGGATGGGGATGTAAATGAGCTGATGGATGTAGTTCTCCACCAT
GTTCCAGAGGCAAAGCTGGTGGAGTGCATTGGTCAAGAACTTATCTTCCTTCTTCCAAATAAGAA
CTTCAAGCACAGAGCATATGCCAGCCTTTTCAGAGAGCTGGAGGAGACGCTGGCTGACCTTGGT
CTCAGCAGTTTTGGAATTTCTGACACTCCCCTGGAAGAGATTTTTCTGAAGGTCACGGAGGATTC
TGATTCAGGACCTCTGTTTGCGGGTGGCGCTCAGCAGAAAAGAGAAAACGTCAACCCCCGACAC
CCCTGCTTGGGTCCCAGAGAGAAGGCTGGACAGACACCCCAGGACTCCAATGTCTGCTCCCCAG
GGGCGCCGGCTGCTCACCCAGAGGGCCAGCCTCCCCCAGAGCCAGAGTGCCCAGGCCCGCAG
CTCAACACGGGGACACAGCTGGTCCTCCAGCATGTGCAGGCGCTGCTGGTCAAGAGATTCCAAC
ACACCATCCGCAGCCACAAGGACTTCCTGGCGCAGATCGTGCTCCCGGCTACCTTTGTGTTTTTG
GCTCTGATGCTTTCTATTGTTATCCCTCCTTTTGGCGAATACCCCGCTTTGACCCTTCACCCCTGG
ATATATGGGCAGCAGTACACCTTCTTCAGCATGGATGAACCAGGCAGTGAGCAGTTCACGGTACT
TGCAGACGTCCTCCTGAATAAGCCAGGCTTTGGCAACCGCTGCCTGAAGGAAGGGTGGCTTCCG
GAGTACCCCTGTGGCAACTCAACACCCTGGAAGACTCCTTCTGTGTCCCCAAACATCACCCAGCT
GTTCCAGAAGCAGAAATGGACACAGGTCAACCCTTCACCATCCTGCAGGTGCAGCACCAGGGAG
AAGCTCACCATGCTGCCAGAGTGCCCCGAGGGTGCCGGGGGCCTCCCGCCCCCCCAGAGAACA
CAGCGCAGCACGGAAATTCTACAAGACCTGACGGACAGGAACATCTCCGACTTCTTGGTAAAAAC
GTATCCTGCTCTTATAAGAAGCAGCTTAAAGAGCAAATTCTGGGTCAATGAACAGAGGTATGGAG
GAATTTCCATTGGAGGAAAGCTCCCAGTCGTCCCCATCACGGGGGAAGCACTTGTTGGGTTTTTA
AGCGACCTTGGCCGGATCATGAATGTGAGCGGGGGCCCTATCACTAGAGAGGCCTCTAAAGAAA
TACCTGATTTCCTTAAACATCTAGAAACTGAAGACAACATTAAGGTGTGGTTTAATAACAAAGGCT
GGCATGCCCTGGTCAGCTTTCTCAATGTGGCCCACAACGCCATCTTACGGGCCAGCCTGCCTAA
GGACAGGAGCCCCGAGGAGTATGGAATCACCGTCATTAGCCAACCCCTGAACCTGACCAAGGAG
CAGCTCTCAGAGATTACAGTGCTGACCACTTCAGTGGATGCTGTGGTTGCCATCTGCGTGATTTT
CTCCATGTCCTTCGTCCCAGCCAGCTTTGTCCTTTATTTGATCCAGGAGCGGGTGAACAAATCCA
AGCACCTCCAGTTTATCAGTGGAGTGAGCCCCACCACCTACTGGGTGACCAACTTCCTCTGGGA
CATCATGAATTATTCCGTGAGTGCTGGGCTGGTGGTGGGCATCTTCATCGGGTTTCAGAAGAAAG
CCTACACTTCTCCAGAAAACCTTCCTGCCCTTGTGGCACTGCTCCTGCTGTATGGATGGGCGGTC
ATTCCCATGATGTACCCAGCATCCTTCCTGTTTGATGTCCCCAGCACAGCCTATGTGGCTTTATCT
TGTGCTAATCTGTTCATCGGCATCAACAGCAGTGCTATTACCTTCATCTTGGAATTATTTGAGAAT
AACCGGACGCTGCTCAGGTTCAACGCCGTGCTGAGGAAGCTGCTCATTGTCTTCCCCCACTTCT
GCCTGGGCCGGGGCCTCATTGACCTTGCACTGAGCCAGGCTGTGACAGATGTCTATGCCCGGTT

TGGTGAGGAGCACTCTGCAAATCCGTTCCACTGGGACCTGATTGGGAAGAACCTGTTTGCCATG
GTGGTGGAAGGGGTGGTGTACTTCCTCCTGACCCTGCTGGTCCAGCGCCACTTCTTCCTCTCCC
AATGGATTGCCGAGCCCACTAAGGAGCCCATTGTTGATGAAGATGATGATGTGGCTGAAGAAAGA
CAAAGAATTATTACTGGTGGAAATAAAACTGACATCTTAAGGCTACATGAACTAACCAAGATTTAT
CCAGGCACCTCCAGCCCAGCAGTGGACAGGCTGTGTGTCGGAGTTCGCCCTGGAGAGTGCTTT
GGCCTCCTGGGAGTGAATGGTGCCGGCAAAACAACCACATTCAAGATGCTCACTGGGGACACCA
CAGTGACCTCAGGGGATGCCACCGTAGCAGGCAAGAGTATTTTAACCAATATTTCTGAAGTCCAT
CAAAATATGGGCTACTGTCCTCAGTTTGATGCAATTGATGAGCTGCTCACAGGACGAGAACATCT
TTACCTTTATGCCCGGCTTCGAGGTGTACCAGCAGAAGAAATCGAAAAGGTTGCAAACTGGAGTA
TTAAGAGCCTGGGCCTGACTGTCTACGCCGACTGCCTGGCTGGCACGTACAGTGGGGGCAACAA
GCGGAAACTCTCCACAGCCATCGCACTCATTGGCTGCCCACCGCTGGTGCTGCTGGATGAGCCC
ACCACAGGGATGGACCCCCAGGCACGCCGCATGCTGTGGAACGTCATCGTGAGCATCATCAGA
GAAGGGAGGGCTGTGGTCCTCACATCCCACAGCATGGAAGAATGTGAGGCACTGTGTACCCGG
CTGGCCATCATGGTAAAGGGCGCCTTTCGATGTATGGGCACCATTCAGCATCTCAAGTCCAAATT
TGGAGATGGCTATATCGTCACAATGAAGATCAAATCCCCGAAGGACGACCTGCTTCCTGACCTGA
ACCCTGTGGAGCAGTTCTTCCAGGGGAACTTCCCAGGCAGTGTGCAGAGGGAGAGGCACTACAA
CATGCTCCAGTTCCAGGTCTCCTCCTCCCTGGCGAGGATCTTCCAGCTCCTCCTCTCCCACA
AGGACAGCCTGCTCATCGAGGAGTACTCAGTCACACAGACCACACTGGACCAGGTGTTTGTAAAT
TTTGCTAAACAGCAGACTGAAAGTCATGACCTCCCTCTGCACCCTCGAGCTGCTGGAGCCAGTC
GACAAGCCCAGGACTAACCTGCAGGGCAGTGAAAAAAATGCTTTATTTGTGAAATTTGTGATGCT
ATTGCTTTATTTGTAACCATTATAAGCTGCAATAAACAAGTTGCGGCCGCGTCGAGTTAAGGGCG
AATTCCCGATAAGGATCTTCCTAGAGCATGGCTACGTAGATAAGTAGCATGGCGGGTTAATCATT
AACTACAAGGAACCCCTAGTGATGGAGTTGGCCACTCCCTCTCTGCGCGCTCGCTCGCTCACTG
AGGCCGGGCGACCAAAGGTCGCCCGACGCCCGGGCTTTGCCCGGGCGGCCTCAGTGAGCGAG
CGAGCGCGCAGCTGGGCCTCAGTGAGCGAGCGAGCGCGCAGCTGCATTAATGAATCGGCCAAC
GCGCGGGGAGAGGCGGTTTGCGTATTGGGCGCTCTTCCGCTTCCTCGCTCACTGACTCGCTGC
GCTCGGTCGTTCGGCTGCGGCGAGCGGTATCAGCTCACTCAAAGGCGGTAATACGGTTATCCAC
AGAATCAGGGGATAACGCAGGAAAGAACATGTCGCGTTGCTGGCGTTTTTCCATAGGCTCCGCC
CCCCTGACGAGCATCACAAAAATCGACGCTCAAGTCAGAGGTGGCGAAACCCGACAGGACTATA
AAGATACCAGGCGTTTCCCCCTGGAAGCTCCCTCGTGCGCTCTCCTGTTCCGACCCTGCCGCTT
ACCGGATACCTGTCCGCCTTTCTCCCTTCGGGAAGCGTGGCGCTTTCTCATAGCTCACGCTGTAG
GTATCTCAGTTCGGTGTAGGTCGTTCGCTCCAAGCTGGGCTGTGTGCACGAACCCCCCGTTCAG
CCCGACCGCTGCGCCTTATCCGGTAACTATCGTCTTGAGTCCAACCCGGTAAGACACGACTTATC
GCCACTGGCAGCAGCCACTGGTAACAGGATTAGCAGAGCGAGGTATGTAGGCGGTGCTACAGA
GTTCTTGAAGTGGTGGCCTAACTACGGCTACACTAGAAGAACAGTATTTGGTATCTGCGCTCTGC
TGAAGCCAGTTACCTTCGGAAAAAGAGTTGGTAGCTCTTGATCCGGCAAACAAACCACCGCTGGT
AGCGGTGGTTTTTTTGTTTGCAAGCAGCAGATTACGCGCAGAAAAAAAGGATCTCAAGAAGATCC
TTTGATCTTTTCTACGGGGTCTGACGCTCAGTGGAACGAAAACTCACGTTAAGGGATTTTGGTCAT
GACTGTGGAATGTGTGTCAGTTAGGCGACATAGGTGATCTATGTAGAAGCCTAGTGGAACAGGTT
AGTTTGAGTAGCTTTAGAATGTAAATTCTGGGATCATAGTGTAGTAATCTCTAATTAACGGTGACG
GTTTGTAAGACAGGTCTTCGCAAAATCAAGCGGCAGGTGATTTCAACAGATTCTTGCTGATGGTTT
AGGCGTACAATGCCCTGAAGAATAAGTAAGAGAATAGCACTCCTCGTCGCCTAGAATTACCTACC
GGCGTCCACCATACCTTCGATTATCGCGCCCACTCTCCCATTAGTCGGCACAGGTGGATGTGTTG
CGATAGCCCGCTAAGATATTCTAAGGCGTAACGCAGATGAATATTCTACAGAGTTGCCATAGGCG
TTGAACGCTTCACGGACGATAGGAATGTTGCGTATAGAGCGTGAGTCATCGAAGTGGTGTATACA
CTCGTACTTAACATCTAGCCCGGCTCTATCAGTACACCAGTGCCTTGAATGACATACTCATCATTA
AACTTTCTCAACAGTCAAACGACCAAGTGCATTTCCAAGGAGTGCGAAGGAGATTCATTCTCTCG
CCAGCACTGTAATAGGCACTAAAAGAGTGAAGATAAGCTAGAGTGCCGTGCTAAGACGGTGTCG
GAACAAAGCGGTCTTACGGTCAGTCGTATTTCCTGTCGAGTCCCGTCCAGTTGAGCGTATCACTC
CCAGTGTACTAGCAAGCCGAGAAGGCTGTGCTTGGAGTCAATCGGATGTAGGATGGTCTCCAGA
CACCGGGCCACCACTCTTCACGCCTAGAAGCATAGAACGTCGAGCAGACATCAAAGTCTTAGTAC
CGGACGTGCCGTTTCACTGCGAATATTACCTGAAGCTGTACCGTTATTGCGGAGCAAAGTGACAG
TGCTGCTCTTATCATATTTGTATTGACGACAGCCGCCTTCGCGGTTTCCTCAGACTCTAGATCGAA
TACAGGCTTATTGTAGGCAGAGGCACGCCCTTGTTAGTGGCTGCGGCAATATCTTCCGATCCCCT
TGTCTAACCATGAATCAATTCTCTCATTTGAAGACCCTAATATGTCATCATTAGTGTTTCAAATGCC
ACCAAATACCGCCTAGAAATGTCTATGATGTGTGTCCACTAGAAGTTGATTCACAAACGACTGCTA
GAATCGCGTGATAGGGCATCTTGAAGTTTACATTGTTGTATCGCAAGGTACTCCGATCTTAATGGA

TGCGAAGTGGTACGGATGCAATCAAGCGCGTGAGAGCGGTACATTAGAGCGTTCACCTACGCTA
CGCTAACGGGCGATTCTGATAAGAATGCACATTGCGTCGATTCATAAGATGTCTCGACCGCATGC
GCAACTTGTGAAGTGTCTACTATCCCTAAGCGCATATCTCGCACAGTAACCGAATATGTCGGCAT
CTGATGTTACCGTTGAGTTAGTGTTCAGCTCACGGAACTTATTGTATGAGTAGAGATTTGTAAGAG
CTGTTAGTTAGCTCGCTCAGCTAATAGTTGCCCACACAACGTCAAATTAGAGAACGGTCGTAACA
TTATCGGTGGTTCTCTAACTACTATCAGTACCCACGACTCGACTCTGCCGCAGCTAGGTATCGCC
TGAAAGCCAGTCAGCGTTAAGGAGTGCTCTGACCAGGACAACAGGCGTAGTGAGAGTTACTTGT
TCGTTGCTCTTCCGACTCGGACCTGAGTTCGCCAACGACCCACTTGAGGTCTGAGCCGGTGAAG
AGAAGTAAGCATCTCGTTCGCAGCTTGCCAGCACTTTCAGAACATGACCCCTATTTGTTTATTTTT
CTAAATACATTCAAATATGTATCCGCTCATGAGACAATAACCCTGATAAATGCTTCAATAATATTGA
AAAAGGAAGAGTGGCCGCCTCGGCCTAGGCTTTTGCAAAGATCGATCAAGAGACAGGATGAGGA
TCGTTTCGCATGATTGAACAAGATGGATTGCACGCAGGTTCTCCGGCCGCTTGGGTGGAGAGGC
TATTCGGCTATGACTGGGCACAACAGACAATCGGCTGCTCTGATGCCGCCGTGTTCCGGCTGTC
AGCGCAGGGGCGCCCGGTTCTTTTTGTCAAGACCGACCTGTCCGGTGCCCTGAATGAACTGCAA
GACGAGGCAGCGCGGCTATCGTGGCTGGCCACGACGGGCGTTCCTTGCGCAGCTGTGCTCGAC
GTTGTCACTGAAGCGGGAAGGGACTGGCTGCTATTGGGCGAAGTGCCGGGGCAGGATCTCCTG
TCATCTCACCTTGCTCCTGCCGAGAAAGTATCCATCATGGCTGATGCAATGCGGCGGCTGCATAC
GCTTGATCCGGCTACCTGCCCATTCGACCACCAAGCGAAACATCGCATCGAGCGAGCACGTACT
CGGATGGAAGCCGGTCTTGTCGATCAGGATGATCTGGACGAAGAGCATCAGGGGCTCGCGCCA
GCCGAACTGTTCGCCAGGCTCAAGGCGAGCATGCCCGACGGCGAGGATCTCGTCGTGACCCAT
GGCGATGCCTGCTTGCCGAATATCATGGTGGAAAATGGCCGCTTTTCTGGATTCATCGACTGTGG
CCGGCTGGGTGTGGCGGACCGCTATCAGGACATAGCGTTGGCTACCCGTGATATTGCTGAAGAG
CTTGGCGGCGAATGGGCTGACCGCTTCCTCGTGCTTTACGGTATCGCCGCTCCCGATTCGCAGC
GCATCGCCTTCTATCGCCTTCTTGACGAGTTCTTCTGAGGTACCATGATGCGTGCATGGTAGAAT
GACTCTTGATAACGGACTTCGACTAGGCAATATCCCTTGTCAACTTGTCGAGGAGAAAAGTATTG
ACTGAAGCGCTCCCGGCACAACGGCCAAAGAAGTCTCAGCAATGTTCTTATTTCCGAATGACATG
CGTCTCCTTGCGGGTAAATCGCCGACCGCAAAACTTAGGAGCCAGGATACAGATAGGTCTAACTT
AGGTTAAGGGAGTAAATCCTGGGATCGTTCAGTTGTAACCATATACTTACGCTGGGGCTTCTCCG
GCGGATGTTACTGTCACCAACCACGAGATTTGAAGTAAACGCATGATTGAGCACATAGCCGCGCT
ATCCGACAATCTCCAAATTGATAACATACCGTTCCATGAAGGCCAGAATTACTTACCGGCCCTTTC
CATGCGTGCGCCATACCGCACTCTGCGCTTATCCGTCCGAGGGGAGAGTGTGCGATCCTCCGTT
AAGATATTCTCACGTATGACGTAGCTATGTATTGTGCAGAGGTAGCGAAGGCGTTGAACACTTCA
CAGATGGTGGGGATTCGGGCAAAGGGCGTGATAACTTGGGGACTAACATAGGCGTAAACTACGA
TGGCACCAACTCAATCGCAGCTCGTGCGCCCTGAATCAACGTACTCATCTCAACTGATTCTCGGC
AATCTACGGAGCGACTTGATTATCAACACCTGTCTAGCAGTTCTAATCTTCTGCCAACATCGTACA
TAGCCTCCAAGAGATTATCATACCTATCGGCACAGAAGTGACACGACGCCGAAGGGTAGCGGAC
TTCTGGTCAACCACAATTCCCCAGGGGACAGGTCCTGCGGTGCGCATCACTTTGTAAGTGCAAG
CAACCCAAGTGAGCCCAGCCTGGACTGAGCTGGTTCCTGTGTCAGGTCGAGGCTGGGGATGAC
AGCTCTTGTAAACATAGTGATCAAGCGTGGCGTCGAACGGTCGAGAAACTCATAGTACCTCGGGT
AGCAACTTACTCAGGTTATTGCTTGAAGCTGTACTATTTCAGGAGCGCTGAAGGTCTCTTCTTCTG
TAGACTGAACTCGCAAGGGTCGTGAAGTCGGTTCCTTCAATGCTTAACAAGAACAAAGGCTTACT
GTGCAGACTGGAACGCCCATCTAGCGGCTCGCGTCTTGAATGCTCGGTCCCCTTTGTCATTGCG
GATACAATCCATTTCCCTCATTCACCAGCTTGCGAAGTCTACATTGAGTAGACGAATGCGACCTA
GAAGAGGTGCGCTTCAGAACTTGTGAGGAGTGGTTGATGCTCTATACTCCATTTGGTGTTTCGTG
CATCACCGCGATAGGCTGACAAGAGGTCTTGAACATTGAATAGCAAGGCACTTCCGGTCTCATAG
AAGAGAGCACGGGATAAGGTACGCGCGTGGTACGGGAGGATCAAGGGGCTACACGATAGAAAG
GCTTCTCCCTCACTCGCTAGGAGGCAAATGCAGAACGCTGGTTACTACTACGATACGTGAAACTT
GTCCAACGGTTGCCCAAAGTGTTAAGTGTCTATCACCCTAGTGCCGTTTCCCGGAGAAAACGCCA
GGTTGAATCCGCATTTGAAGCTACGATGGTGAAGTCTGGGTCGAGCGCGCCGCATGTTGATTGC
GTGAGTAGGCTCGACCAAGAACCGCTAGTAGCGTCGCTGTAGAAATAGTTCTCGACAGACCGTC
GAGTTTAGAAAATGGTAGCAGCATTGTTCGCATCTCAATCAAGTATGGATTACGGTGTTTACACTG
TCCTGCGGCTACCCATCGCCTGAAATCCAGCTCGTGTCAAGCCATTGCCTCTCCGGGACGCCGC
ATGAAGTAACTACATATACCTTGCACGGGTTGACTGCGGTCCGTTCAGACTCGACCAAGGACACA
ATCCAGCGATCGGTGCGGGCCTCTTCGCTATTACGC
