# Supplementary material for: mRNA trans-splicing dual AAV vectors for (epi)genome editing and gene therapy
Source: Nat Commun. 2023 Oct 18;14:6578. doi: 10.1038/s41467-023-42386-0 (PMC10584818; doi:10.1038/s41467-023-42386-0)
Supplement: Supplementary file 2 — Reporting Summary [file 41467_2023_42386_MOESM2_ESM.pdf]

nature portfolio

Corresponding author(s): Elvir Becirovic

Last updated by author(s): Sep 21, 2023

Reporting Summary

Nature Portfolio wishes to improve the reproducibility of the work that we publish. This form provides structure for consistency and transparency in reporting. For further information on Nature Portfolio policies, see our Editorial Policies and the Editorial Policy Checklist.

Statistics

For all statistical analyses, confirm that the following items are present in the figure legend, table legend, main text, or Methods section.

n/a	Confirmed	
☐	☒	The exact sample size (*n*) for each experimental group/condition, given as a discrete number and unit of measurement
☐	☒	A statement on whether measurements were taken from distinct samples or whether the same sample was measured repeatedly
☐	☒	The statistical test(s) used AND whether they are one- or two-sided<br>*Only common tests should be described solely by name; describe more complex techniques in the Methods section.*
☒	☐	A description of all covariates tested
☐	☒	A description of any assumptions or corrections, such as tests of normality and adjustment for multiple comparisons
☐	☒	A full description of the statistical parameters including central tendency (e.g. means) or other basic estimates (e.g. regression coefficient) AND variation (e.g. standard deviation) or associated estimates of uncertainty (e.g. confidence intervals)
☐	☒	For null hypothesis testing, the test statistic (e.g. *F*, *t*, *r*) with confidence intervals, effect sizes, degrees of freedom and *P* value noted<br>*Give P values as exact values whenever suitable.*
☒	☐	For Bayesian analysis, information on the choice of priors and Markov chain Monte Carlo settings
☒	☐	For hierarchical and complex designs, identification of the appropriate level for tests and full reporting of outcomes
☒	☐	Estimates of effect sizes (e.g. Cohen's *d*, Pearson's *r*), indicating how they were calculated

Our web collection on statistics for biologists contains articles on many of the points above.

Software and code

Policy information about availability of computer code

Data collection	To predict the strength of different splice sites, NNSplice (Berkeley Drosophila Genome Project, http://www.fruitfly.org/seq_tools/splice.html) and the alternative splice site predictor (ASSP, http://wangcomputing.com/assp/) were used.<br>sgRNAs and pegRNAs were designed using the CRISPOR (http://crispor.tefor.net/) or pegFinder (http://pegfinder.sidichenlab.org/) software, respectively.<br>The western blots were imaged using the ImageLab software (Bio-Rad) or VisionWorks LS Analysis Software (ABCA4, Analytik Jena).<br>Luminescence measurements were performed with the Living Image® 4.7.4 software (Perkin Elmer).<br>Stereotactic injections were performed using the Neurostar software (Neurostar GmbH).<br>OCT measurements were performed using the Heidelberg Eye Explorer version 1.10.4.0 software (Heidelberg Engineering).<br>Confocal images were obtained using the LASX software (Leica).
Data analysis	qRT-PCR results were analyzed using the QuantStudioTM Design & Analysis software (Thermo Fisher Scientific).<br>The western blots relative band intensities were quantified using the ImageLab software (Bio-Rad, v5) or VisionWorks LS Analysis Software (ABCA4, Analytik Jena).<br>Luminescence data was analysed using the Living Image® 4.7.4 software (Perkin Elmer).<br>For RNAseq Trimmomatic v.0.36 was used to trim the sequence reads for adapter sequences and quality. The reads were mapped to the Mus musculus GRCm38 reference genome (Ensembl) using the STAR aligner v.2.5.2b. Unique gene hit counts were calculated using FeatureCounts from the Subread package v.1.5.2 and only unique reads that fell into exonic regions were included. DESeq2 was used to analyze the obtained gene hit counts.<br>Deconvolution and maximum intensity z-projection of images from ABCA4 immunostainings were performed in the ZEISS Zen Blue 3.3 Software. To analyze the images acquired by confocal microscopy Fiji (https://fiji.sc, 1.54f), an open-source image processing software, was

used.
Plotting and statistical analysis were performed with GraphPad Prism 9 (GraphPad Software, v10.0.2).

For manuscripts utilizing custom algorithms or software that are central to the research but not yet described in published literature, software must be made available to editors and reviewers. We strongly encourage code deposition in a community repository (e.g. GitHub). See the Nature Portfolio guidelines for submitting code & software for further information.

Data

Policy information about availability of data

All manuscripts must include a data availability statement. This statement should provide the following information, where applicable:

- Accession codes, unique identifiers, or web links for publicly available datasets
- A description of any restrictions on data availability
- For clinical datasets or third party data, please ensure that the statement adheres to our policy

All source data are provided as a Source Data file. The datasets generated in this study are available in public repositories. The RNA-Seq data have been deposited to GEO (Gene Expression Omnibus, https://www.ncbi.nlm.nih.gov/geo/) under the GEO accession GSE198893. The NGS data from the prime editing experiments have been also deposited to GEO under the GEO accession GSE198863. All final REVeRT sequence elements used in this study are listed in Table S2. Full sequences of the final constructs used for gene therapy experiments are provided in the supplementary information. Additionally, sgRNAs, pegRNAs and primers used in this study can be found in Table S5, S6 and S7. The gating strategy used in this study is displayed in Fig. S1. The Mus musculus GRCm38 reference genome can be accessed via Ensembl (http://nov2020.archive.ensembl.org/Mus_musculus/Info/Index).

Research involving human participants, their data, or biological material

Policy information about studies with human participants or human data. See also policy information about sex, gender (identity/presentation), and sexual orientation and race, ethnicity and racism.

Reporting on sex and gender	N/a
Reporting on race, ethnicity, or other socially relevant groupings	N/a
Population characteristics	N/a
Recruitment	N/a
Ethics oversight	N/a

Note that full information on the approval of the study protocol must also be provided in the manuscript.

Field-specific reporting

Please select the one below that is the best fit for your research. If you are not sure, read the appropriate sections before making your selection.

☒ Life sciences ☐ Behavioural & social sciences ☐ Ecological, evolutionary & environmental sciences

For a reference copy of the document with all sections, see nature.com/documents/nr-reporting-summary-flat.pdf

Life sciences study design

All studies must disclose on these points even when the disclosure is negative.

Sample size	Sufficient sample sizes were chosen to determine whether the outcome was statistically significant. At least three biologically independent replicates were used to meet the minimal requirements for statistical analysis.<br>Effort was taken to keep the number of animals at a minimum.
Data exclusions	No data was excluded.
Replication	At least 3 biological replicates were used for each experiment.<br>All attempts at replication were successful.
Randomization	All animals and cells were randomly assigned to the experimental or the control group.
Blinding	All experiments were not blinded.

Reporting for specific materials, systems and methods

We require information from authors about some types of materials, experimental systems and methods used in many studies. Here, indicate whether each material, system or method listed is relevant to your study. If you are not sure if a list item applies to your research, read the appropriate section before selecting a response.

Materials & experimental systems

n/a	Involved in the study
☐	☒ Antibodies
☐	☒ Eukaryotic cell lines
☒	☐ Palaeontology and archaeology
☐	☒ Animals and other organisms
☒	☐ Clinical data
☒	☐ Dual use research of concern
☒	☐ Plants

Methods

n/a	Involved in the study
☒	☐ ChIP-seq
☐	☒ Flow cytometry
☒	☐ MRI-based neuroimaging

Antibodies

Antibodies used

Living Colors A.v. Monoclonal Antibody (JL-8), mouse α-GFP/Cerulean (1:2000): supplier: Clontech, Takara, catalog number: 632380, monoclonal antibody (JL-8)

β-Tubulin (D3U1W) Mouse mAb (1:3000): supplier: Cell Signaling Technology, catalog number: 86298. monoclonal antibody (D3U1W)

Anti-Myo7b antibody produced in rabbit (1:1000): supplier: Sigma-Aldrich, catalog number: HPA039131, polyclonal antibody

Anti-β-Actin–Peroxidase antibody, Mouse monoclonal, clone AC-15 (1:2000): supplier: Sigma-Aldrich, catalog number: A3854, monoclonal antibody (AC-15)

Anti-ABCA4 (1:1000): supplier: abcam, catalog number: ab72955, polyclonal antibody

Rabbit anti-hexokinase II antibody, C64G5 (1:1000): supplier: Cell Signaling, catalog number: XXX, monoclonal antibody (C64G5)

Peroxidase AffiniPure Donkey Anti-Rabbit IgG (H+L) (1:2000): Jackson ImmunoResearch, catalog number 711-035-152, polyclonal antibody

goat anti-mouse IgG-HRP (1:2000): Santa Cruz Biotechnology, catalog number sc-2005, polyclonal antibody

Rabbit anti-opsin red/green (M-opsin) antibody (1:300), supplier: Merck, catalog number: AB5405, polyclonal antibody

Mouse anti-Rhodopsin Antibody, CT, last 9 amino acids (1:2000), supplier: Sigma Aldrich, catalog number: MAB5356, clone Rho 1D4

Anti-ABCA4 Antibody (3F4) (1:100): supplier: Santa Cruz Biotechnology, catalog number: sc-65672, monoclonal antibody (3F4)

Rabbit anti-CNGB1 antibody (1:5000): supplier: custom made, polyclonal antibody

Cy™3 AffiniPure Donkey Anti-Rabbit IgG (H+L) (1:400): supplier: Jackson ImmunoResearch, catalog number: 711-165-152, polyclonal antibody

Cy™5 AffiniPure Goat Anti-Mouse IgG (H+L) (1:400): supplier: Jackson ImmunoResearch, catalog number: 115-175-146, polyclonal antibody

Alexa Fluor Plus 488 Donkey anti-Mouse: supplier (1:800): Thermo Fisher Scientific, catalog number: A32766, polyclonal antibody

Validation

Living Colors A.v. Monoclonal Antibody (JL-8): validated by manufacturer.

β-Tubulin (D3U1W) Mouse mAb (1:3000): The antibody was validated by the manufacturer by western blot analysis of extracts from HeLa, NIH/3T3, KNRK cells and rat brain.

Anti-Muo7b antibody: The antibody was validated by the manufacturer for Immunohistochemistry by stainings of duodenum and liver tissues.

Anti-β-Actin–Peroxidase antibody: The antibody was validated by the manufacturer by western blot analysis using cell extracts of human foreskin fibroblasts or chicken fibroblasts.

Anti-ABCA4: The antibody was validated by the manufacturer by western blot analysis using lysates of human eye and murine retina tissue

Rabbit anti-hexokinase II antibody: validated by the manufacturer.

Peroxidase AffiniPure Donkey Anti-Rabbit IgG (H+L): validated by manufacturer.

goat anti-mouse IgG-HRP: validated by manufacturer.

Rabbit anti-opsin red/green (M-opsin) antibody: The antibody was validated by the manufacturer for Immunohistochemistry by stainings of mouse retina tissue.

Mouse anti-Rhodopsin Antibody, CT, last 9 amino acids: The antibody was validated by the manufacturer by western blot using isolated bovine rod outer segment and immunohisto/cytochemistry with fixed frozen tissue sections.

Anti-ABCA4 Antibody (3F4): The antibody was validated by the manufacturer by Immunoperoxidase staining of formalin fixed, paraffin-embedded human fetal eye tissue

Rabbit anti-CNGB1 antibody: validated by manufacturer.

Cy™3 AffiniPure Donkey Anti-Rabbit IgG (H+L): validated by manufacturer.

Cy™5 AffiniPure Goat Anti-Mouse IgG (H+L): validated by manufacturer.

Alexa Fluor Plus 488 Donkey anti-Mouse: validated by manufacturer.

Eukaryotic cell lines

Policy information about cell lines and Sex and Gender in Research

Cell line source(s)	661W cells were kindly provided by Prof. Muayyad Al-Ubaidi, University of Housten, US.<br>HEK293 cells were ordered from DSMZ - German Collection of Microorganisms and Cell Cultures GmbH (cat. no. ACC305).<br>HEK293T cells were ordered from DSMZ - German Collection of Microorganisms and Cell Cultures GmbH (cat. no. ACC635).<br>Mouse embryonic fibroblasts (MEFs) were generated as described in Xu J. Preparation, culture, and immortalization of mouse embryonic fibroblasts. Curr Protoc Mol Biol. 2005 May;Chapter 28, doi: 10.1002/0471142727.mb2801s70.
Authentication	661W: cell lines were not authenticated.<br>HEK293, HEK293T: DSMZ states that they comprehensively perform authentication (Short Tandem Repeat profiling) and quality control tests on all distribution lots of cells lines. In cell culture the HEK293 and HEK293T cells had shown typical morphology and cell growth.<br>MEFs: cell lines were not authenticated.
Mycoplasma contamination	Cell lines were not tested for mycoplasma contamination.
Commonly misidentified lines<br>(See ICLAC register)	No commonly misidentified cell lines were used.

Animals and other research organisms

Policy information about studies involving animals; ARRIVE guidelines recommended for reporting animal research, and Sex and Gender in Research

Laboratory animals	Rho+/P23H (B6.129S6(Cg)-Rhotm1.1Kpal/J, Jackson Laboratory, RRID:IMSR_JAX:017628) mice and Abca4-/- Rdh8-/- (B6;129-Abca4tm1Ght Rdh8tm1Kpal/J, Jackson Laboratory, RRID:IMSR_JAX:030503) mice were initially bought from the Jackson Laboratory. Rho P23H/+ and C57BL/6J WT mice were obtained by in-house breeding and maintained on a C57BL/6J background. Abca4-/- Rdh8-/- mice homozygous for the Leu450 polymorphism in Rpe65 were obtained by in-house breeding. Mice were kept in a 12-hour dark/light cycle at an ambient temperature of 22 °C, and 60 % humidity.<br><br>Experiments were performed with both female and male mice.<br><br>Age of mice used in the animal experience:<br>Hippocampal primary neuron preperation: Neurons were isolated from mice hippocampi at p0.<br>Subretinal injection: Injection at p21 for C57BL/6J and Rho P23H/+ mice. Retina isolation for qRT-PCR, WB and IHC 4 weeks after injection. Subretinal Injection for ABCA4-/- Rdh8 -/- mice was performed at p14. ERG and OCT measurements were performed 4 and 10 weeks post injection. Retinas were isolated for WB and IHC 10 weeks post injection.<br>Hippocampal injection: Injection was performed at p30. The hippocampi were isolated 4 weeks after injection.<br>Intraperitoneal injection: Injection was performed at p7. For Luciferase experiments imaging and organ isolation was performed 3 weeks after injection. For transactivation experiments the organs were isolated 5 weeks after injection.
Wild animals	No wild animals were used.
Reporting on sex	Sex was not considered in the study design.
Field-collected samples	No field-collected samples were used.
Ethics oversight	All animal procedures were performed with the permission of local authorities (District Government of Upper Bavaria, Germany) in accordance with the German laws on animal welfare (Tierschutzgesetz), and were performed in compliance with widely accepted ethical standards. Effort was taken to keep the number of animals at a minimum.

Note that full information on the approval of the study protocol must also be provided in the manuscript.

Flow Cytometry

Plots

Confirm that:

☒ The axis labels state the marker and fluorochrome used (e.g. CD4-FITC).

☒ The axis scales are clearly visible. Include numbers along axes only for bottom left plot of group (a 'group' is an analysis of identical markers).

☒ All plots are contour plots with outliers or pseudocolor plots.

☒ A numerical value for number of cells or percentage (with statistics) is provided.

Methodology

Sample preparation	Flow cytometry was conducted on HEK293 cells 48 h post transfection. Cells were washed with DPBS (Thermo Fisher Scientific) and detached from the culture plate with TrypLE™ Express (Thermo Fisher Scientific). The cells were collected in culture medium and centrifuged at 100 x g for 5 min. The cell pellet was resuspended in 400 µl FACS buffer (2 % FBS (Sigma), 2 mM EDTA (VWR), 25 mM HEPES (Sigma) in PBS (Sigma)). Immediately before flow cytometry, the cells were separated with a 40 µm strainer (pluriSelect).
Instrument	FACS was performed on a BD LSR FortessaTM (BD Bioscience)
Software	Data were collected with BD FACSDiva Software and analyzed in FlowJo (https://www.flowjo.com).
Cell population abundance	Cell counts were performed using SYTOX Blue Dead Cell staining (Thermo Fisher), after 5 min incubation at room temperature. Cells were counted for each biological replicate.
Gating strategy	Analysis was done with single cell gating hierarchies on FSC and SSC. From this, the cerulean-positive population could be distinguished. For better analysis, live cells were quantified by SYTOX Blue Dead Cell Stain.

☒ Tick this box to confirm that a figure exemplifying the gating strategy is provided in the Supplementary Information.
